# Intermediate acoustic-to-semantic representations link behavioral and neural responses to natural sounds

**DOI:** 10.1038/s41593-023-01285-9

**Published:** 2023-03-16

**Authors:** Bruno L. Giordano, Michele Esposito, Giancarlo Valente, Elia Formisano

**Affiliations:** 1 Institut de Neurosciences de La Timone, UMR 7289, CNRS and Université Aix-Marseille, Marseille, France; 2 Department of Cognitive Neuroscience, Faculty of Psychology and Neuroscience, Maastricht University, Maastricht, the Netherlands; 3 Maastricht Centre for Systems Biology (MaCSBio), Faculty of Science and Engineering, Maastricht University, Maastricht, the Netherlands; 4 Brightlands Institute for Smart Society (BISS), Maastricht University, Maastricht, the Netherlands

**Keywords:** Cortex, Neural encoding, Machine learning, Perception, Sensory processing

## Abstract

Recognizing sounds implicates the cerebral transformation of input waveforms into semantic representations. Although past research identified the superior temporal gyrus (STG) as a crucial cortical region, the computational fingerprint of these cerebral transformations remains poorly characterized. Here, we exploit a model comparison framework and contrasted the ability of acoustic, semantic (continuous and categorical) and sound-to-event deep neural network representation models to predict perceived sound dissimilarity and 7 T human auditory cortex functional magnetic resonance imaging responses. We confirm that spectrotemporal modulations predict early auditory cortex (Heschl’s gyrus) responses, and that auditory dimensions (for example, loudness, periodicity) predict STG responses and perceived dissimilarity. Sound-to-event deep neural networks predict Heschl’s gyrus responses similar to acoustic models but, notably, they outperform all competing models at predicting both STG responses and perceived dissimilarity. Our findings indicate that STG entails intermediate acoustic-to-semantic sound representations that neither acoustic nor semantic models can account for. These representations are compositional in nature and relevant to behavior.

## Abstract (web summary)

The authors compare three classes of models—acoustic, semantic and sound-to-event deep neural network—to determine which can best link specific features of auditory stimuli to predicted functional magnetic resonance imaging responses in auditory cortical regions.

## Main

One of the most important functions of the auditory system is to assist us in recognizing sound-generating objects and events in the acoustic environment (for example, a bird chirping, a car approaching)^1^. Although the functional-neuroanatomical pathway subserving sound processing is well understood^2,3^, our knowledge of how the brain transforms incoming sounds into meaningful semantic representations is less established^4–10^. Over the years, several computational models have been proposed that can be used to describe sound representations at the different stages of this acoustic-to-semantic transformation chain. On the acoustic (input) side, these include biophysically inspired models approximating sound representations at the peripheral, subcortical and early cortical levels^11–13^, and psychophysically informed models of the dimensions of auditory sensation^14–16^. On the semantic (output) side, recent models developed in the context of natural language processing (NLP) derive numerical representations of abstract semantic entities and concepts—the output of sound recognition^17–19^. Finally, end-to-end deep neural networks (DNNs) have been trained on large datasets of human-labeled sounds to map the acoustic input (waveform, spectrogram) into predefined sets of semantic categories^9,20^.

A typical approach to assess the validity of computational models of sound representation is to evaluate their ability to explain experimental—behavioral and/or neural—observations from human listeners. Using this approach, researchers demonstrated that a model accounting for acoustic modulations at different spectrotemporal scales (Chi et al.^13^, referred to here as the modulation transfer function (MTF) model) explains accurately functional magnetic resonance imaging (fMRI) response patterns to natural sounds in Heschl’s gyrus (HG) and early auditory areas^8,10^. These studies also showed that MTF model predictions are less accurate for nonprimary auditory areas along the ventral^2^ and dorsal^3^ subdivisions of the superior temporal gyrus/sulcus (STG/STS). These areas receive their input from early auditory areas and have been shown to exhibit preferential responses to predefined categories of natural sounds (for example, speech^21–23^, human and animal vocalizations^24,25^, music^22,23^ and action sounds^4,25^), and thus are likely to play a crucial role in the acoustic-to-semantic transformation of natural sounds. Most investigations so far have interpreted observed STG responses to natural sounds in terms of neuronal selectivity for broad semantic categories (for example, ‘speech’, ‘music’^22^), whereas others have argued for distributed, compositional coding^26^. Thus, the nature of sound representations in STG is debated, and the question of how semantic representations are derived from earlier stages remains unanswered.

Recently, Kell et al.^9^ showed that sound representations derived from DNNs, specifically trained to recognize speech or musical genres, can explain fMRI responses to natural sounds in STG better than MTF-derived representations. These results suggest that DNNs may be useful to understand the nature of sound representations in nonprimary auditory areas, and STG in particular. Yet, there remain open questions that need to be addressed to interpret and frame the function of DNNs within current computational auditory neuroscience research, including their comparison with psychoacoustical models of postprimary cerebral representation^6^, semantic embeddings^27^ or categorical models of selective cerebral responses to natural sounds^10,22^.

**Fig. 1:**
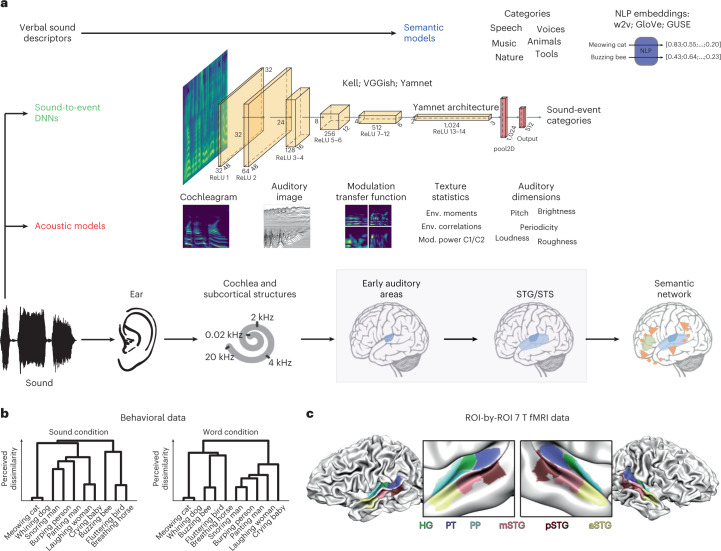
Measuring model representations in behavioral and fMRI data. **a**, Conceptual depiction of the sound representation models considered in this study. Models are divided in three classes, acoustic, sound-to-event DNNs and semantic, and are arranged along a continuum that emphasizes their relationship with the cerebral sound processing hierarchy (regions considered in this study are highlighted). NLP, natural language processing; w2v, Google News word2vector; Env., envelope; Mod., modulation. **b**, Sketch of the behavioral data considered in this study, measuring the perceived dissimilarity of sound stimuli or of words (name plus verb sentences) describing the corresponding sound-generating events (data from Giordano et al.^30^). **c**, Visual depiction of the ROIs considered for the analysis of sound representation models in the brain (data from Santoro et al.^8^).

**Fig. 2:**
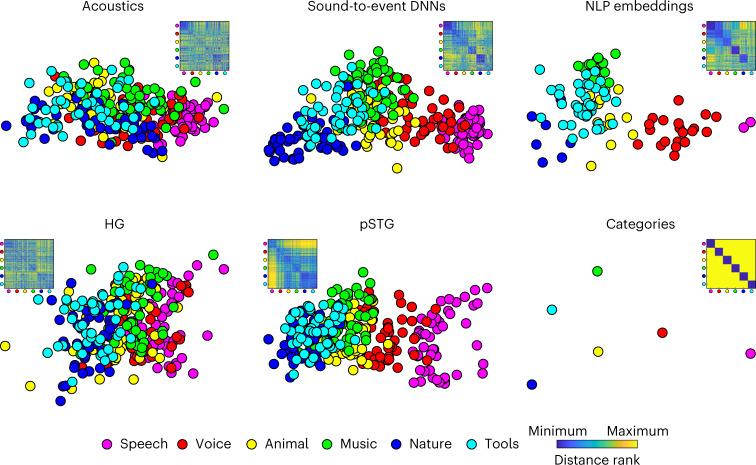
Visualizing acoustic-to-semantic representations in computational models and in the brain. Top, metric MDS of the distance between stimuli in acoustic, sound-to-event DNNs and NLP models (MDS performed on the standardized distance averaged across all model components; for example, all layer-specific distances across all DNNs). Bottom, metric MDS of the distance between stimuli in training-set fMRI data, averaged across CV folds and participants and of the category model. All MDS solutions were Procrustes-rotated to the pSTG MDS (dimensions considered, *N* = 60; only translation and rotation considered). For each MDS solution, we also show the ranked dissimilarity matrix. Note the spatial overlap of category exemplars in the categorical model, postulating zero within-category distances, and the corresponding graded representation of category exemplars in the other models. Note also how pSTG captures the intermediate step of the acoustic-to-semantic transformation emphasized in sound-to-event DNNs. See Supplementary Figs. 2 and 3 for the MDS representation of each model and ROI. fMRI participants, *N* = 5.

**Fig. 3:**
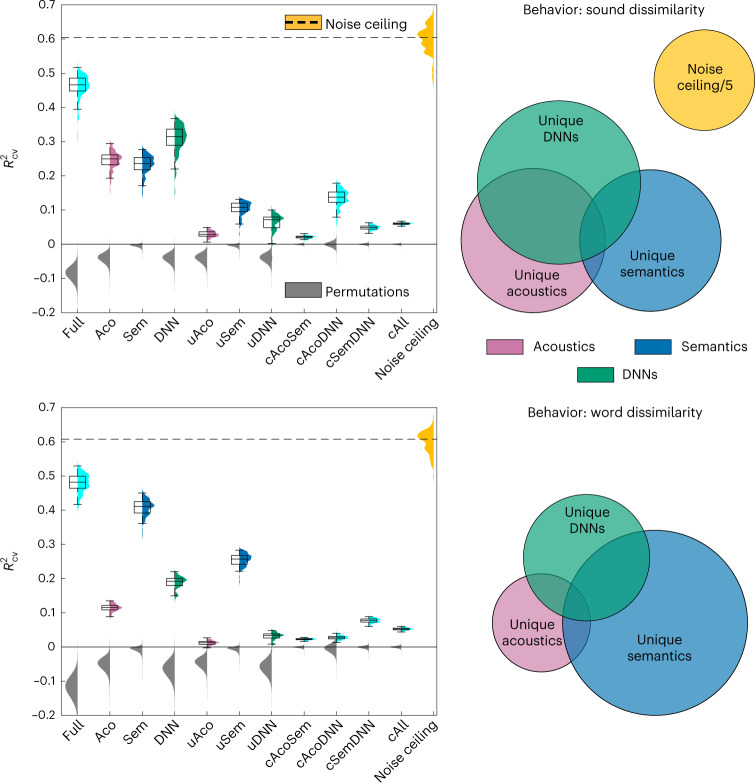
Acoustic and semantic representations in behavioral data. Left, representation analysis by model class. Full, all models together; Aco, acoustic models; Sem, semantic models; DNN, sound-to-event DNNs; u, unique predictive variance component; c, common predictive variance component; cAll, predictive variance component common to the acoustic, semantic and DNN models. Colors represent plugin distribution of *R*^2^_CV_ across CV folds, each with a corresponding box-plot (center, median; lower/upper box limits, first/third quartile; bottom/top whisker, data within 1.5 interquartile ranges from first and third quartiles, respectively); gray, permutation distribution of the across CV-folds median *R*^2^_CV_; orange, distribution of noise ceiling across CV folds (dashed line, median noise ceiling *R*^2^_CV_). Right, Euler diagram representation of the unique and common variance components predicted by acoustic, semantic and DNN models. Top, perceived sound dissimilarity task. Bottom, perceived word dissimilarity task. See Supplementary Fig. 4 for model-by-model analyses, and Supplementary Tables 1 and 2 for numerical results. See Supplementary Figs. 9 and 10, and Supplementary Table 7, for layer-level analyses of DNN representation in perceived sound dissimilarity. Sound or word dissimilarity participants, *N* = 20.

**Fig. 4:**
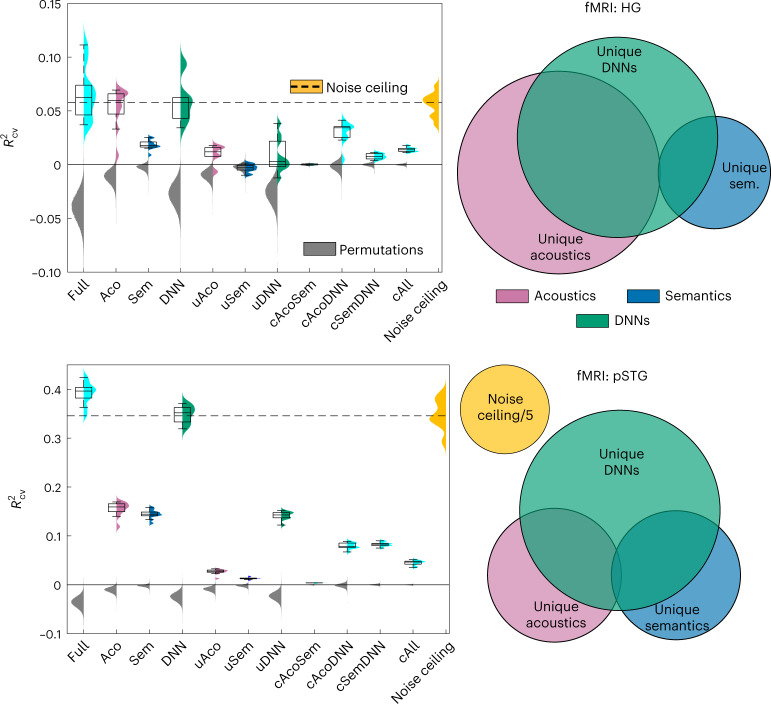
Acoustic and semantic representations in 7 T fMRI data. Left, representation analysis by model class (definitions as in Fig. 3). Colors represent plugin distribution of *R*^2^_CV_ across CV folds, each with a corresponding box-plot (center, median; lower/upper box limits, first/third quartile; bottom/top whisker, data within 1.5 interquartile ranges from first and third quartiles, respectively); gray, permutation distribution of the across CV-folds median *R*^2^_CV_; orange, distribution of noise ceiling across CV folds (dashed line, median noise ceiling *R*^2^_CV_). Right, Euler diagram representation of the unique and common variance components predicted by acoustic, semantic and DNN models. Top, results for the HG ROI. Bottom, results for the pSTG ROI. See Supplementary Figs. 5–8 for a graphical representation of model-by-model and variance partitioning results from all ROIs, and Supplementary Tables 3–6 for numerical results. See Supplementary Figs. 9 and 10, and Supplementary Tables 8–12, for layer-level analyses of DNN representation in fMRI. fMRI participants, *N* = 5.

**Fig. 5:**
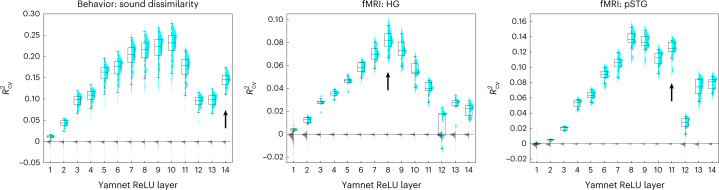
Layer-by-layer analysis of DNN representation in perceived sound dissimilarity and 7 T fMRI. Predictive power (*R*^2^_CV_) of each Yamnet layer. Arrows indicate the top Yamnet layer for which we observed a significant improvement in the predictive power when the layer is added to all previous layers (*P* < 0.05, one-sided, adjusted for MC across layers and fMRI ROIs). Cyan, plugin distribution of *R*^2^_CV_ across CV folds, each with a corresponding box-plot (center, median; lower/upper box limits, first/third quartile; bottom/top whisker, data within 1.5 interquartile ranges from first and third quartiles, respectively); gray, permutation distribution of the across CV-folds median *R*^2^_CV_. See Supplementary Figs. 9 and 10 for results for all DNNs and ROIs and Supplementary Tables 8–12 for numerical results. Sound dissimilarity and fMRI participants, *N* = 20 and *N* = 5, respectively.

**Fig. 6:**
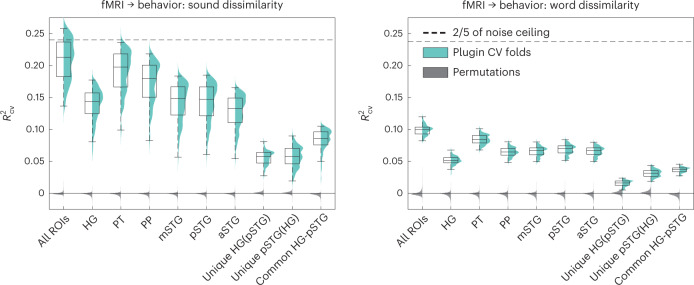
Prediction of behavioral data from 7 T DNN-weighted fMRI data. Left, perceived sound dissimilarity task. Right, perceived word dissimilarity task. Cyan, plugin distribution of *R*^2^_CV_ across CV folds, each with a corresponding box-plot (center, median; lower/upper box limits, first/third quartile; bottom/top whisker, data within 1.5 interquartile ranges from first and third quartiles, respectively); dark gray, cross-CV fold median of the permutation analyses; dashed line, 2/5 (40%) of across-fold median noise ceiling *R*^2^_CV_. Unique, unique behavior-predictive variance; Common, common behavior-predictive variance (HG + STG analysis). See Supplementary Fig. 11 for the same analysis excluding the fMRI speech stimuli and Supplementary Tables 14 and 15 for numerical results. Sound or word dissimilarity participants, *N* = 20.

Here, we address these questions within a systematic model comparison framework, extending representational similarity analysis^28^ into the domain of cross-validated variance partitioning^29^. In particular, we compared numerical predictions of behavioral responses (see Giordano et al.^30^; Experiment 2) and high-field (7 T) fMRI auditory cortical responses to natural sounds (see Santoro et al.^8^; Experiment 2) from models in three classes: acoustic, semantic and sound-to-event DNNs (Fig. 1). We find that both behavioral and fMRI responses (STG) are better predicted by DNNs than acoustic and semantic models (Figs. 2–4). Through a variance partition analysis, we show that DNNs capture predictive acoustic and semantic representations in behavior and in the brain, and that their superiority stems from a representational level that cannot be explained either by acoustic or semantic models or by their combination (Figs. 3 and 4). Additional analyses show that this sound representation level emerges in intermediate layers of the DNNs (Fig. 5). We finally reveal shared DNN representations in fMRI and behavior through a stringent external validation test that generalizes DNNs representations in fMRI data to behavioral data obtained in a different group of participants that carried out a different task, on different sound stimuli (Fig. 6). Overall, our results suggest that common representations, intermediate between acoustics and semantics, subserve behavioral and neural (STG) responses to natural sounds.

## Results

Figure 1 provides a schematic overview of all models (Fig. 1a) and data (Fig. 1b,c) in this study. We considered five biophysically and/or psychophysically inspired acoustic models approximating signal representation at the level of the peripheral auditory system (cochleagram (CochGram^11^)), subcortical auditory centers (stabilized auditory image model (SAI^12^)), primary auditory cortex (modulation transfer function (MTF^13^)), and postprimary auditory cortex (texture model^31^ and auditory dimensions model (AudDims), measuring five time-varying measures of auditory sensation: pitch^14^, loudness^15^, periodicity^32^, timbre brightness^33^ and roughness^16^). We characterized semantic structure with a model describing categorical sensitivities (fMRI) and with multidimensional semantic embeddings (behavior, fMRI) calculated by applying three NLP models to sound labels (single-word based, unsupervised learning (GloVe^18^) and supervised learning (GNewsW2V^17^) and sentence based (Google Universal Sentence Encoder (GUSE^19^)). We finally considered sound-to-event DNNs trained to recognize sound event categories from input sounds: the dual-task word and music genre categorization network by Kell et al.^9^, and the VGGish and Yamnet networks by the Google Research Team^20^, trained to recognize the sound event categories in the AudioSet natural sound taxonomy^34^, and a benchmarking standard in artificial hearing research (Fig. 1a).

Behavioral data consisted of perceived dissimilarities estimated with a hierarchical sorting task^35^, whereby experiment participants (*N* = 40) merged similar stimuli or groups of stimuli until they were all grouped together (*N* stimuli (sounds or sound-identification labels = 80, 40 for each of two categories) living object and nonliving object; median sound duration = 5.1 s) (Fig. 1b). fMRI dissimilarity matrices were obtained from response patterns in six auditory cortical regions of interest (ROIs). Participants (*N* = 5) performed a one-back sound-repetition detection task and listened to 288 sounds (72 for each of six categories: human nonspeech vocal sounds, speech sounds, animal cries, musical instruments, scenes from nature and tool sounds, with sound duration = 1 s) (Fig. 1c).

Figure 2 visualizes, for fMRI stimuli and data, the dissimilarity matrices and their two-dimensional multidimensional scaling (MDS) projections of modeled sound representations (acoustics, DNN, semantics, top row) and of observed response patterns in HG and pSTG, bottom row) (see Supplementary Figs. 2 and 3 for the MDS representation of all models and ROIs, respectively). In these MDS plots, different points indicate different stimuli: the closer two stimuli are in the plot, the more similar their modeled representations (top) or observed responses (bottom). Note that the category of stimuli is used here only for color coding, but it does not influence the analysis otherwise. The MDS plots of stimulus representations by the various model classes (top) highlight the hypothesized acoustic-to-semantic transformation underlying sound recognition. Representations of stimuli from different categories largely overlap in acoustic models (except for speech), partially cluster in DNN models, and more fully cluster to reflect the semantic stimulus categorization in NLP models. Inspection of the MDS plots of observed fMRI responses (bottom) suggests a similar gradual transformation of sound representations in auditory cortex, with stimulus distances resembling those of acoustic models in HG and those of DNNs in STG. Semantic categories may then be read-out from STG representations (for example, in prefrontal cortex) as schematized by the MDS plot of categorical predictors (bottom-right corner). With the analyses described below and in the Supplementary Information materials, we assess statistically these hypotheses and qualitative observations. Furthermore, we investigate the relation between neural and behavioral representations.

Figures 3 and 4 illustrate the ability of the various models to predict behavioral and fMRI response dissimilarities (that is, distances; Supplementary Fig. 1), as assessed using the cross-validated *R*-squared statistic (*R*^2^_CV_; Methods). Models are grouped in three classes: acoustics (red), semantics (blue) and DNNs (green). For the behavioral data, we observed significant predictions by all tested models (*P* ≤ 0.0155, corrected across same-class models, family-wise error rate (FWER) = 0.05; Supplementary Fig. 4 and Supplementary Table 2). In the sound dissimilarity condition, *R*^2^_CV_ was overall higher for DNNs compared with semantic and acoustic models (*P* = 0.042 and 0.018 for the DNN versus semantic or acoustic-model contrasts, respectively; Fig. 3, top-left panel, and Supplementary Table 1). In the control word dissimilarity condition, *R*^2^_CV_ was instead higher for semantic models compared with DNN and acoustic models (*P* = 0.0001; Fig. 3, bottom-left panel, and Supplementary Table 1). In the sound dissimilarity condition, AudDims, Yamnet and GNewsW2V outperformed the other models for the acoustic, DNN and semantic model classes, respectively (*P* = 0.0001 for all relevant pairwise contrasts; Supplementary Fig. 4, left panel, and Supplementary Table 2). GNewsW2V outperformed the competing semantic models also at predicting perceived dissimilarity in the word condition (*P* = 0.0001 for all relevant pairwise contrasts; Supplementary Fig. 4, right panel, and Supplementary Table 2).

The variance-partitioning analysis indicated that the DNNs incorporate a large part of the perceived sound dissimilarity variance predicted also by the acoustic and semantic models as well (*P* value for common acoustic-DNN and semantic-DNN variance = 0.0001), and that the component uniquely explained by semantic models is larger than the unique acoustic component (*P* = 0.006; Fig. 3, top-left panel, and Supplementary Table 1).

For the fMRI data, model prediction results were distinctively dependent on the anatomical ROI (Fig. 4, Supplementary Figs. 5–8 and Supplementary Tables 3–6). In HG, the putative location of primary (core) areas, all model classes predicted a significant portion of the fMRI variance (*P* = 0.0001). However, only acoustic models predicted a unique variance of the fMRI response dissimilarities (*P* = 0.0013; *P* ≥ 0.331, for the unique semantic and DNN explained variances), with a large common component predicted equally by the acoustics and DNN models (*P* = 0.0001; Fig. 4, top-left panel, and Supplementary Table 3). Across the acoustic models, the spectrotemporal modulation representation (MTF) provided the best prediction of HG responses (*P* ≤ 0.0017; Supplementary Fig. 6 and Supplementary Table 4). No DNN model outperformed clearly all others, with Yamnet outperforming Kell’s network, but not VGGish (*P* = 0.0012 and 0.085, respectively).

In the STG regions (posterior/mid/anterior STG: mSTG, pSTG, aSTG), which contain auditory areas at higher processing levels (parabelt), we observed a complementary pattern of results compared with HG. Whereas predictions by the acoustic models and DNNs were comparable in HG, in all STG regions DNN predictions were significantly better than those made by acoustic and by semantic models (*P* = 0.0001, for all relevant contrasts in all STG ROIs; Fig. 4, bottom-left panel, Supplementary Figs. 5–8 and Supplementary Table 3). Categorical predictors explained more variance in STG than in HG and early ROIs (see Supplementary Fig. 3b for category-by-category details and Supplementary Table 16 for numerical results), which is consistent with previous studies^22,23^. DNN models, however, were significantly better than this categorical stimulus description in all STG regions (Supplementary Fig. 6). Importantly, the variance partition analysis showed that DNNs predicted variance that could not be predicted by either acoustic or semantic models (or by their combination; see significant unique contribution of the DNNs, *P* = 0.0001).

Similarly to HG, in STG both Yamnet and VGGish outperformed Kell’s network at predicting brain response similarities (*P* ≤ 0.0012, for all relevant contrasts in all STG ROIs; Supplementary Fig. 6 and Supplementary Table 4). Unlike in HG, semantic and acoustic models predicted a similar portion of variance. In all STG regions, the AudDims model outperformed all others (*P* = 0.0001, for all relevant contrasts in all STG ROIs; Supplementary Fig. 6 and Supplementary Table 4), which is in agreement with the notion that AudDims entails a higher level of acoustic representations (pitch, loudness, brightness and so on) compared with spectrotemporal modulations (MTF). GNewsW2V was the best semantic model in pSTG and mSTG, which is consistent with previous analysis of the behavioral data (*P* ≤ 0.0034).

Overall, these results indicate that intermediate sound representations, such as those emerging in hierarchical DNNs, describe neural sound representations in STG (as measured with fMRI) better than other proposed acoustic (low-level, MTF; high-level, AudDims) or semantic representations (both continuous (word2vec) and discrete categories; see also the detailed analysis of DNNs below). The variance-partitioning analysis further supports this interpretation, showing that the unique DNN component in pSTG and mSTG is significantly larger than the unique acoustic or semantic component (*P* ≤ 0.0021; Fig. 4, bottom-left panel, Supplementary Figs. 5–8 and Supplementary Table 3). In PT and PP regions, which contain nonprimary auditory (belt) regions, results resembled those observed in STG region, including a significant unique contribution to prediction by DNNs (*P* = 0.0001), a clear better performance of Yamnet and VGGish over Kell’s DNN (*P* = 0.0001), of GNewsW2V over the other continuous semantic models (*P* = 0.0001) and an overall better predictivity of AudDims relative to the other acoustic models (Supplementary Figs. 5 and 6 and Supplementary Tables 3 and 4). This suggests that the transformation from acoustic to higher-level sound representations initiates at the level of belt auditory areas.

The original set of stimuli used in the fMRI study included speech as one of the sound categories. Speech sounds differ from the other sounds in terms of low-level acoustic features; furthermore, they evoke significantly higher responses in STG than other sounds^22^. To control that our results were determined not solely by differences in responses to speech stimuli compared with the other sounds, we performed identical analyses of model comparison and variance partitioning after removing the speech stimuli and corresponding responses from the data (Supplementary Figs. 7 and 8 and Supplementary Tables 5 and 6). We observed a reduction of overall response levels and consistency, especially in STG regions (as indicated by the lower noise level; Supplementary Figs. 7 and 8). However, the results of the model comparison and variance partitioning reproduced those obtained with the full set of stimuli, thus confirming the general predominance of DNN models.

Sound-to-event DNNs provided the best account of both perceived sound dissimilarity (but not word dissimilarity), and fMRI responses to natural sounds. We then assessed in detail the predictive power of each layer of these DNNs by performing layer-by-layer predictions (Fig. 5, Supplementary Figs. 9 and 10 and Supplementary Tables 8–12). We observed that, for both VGGish and Yamnet, the most predictive DNNs among those considered here, behavioral and fMRI responses to sounds were best predicted by intermediate layers that bridge the input acoustic stimulus with the output semantic categorization of sound events. A control analysis of a randomly initialized VGGish network failed to replicate this predictivity advantage for intermediate DNN layers (Supplementary Fig. 12). In addition, we also performed a cumulative analysis assessing the DNN-based predictions of responses obtained by incrementally adding layers from the earliest to the latest (Supplementary Fig. 10 and Supplementary Table 9). Interestingly, for fMRI responses, we observed distinct prediction profiles for HG and STG regions, with incremental contribution of the late layers in STG regions but not in HG (Fig. 5, Supplementary Fig. 10 and Supplementary Table 10).

Despite the large differences between behavioral and fMRI data in terms of sound sets, experiment participants and experimental paradigm (dissimilarity judgment versus one-back task), the separate analyses of both datasets gave highly consistent indications of the ability of the different computational models and, in particular, of sound-to-event DNNs, to predict responses to natural sounds. We thus tested the degree of generality and behavioral relevance of the modeled neural representations by carrying out an analysis predicting behavioral perceived sound dissimilarities from the fMRI responses. Mapping the stimuli for both datasets onto a common space of DNN models made this possible (Fig. 6, Supplementary Fig. 11 and Supplementary Tables 14–15). We found that DNN-weighted fMRI from all ROIs together predicted a substantial amount of the variance of the perceived sound dissimilarity (35% of the noise ceiling; *P* = 0.0001; Fig. 6, left panel) and that HG and pSTG contributed unique aspects to the prediction of behavior (*P* = 0.0001). We obtained the same results in a control analysis that excluded the speech fMRI stimuli (Supplementary Fig. 11). A further control analysis revealed a comparatively limited ability of DNN-weighted fMRI data to predict perceived word dissimilarity (Fig. 6, right panel, and Supplementary Fig. 11, right panel).

## Discussion

We investigated the representation of natural sounds through model-based analyses of two largely different datasets obtained with different methodology (behavioral measure versus fMRI), stimuli and paradigms (perceived dissimilarity versus one-back repetition detection) and participants. Both datasets gave highly consistent indications of the ability of existing computational models to predict responses to natural sounds. Among the models we considered, sound-to-event DNNs provided the best overall predictions in both the behavioral and neural datasets (Figs. 3 and 4). Furthermore, projecting fMRI data onto the DNN model space, we could predict a sizeable portion of the behavioral data variance (Fig. 6), indicating that the DNNs capture a representational level common to behavioral and neural responses.

The DNNs considered here are convolutional hierarchical models trained to categorize sound-producing objects and events. As such, they can be considered candidate computational implementations of the acoustic-to-semantic transformations underlying the recognition of everyday sounds. Although a mechanistic account of this transformation remains difficult, the comparative analysis with acoustic and semantic models for different tasks (behavior) and for different regions (fMRI) provides important insights into the interpretation of the unique contribution of DNNs to the predictions and on the nature of neural sound representations in STG.

The DNNs outperformed the other models for the sound dissimilarity task in the behavioral dataset and in nonprimary STG regions in the fMRI dataset. On the contrary, the semantic models outperformed the DNNs in the word dissimilarity task (behavior) (Fig. 3) and a spectrotemporal acoustic model matched the DNNs performance in HG (fMRI) (Fig. 4). This dissociation of results suggests that the additional contribution of the DNNs reflects a sound representation level that is neither acoustic (as reflected in HG responses) nor semantic (as reflected in the word task). We refer to this level as ‘intermediate.’ The variance-partitioning analysis corroborates this observation statistically, as it shows a significant unique contribution of DNNs in the sound dissimilarity task (behavior) and in STG regions (Figs. 3 and 4).

Our findings have relevant implications for current models of natural sound representation in nonprimary auditory cortex. A dominant view interprets fMRI response patterns to natural sounds in STG as evidence for a localist code implementing a one-to-one correspondence between highly selective neuronal populations and semantic categories^10,22^. The superiority of DNN models compared with categorical and other (nonauditory) continuous semantic representations suggests instead that auditory semantic information in STG is distributed spatially and is ‘componential,’ with neuronal populations encoding primitive components (dimensions) of multidimensional representations. Within this framework, sound categories may then be resolved in higher-level cortical areas (for example, ventro-lateral prefrontal cortex) through task/context dependent read-out of STG responses^36^ (Fig. 2). The layer-by-layer DNN analysis supports this hypothesis, showing that intermediate (rather than late) layers of the network architecture contribute maximally to the DNNs predictions (Fig. 5). In these intermediate layers, at the interface between the early (convolutional) blocks and the late (fully connected) blocks, complex features are formed and squeezed into lower-dimensional manifolds, after their initial expansion in early layers and before the task-specific refinement and categorical read-out occurring in the late (output) layers.

Distributed coding along a finite number of primitive dimensions accounts for flexible and adaptive representation of virtually infinite categories, as well as the within-category distinction of exemplars. On the contrary, a localist view requires specifying how many/which categories (and subcategories) STG actually encodes and requires ad hoc mechanisms for exemplar coding. It is conceivable that this proposed ‘general purpose’ coding mechanism coexists with specialized processing mechanisms, devoted, for example, to the linguistic analysis of speech^21,22^ (see below) and, possibly, to processing highly specific aspects of music^22,23^. In addition, fMRI investigations may highlight one or the other of these complementary neural coding depending on the chosen analytical approaches (multivariate versus univariate).

The MDS visualization of modeled sound representations and fMRI data (Fig. 2), as well as the pattern of results in PT and PP (intermediate between HG and STG, Supplementary Figs. 5–10) suggest a gradual transformation in the auditory cortex, with a progressive decrease of low-level acoustic features resolution and parallel enrichment of higher-level information. This is consistent with previous observations in the visual cortex^37^, with the notable difference that STG responses are best explained by intermediate DNN layers whereas the responses to natural images in higher-level visual cortex and in perceived image dissimilarity are best explained by late DNN layers^38^. This may reflect an important difference between visual and auditory cortex, although further investigations will be required to eliminate effects of architectural differences between sound and image classification networks.

Interestingly, the direct prediction of behavioral data from fMRI data revealed a significant unique contribution of HG and early auditory regions, together with STG. Judging the similarity of complex sounds requires actively attending to and comparing sounds’ acoustics^5^, which may be reflected in the responses of HG and early areas. These results predict an active role of early auditory areas, together with STG, in tasks requiring fine-grained sound identification.

At present, relating the primitive dimensions of STG representations to interpretable sound attributes requires further research. A suggestive hypothesis comes from theoretical and empirical research in auditory cognition, which conceives recognition of everyday sounds as inference about the sound-generating sources^1^. From this perspective, intermediate STG representations may reflect sound attributes that can be derived from the acoustic waveform and are functional to inferring sources from sounds, such as basic mechanisms of sound production, and the material and geometry of the objects. In future investigations, optimized stimulus sets and tasks are required to disentangle the representation of these dimensions from acoustics and general semantics. Combined with high spatiotemporal resolution methods (such as electrocorticography), these optimized designs may also help to relate our observations to actual neuronal computations^39^ and differentiating the contribution of the various STG regions to high-level representations of natural sounds. This differentiation was not resolved in our analyses, but has been reported in recent electrocorticography investigations of speech^40^.

All models considered here are general purpose auditory models that can be applied to any sound, including speech. With respect to speech sounds, however, these models account for their acoustic (as for all the other sounds) but not their linguistic processing. For speech, thus, intermediate representations, such as those emerging in the general purpose DNNs considered here, may encode combinations of features relevant to derive paralinguistic attributes (for example, gender, identity) but not linguistic units (for example, phonemes, syllables) required to extract the linguistic meaning conveyed by the speech waveform. Ample evidence indicates that intermediate linguistic units, such as phonemes, are represented in STG^39–44^ and that they are derived from the integration of several spectrotemporal cues^42^. Interestingly, recent studies indicate that articulatory mechanisms of sound production (for example, place, manner of articulation) provide relevant organizational dimensions for neural^40,42^ as well as behavioral^45^ responses to phonemes. Thus, our proposal that intermediate sound representations reflect basic mechanisms of sound production generalizes to all natural sounds the link between physical mechanisms, cortical organization and behavior, as previously observed for speech.

Besides the main comparison across model classes, our analyses enabled relevant considerations for each separate model classes as well. The comparison of acoustic models revealed that, whereas HG responses are better accounted for by the sound spectrotemporal modulation structure (MTF model), STG responses and perceived sound dissimilarity are better accounted for by an auditory dimensions model estimating perceived attributes of auditory sensation (pitch, loudness, brightness, periodicity and roughness). This is consistent with previous findings^6–8,46–48^ and confirms that STG and perceptual responses entail higher-order representations of the input sound. The similarity of results for MTF and DNN models in HG suggests that the biophysical validity of sound-to-event DNNs could be further improved by considering input acoustic representations other than the waveform or cochleagram-like representations. Also, understanding the relation between perceptual auditory dimensions and the representations emerging in the early/intermediate DNN layers may help link DNNs to psychoacoustics.

The set of models we considered included continuous models of semantics, obtained by neural network analyses of large natural language corpora. Importantly, these semantic models outperformed all other models in the word dissimilarity task and predicted a unique part of the perceived sound dissimilarity variance. This latter behavioral finding is, in our analyses, without a clear fMRI counterpart. Our fMRI dataset did not include (pre-)frontal cortex, potentially explaining why we did not find higher-order semantic representations in fMRI data. Future extensions should consider a larger brain coverage, including frontal as well as other regions of the semantic network^27^.

Finally, our selection of DNNs was limited to a convolutional architecture previously employed to predict fMRI responses^9^, and two closely related convolutional DNNs (VGGish and Yamnet) developed by Google^20^. The superior performance of these latter models is most probably due to the much larger and heterogeneous set of sounds and event categories used for training^34^. It will be interesting, in future work, to examine in detail how the set of training sounds, their semantic description and organization, and the task modulates the correspondence between DNNs and human behavioral and neural responses. Furthermore, it will be important to compare other neural network algorithms for automated sound recognition (including recurrent neural networks, long short-term memory networks and transformers) that exhibit ever improving performances. By releasing the data and code, this study contributes to an open-ended comparison framework that can set a benchmarking baseline for new comparisons and analyses of natural sound representation in behavior and in the brain.

## Methods

We considered data from two previously published studies on the behavioral and auditory cortical responses (fMRI) to natural sounds^8,30^. Here, we examined the extent to which these responses could be predicted by computational models of sound representation from three different classes: acoustic-processing models, semantic-processing models and DNN sound-to-event models.

### Behavioral and neuroimaging data

Behavioral data from Giordano et al.^30^ (Experiment 2) were collected while two groups of different participants (*N*_p_ = 40, 20 per group; 26 females, 14 males; median age, 25 years) carried out either a task estimating the dissimilarity of natural sounds (sound dissimilarity condition; *N*_s_ = 80), or a task estimating the dissimilarity of verb plus noun sentences describing the sound source for each of the 80 sounds (for example, ‘crying baby’; word dissimilarity condition; random assignment of participants to the experimental conditions). Participants to each condition evaluated the dissimilarity of two separate sets of 40 stimuli (living object and nonliving object sets; median sound duration, 5.1 s) on separate sessions (stimulus set order counterbalanced across participants), using hierarchical sorting^35^. In particular, for each sound set, they initially created 15 groups of similar sounds (sound dissimilarity condition) or of verbal sound descriptors (word dissimilarity condition) by grouping onscreen icons (random assignment of sounds/words to onscreen icons) activating the stimuli when clicked upon, and merged at each subsequent step the two most similar groups of stimuli until all stimuli were merged in the same group. Pairwise stimulus dissimilarity was defined as the step at which two stimuli were merged in the same group. No statistical methods were used to predetermine sample sizes, but our sample sizes are similar to those reported in previous publications^33,35^.

High-field 7 T fMRI data from Santoro et al.^8^ (Experiment 2) were collected while participants (*N*_p_ = 5; three females, two males; median age, 27 years) listened attentively to natural sound stimuli (*N*_s_ = 288) from six categories (human nonspeech vocal sounds, speech sounds, animal cries, musical instruments, scenes from nature, tool sounds; 48 sounds per category). The 288 stimuli were divided into four nonoverlapping sets of 72 sounds each (12 sounds per category in each set; sound duration, 1 s). Participants underwent two subsequent fMRI sessions (six runs, with three repeated presentations of each of two of the 72-sound sets, each on a separate run; randomized presentation order of sounds within each run). No statistical methods were used to predetermine sample sizes, but our sample sizes are similar to those reported in previous publications^7,27^. fMRI data were acquired during an event-related design (repetition time (TR) = 2,600 ms, acquisition time (TA) = 1,200 ms, echo time (TE) = 19 ms, generalized autocalibrating partially parallel acquisition (GRAPPA) = 2, partial Fourier = 6/8, flip angle = 70°, voxel size = 1.5 mm^3^, *N* slices = 46 with no gap between slices) with an acquisition volume covering the brain transversally from the inferior portion of the anterior temporal pole to the superior portion of the STG, bilaterally. Stimuli were presented during a silent gap (TR minus TA) of 1.4 s between subsequent volume acquisitions (interstimulus intervals chosen randomly between 2, 3 and 4 TRs (5.2, 7.8 and 10.4 s) with sound onset occurring at random either 50, or 200 or 350 ms after MRI volume acquisition). Participants performed an incidental one-back repetition detection task (6.49% of all sound trials) and responded with a button press when a sound was repeated (fMRI data for one-back trials not considered because of motor contamination and stimulus-habituation effects). Estimates of fMRI responses for each stimulus and participant were computed using the two-step procedure of Kay et al.^49^. Specifically, in a first step we estimated voxel-specific hemodynamic response functions (HRFs) in each of the four cross-validation (CV) folds (using finite impulse response modeling and all training-set stimuli). In a second step, using these voxel-specific HRFs and one predictor per sound we obtained the fMRI response estimates as the weights of a general linear model fit to the fMRI time series. This was done separately for the training and test-set (see Santoro et al.^7,8^; one-back trials not considered). For each participant, we considered response estimates from six bilateral masks defined anatomically to include HG, planum temporale (PT), planum polare (PP) and the pSTG, mSTG or aSTG.

The behavioral experiment was approved by the McGill Research Ethics Board. The fMRI experiment was approved by the Ethical Committee of the Faculty of Psychology and Neuroscience of Maastricht University. Procedures in both experiments followed the principles expressed in the Declaration of Helsinki. Informed consent was obtained from all experiment participants. Participants in the behavioral experiments were compensated at an hourly rate of ten Canadian dollars per hour and in the fMRI experiments with gift certificates with a value of 7.5 euros per hour. Data collection and analysis were not performed blind to the conditions of the experiments.

### Computational models

We considered sound representation models from three classes: acoustic processing models, models of the semantic structure of the sound events (category structure and natural language processing of verbal descriptors of the sound source) and supervised sound-to-event DNNs trained to learn sound event categories from sounds. Models could include a different number of component representations, measuring a qualitatively different transformation of the input stimulus. For the fMRI sound stimuli, acoustic and DNN models were estimated considering the entire stimulus length (1 s). For the sound stimuli in the behavioral experiment, they were estimated considering the first 2 s of the waveform.

#### Acoustic processing models

We considered five different acoustic-processing models implementing biophysically and/or psychophysically informed signal transformations.

##### Cochleagram

We used the NSLtools implementation of the cochleagram representation^13^, modeling the dynamic spectral analysis at the periphery of the auditory system (outer and inner ear, and cochlear filtering^11^). The cochleagram representation consisted in a time-varying signal (temporal resolution = 8 ms, *N* time samples = 1,999 and 999 for the behavioral and fMRI experiment stimuli, respectively) output from each of 128 cochlear filters with log_2_ spaced frequencies (179–7,246 Hz). The cochleagram model included two components, the time-varying cochleagram (32,000 parameters for the behavioral dataset and 16,000 parameters for the fMRI dataset) and the time-averaged cochleagram (128 parameters for both datasets).

##### Stabilized auditory image

We considered the AIM-MAT v.1.5 implementation of the stabilized auditory image model^12^. SAI (one model component) implements a periodicity analysis (roughly akin to an autocorrelation) of the neural activity pattern simulated at the level of the subcortical auditory system^12^. More specifically, the SAI is a time-varying representation (temporal resolution = 5 ms, *N* time samples = 401 and 201 for the behavioral and fMRI experiment stimuli, respectively) of the short-term signal periodicity (lags from 0 to 35 ms at sound sampling rate resolution) in different cochlear channels (linearly spaced on an equivalent rectangular bandwidth (ERB)-rate scale between 100 and 6,000 Hz). The SAI model included two components, the time-varying SAI (33,768,000 parameters for the behavioral dataset and 16,968,000 parameters for the fMRI dataset) and the time-averaged SAI (84,000 parameters for both datasets).

##### Modulation transfer function

We used the NSLtools implementation of the multiresolution spectrotemporal model of Chi et al.^13^. The MTF quantifies the spectrotemporal modulations (scale from 0.25 to 8 cycles/octave; unsigned rate from 4 to 156 Hz) in each channel of the input cochleagram representation (128 channels on; 179–7,246 Hz) as a function of time (temporal resolution = 8 ms). The MTF representation produced complex numbers in output. The MTF model comprised six components, three for the time-varying MTF (MTF magnitude, MTF phase and MTF magnitude and phase together, used to generate a between-stimulus distance in the complex plane, see below) and three for the time-averaged MTF (magnitude, phase and phase plus magnitude; total parameters for MTF model across these six components = 19,790,848 and 9,934,848 for behavioral and fMRI dataset, respectively; minimum number of parameters = 19,712 for both datasets; maximum number of parameters = 9,856,000 and 4,928,000 for the behavioral and fMRI dataset, respectively).

##### Texture model

We used the Sound Texture Synthesis Toolbox v.1.7 of the sound texture analysis model by McDermott and Simoncelli^31^. The texture model (best-performing set of statistics for identification experiments in fig. 5a in McDermott and Simoncelli^31^) includes five separate components measuring different summary statistics of the time-varying amplitude envelopes of the cochleagram (32 frequency bands evenly spaced on an ERB-rate scale from 20 Hz to 10 kHz), or of the modulation analysis specific to each of the frequency bands (six modulation filters with center frequencies log-spaced between 30 and 100 Hz): (1) the marginal statistics of the band-specific envelopes (mean, variance, skewness and kurtosis); (2) the pairwise correlations C between band-specific amplitude envelopes; (3) the power in each modulation band; (4) the pairwise correlation between the modulation analysis of each frequency band at a constant modulation filter frequency (C1 correlations); (5) the correlation between the modulation analysis of the same frequency band at adjacent modulation filter frequencies (C2 correlations). For both the behavioral and fMRI datasets, the texture model included a total of 6,656 parameters across the five components for both datasets (minimum = 32; maximum = 6,144).

##### Auditory dimensions

We considered a model quantifying the temporal profile and summary statistics of five time-varying measures of auditory sensation (temporal resolution = 1 ms, *N* time samples of all time-varying auditory dimensions = 1,999 and 999 for the behavioral and fMRI experiment stimuli, respectively): pitch^14^, loudness^15^, periodicity^32^, timbre brightness^33^ and roughness^16^. Time-varying loudness and brightness were derived from the instantaneous specific loudness of the input signal^15^, as estimated in the Genesis Loudness Toolbox. Instantaneous specific loudness measures the time-varying contribution to the overall instantaneous loudness (temporal resolution = 1 ms) in separate frequency bands (*N* frequency bands = 153, equally spaced on an ERB-rate scale between 20 and 15,000 Hz). For each temporal frame, loudness (measured on a sone scale) was then defined as the sum of the instantaneous specific loudness across frequency bands, whereas timbre brightness was defined as the spectral centroid, that is, as the specific loudness weighted average ERB-rate frequency^33^. Time-varying pitch (measured on an ERB-rate scale) and periodicity (ratio of periodic to aperiodic power, in dB) were estimated using the Yin model by Cheveigné and Kawahara^14^. Time-varying roughness^16^ was finally estimated using the model implemented in the MIRtoolbox v.1.7.2. The auditory dimensions model included three components for each of the five auditory dimensions (time-varying dimension and first two moments: mean and s.d. of the time-varying dimension), for a total of 15 components. The auditory dimensions model included 50,005 and 10,005 parameters for the behavioral and fMRI dataset, respectively (minimum = 1 for both datasets; maximum = 1,999 and 999 for the behavioral and fMRI dataset, respectively).

#### Semantic models

We considered two classes of models quantifying the semantics of natural sounds in terms of attributes of the sound source. For the fMRI dataset only, we considered a six-component category model that differentiated each of the six sound categories (speech, human voices, animal vocalizations, music, nature sounds and tool sounds; one parameter for each of the six components) from the rest of the sounds (for example, the speech component was a between-stimulus distance defined as zero for between-speech distances and one otherwise; see refs. ^22–24^ for category sensitivities in the auditory brain). For both datasets, we also considered three NLP models (one model component each) measuring the embedding of semantic data for the sound stimuli (verb plus noun sentences identifying the sound source). For the behavioral datasets, name plus verb sound descriptors were derived from the results of a preliminary verbal identification experiment in Giordano et al.^30^, Experiment 1) during which 20 individuals, who did not take part in Experiment 2, were asked to identify the sound-generating events using one verb and one or two nouns. In particular, for each of the sound stimuli the name plus verb sound descriptors considered for the analyses in this study, and evaluated by participants in the word condition, were the modal verbs and nouns (that is the most frequent verbs and nouns) across the 20 participants to the verbal identification experiment in Giordano et al.^30^, Experiment 1). For the fMRI dataset, labels were instead defined by the experimenter to be as close as possible to the verbal descriptors of the AudioSet taxonomy^34^. We estimated the semantic embeddings for the verb plus noun sentences by considering three models: GloVe^18^ (unsupervised learning of corpora statistics; *n* parameters = 300), a word2vec trained on the Google News dataset: GNewsW2V^17^ (*n* parameters = 300) and the Google Universal Sentence Encoder: GUSE^19^ (*n* parameters = 512). For GloVe and GNewsW2V, we estimated one semantic embedding for each sound stimulus by averaging across the semantic embeddings for the name and verb sound descriptors. With GUSE, we instead directly estimated one semantic embedding for the name plus verb sentence. Each of the GloVe, GNewsW2V and GUSE models included one single model component.

#### Sound-to-event DNNs

We considered three pretrained feed-forward convolutional networks trained to learn sound event categories from an input acoustic representation: VGGish and Yamnet by the Google Research team^20,34^, and the dual-task network by Kell et al.^9^. Both VGGish and Yamnet (input audio to both trained networks = 0.975 s, converted to a stabilized log-mel spectrogram) were trained to classify 10 s YouTube audio tracks with a set of 527 (VGGish) or 521 (Yamnet) labels from the Audioset ontology^34^. Kell’s dual-task network (input audio to trained network = 2 s converted to a cochleagram representation) carried out two classification tasks on the input 2 s audio excerpts: a 587-way word recognition task, and a 41-way music genre recognition task. VGGish consists of an input layer, four convolutional blocks (convolution, ReLU, MaxPooling), followed by three fully connected blocks (fully connected, ReLU) that progressively implement a dimension reduction to a 128-dimensional semantic embedding (output layer). Yamnet consists of an input layer (the same as VGGish), a standard convolutional block (convolution, batch normalization, ReLU) followed by a series of 13 depthwise separable convolution blocks, each including six layers (depthwise conv2d, batch normalization, ReLU, conv1d, batch normalization, ReLU). This generates a three by two array of activations for 1,024 kernels, which are then averaged to give a 1,024-dimension embedding and put through a single logistic layer to get the 521 per-label output scores. Kell’s network consists of an input layer, followed by two convolutional blocks (convolution, normalization pooling), followed by one convolutional block connected to the two task-specific branches (two convolutions, pooling, fully connected layer and a final softmax layer for class-probability prediction). For each DNN, we selected as model components the activations in the last pooling layer of each convolutional block, as well as in each of the remaining noninput layers not part of a convolutional block (7, 14 and 9 model components for VGGish, Yamnet and Kell’s network, respectively). For the behavioral experiment stimuli, layer activations in the VGGish and Yamnet model were estimated for each of two subsequent 0.975 s windows and then averaged across windows. For both the behavioral and fMRI datasets, the Kell model included a total of 557,152 parameters (minimum = 1,024; maximum = 177,504), the VGGish model included a total of 192,640 parameters (minimum = 128; maximum = 98,304), and the Yamnet model included a total of 380,928 parameters (minimum = 6,144; maximum = 98,304). For VGGish only, we also considered a random-weights control VGGish model with Kaiming He weights initialization.

### Model representation analyses

We assessed the representation of computational models in behavioral and fMRI data using a cross-validated framework predicting group-averaged behavioral and fMRI between-stimulus distances from model-based distances (Supplementary Fig. 1). Behavioral data for each participant were already distance matrices of perceived between-stimulus dissimilarities (*N* stimulus pairs in the distance matrices = 780 for each of the two stimulus sets). For each participant in the fMRI experiment, we estimated ROI-specific between-stimulus distances as the Euclidean distance between the demeaned generalized linear model betas for the different stimuli within a given ROI^28^ (*N* stimulus pairs in the distance matrices = 23,220 for the training-set data and 2,556 for the test-set data, see below). For each computational model (except the category model, already defined as a distance, see above), we created a separate between-stimulus cosine distance for each of the model components (see, for example, Mikolov et al.^17^ for cosine similarity in NLP and audio DNNs). For sound stimuli in the fMRI experiment (duration = 1 s), VGGish and Yamnet representations were computed by considering the first 0.960 and 0.975 s of the sound signals, respectively (one DNN analysis window; zero padding of sound stimuli to fill one analysis window for the Kell network). For sound stimuli in the behavioral experiment (median duration = 5.1 s), we estimated acoustic representations and representations in the Kell network by considering the first 2 s of the sound stimuli (one analysis window in the Kell network; zero padding for stimuli shorter than 2 s). For the same stimuli, we estimated a time-varying VGGish and Yamnet representation by considering two subsequent analysis windows of 0.960 and 0.975 s, respectively. Note that the computation of the model component distances did not include an optimization of the model parameters, that is, all parameters were given equal weight for the component distance computation.

For both behavioral and fMRI data, we adopted a cross-validated linear regression framework predicting behavioral and fMRI group-averaged distances from sets of model component distances (for example, prediction of behavioral dissimilarities from all components of acoustic models; CV across participants for behavioral data and across both participants and stimuli for fMRI data). We considered a framework predicting group-averaged distances because of previous studies showing reduced reliability and acoustic-model assessment at the level of the single-participant estimating dissimilarities with a behavioral hierarchical sorting task^35^. For both datasets, significance testing relied on a permutation-based framework (Mantel’s test, relying on the permutation of rows and columns of the distance matrix) with maximum-statistics correction for multiple comparisons (MC^50^, one-sided inference for all tests exception done for the contrasts which relied on two-sided inference). Note that our permutation framework does not make any assumption about the distributional properties of the data. We carried out six subsequent analyses. First, we assessed whether all the model components together, or all the acoustic-model components, or all the semantic model components or all the DNN model components predicted a significant portion of the behavioral or fMRI distances, and carried out pairwise predictive variance contrasts between the acoustics, semantics and DNN model classes (MC correction across mode classes or contrasts, and across fMRI ROIs). Second, we assessed the significance of the predictive variance for each of the models within each model class, and carried out pairwise contrasts between same-class models (MC correction across same-class models or contrasts, and across fMRI ROIs). Third, we measured the unique predictive variance for each of the model classes, and carried out pairwise contrasts between unique predictive variances for the different model classes (see below for details on partitioning of cross-validated variances). Fourth, we measured the predictive variance components common to the different model classes (three two-model common variances, and one three-model common variance; MC correction across common variance components, and across fMRI ROIs). Fifth, we quantified in detail the predictive power of each DNN by carrying out an analysis of the predictive power of each layer (layer-by-layer analysis), or by each layer together with the preceding network layers (layer-cumulative analysis). Finally, we carried out an analysis generalizing model representations from fMRI to behavioral data, that is, predicting behavioral dissimilarities from fMRI mapped, by linear regression, onto the space of computational model distances.

We used two different CV schemes for the behavioral and fMRI data. The behavioral data were collected on two separate sound sets (living and nonliving sound-generating object), explicitly selected to have a different semantic structure (and potentially correlated acoustical structure). The fMRI data were instead collected for four separate stimulus sets characterized by the same semantic-category structure and more homogeneous acoustical structure. Because of these design properties, we cross-validated across stimulus sets only for the fMRI analyses. For both datasets, we used a repeated split-half approach for cross-validating predictive variance estimates (*R*^2^_CV_) across groups of participants (behavioral data: ten participants for training and ten for testing; fMRI data: three participants for training and two for testing; 100 random splits considered for the behavioral data; all the ten possible splits of three and two participants considered for the fMRI data). For each split, we averaged behavioral or fMRI data across training and test participants separately, and estimated the betas of a standardized linear regression model predicting test-set data from the model component distances (independent *z*-scoring of test and training-set data and predictors). The predictive variance *R*^2^_CV_ was estimated as 1 – SSE_test_/SST_test_, where SSE_test_ = sum of the squared error of the prediction of the test-set data from the training-set regression betas applied to the test-set predictors and SST_test_ = total sum of squares of the test-set data. For each split, we also computed 10,000 row per column permutations (same object permutations kept across splits). We carried out inference on the median of the *R*^2^_CV_ measures across splits using a maximum-statistics approach for the correction of MC at the FWER = 0.05 level. For each CV split, we also computed a cross-validated measure of noise ceiling capturing the maximum predictable variance in the behavior and fMRI group-averaged distances. Noise ceiling was estimated as 1 – SSD_test-train_/SST_test_ where SSD_test-train_ = sum of the squared differences between test and training behavioral or fMRI group-averaged distances. The noise ceiling estimate is thus distributional rather than pointwise. Thus defined, the noise ceiling can give an indication on whether future studies should rely on better models (when none approach the noise ceiling) or, when we reach the noise ceiling, on better data, characterized by a lower noise level and/or acquired through a more complex design that is capable of challenging the best predictive models. Unique and common predictive variances were estimated by applying the commonality analysis approach^29^ to predictive *R*^2^_CV_ measures. In particular, for the analysis partitioning the predictive variance for the acoustic, semantics and DNN model classes, predictive variance components were estimated by applying the following equations to the *R*^2^_CV_ values for each model class alone, for each pair of model classes and for the three model classes together:

1 $$U_i = R^2_{{\mathrm{CV.}}ijk}-R^2_{{\mathrm{CV.}}jk}$$

2 $$C_{{ij}} = R^2_{{\mathrm{CV.}}{ik}} + R^2_{{\mathrm{CV.}}{jk}}-R^2_{{\mathrm{CV.}}{k}} - R^2_{{\mathrm{CV.}}{ijk}}$$

3 $$C_{{ijk}} = R^2_{{\mathrm{CV.}}{i}} + R^2_{{\mathrm{CV.}}{j}} + R^2_{{\mathrm{CV.}}{k}}-R^2_{{\mathrm{CV.}}{ij}}-R^2_{{\mathrm{CV.}}{ik}}-R^2_{{\mathrm{CV.}}{jk}} + R^2_{{\mathrm{CV.}}{ijk}}$$

where: *U*_*i*_ is the unique predictive variance for model *i* (for example, all semantic models together); *C*_*i*_ is the common predictive variance for models *i* and *j*, and *C*_*ijk*_ is the common predictive variance for the three models *i*, *j* and *k*. The analysis predicting behavioral dissimilarities from fMRI data was divided in three steps. First, for each ROI and CV partition (same training partitions considered for the above analyses), we estimated the training-step standardized regression betas of the model predicting fMRI distances from DNN model distances (components from all DNNs together), and applied the regression betas to the standardized DNN model distances for each of the two stimulus sets in the behavioral experiment. These model-based behavioral dissimilarity predictions were then averaged across fMRI CV folds to yield one fMRI-based behavior prediction for each of the fMRI ROIs. These DNN-based fMRI models of the behavioral stimuli were finally considered as predictors within the same cross-validated permutation-based framework adopted to assess the representation of the DNN models in behavioral data. In particular, we evaluated the behavior-predictive power of all fMRI ROIs together, and of each fMRI ROI in isolation, and adopted the commonality analysis framework to separate the unique and common behavior variance predicted by two key fMRI ROIs, HG and pSTG. From the statistical point of view, this analysis constitutes a stringent external validation test of the representation of DNNs in largely different fMRI and behavioral datasets. From the conceptual point of view, this approach measures the overlap of DNN representations in fMRI and behavioral data because the prediction would not be possible if fMRI and behavior were driven by separate components of the DNN variance.

### Reporting summary

Further information on research design is available in the Nature Portfolio Reporting Summary linked to this article.

## Online content

Any methods, additional references, Nature Portfolio reporting summaries, source data, extended data, supplementary information, acknowledgements, peer review information; details of author contributions and competing interests; and statements of data and code availability are available at 10.1038/s41593-023-01285-9.

## Supplementary information

Supplementary Information Supplementary Figs. 1–12 and Tables 1–16.

Reporting Summary

## Data Availability

All data used in the analyses are available at the following Dryad repository: 10.5061/dryad.0p2ngf258

## Code availability

All code used for the analyses is available at the following Dryad repository: 10.5061/dryad.0p2ngf258
