# Supplementary material for: Intermediate acoustic-to-semantic representations link behavioral and neural responses to natural sounds
Source: Nat Neurosci. 2023 Mar 16;26(4):664–72. doi: 10.1038/s41593-023-01285-9 (PMC10076214; doi:10.1038/s41593-023-01285-9)
Supplement: Supplementary file 1 — Supplementary Figs. 1–12 and Tables 1–16. [file 41593_2023_1285_MOESM1_ESM.pdf]

nature neuroscience

Article

https://doi.org/10.1038/s41593-023-01285-9

Intermediate acoustic-to-semantic representations link behavioral and neural responses to natural sounds

In the format provided by the
authors and unedited

Supplementary Information for

Intermediate acoustic-to-semantic representations link behavioural and neural responses to natural sounds

Bruno L. Giordano, Michele Esposito, Giancarlo Valente, Elia Formisano
Correspondence: bruno.giordano@univ-amu.fr ;
e.formisano@maastrichtuniversity.nl

This PDF file includes:

Supplementary Figures 1 to 12
Supplementary Tables 1 to 16

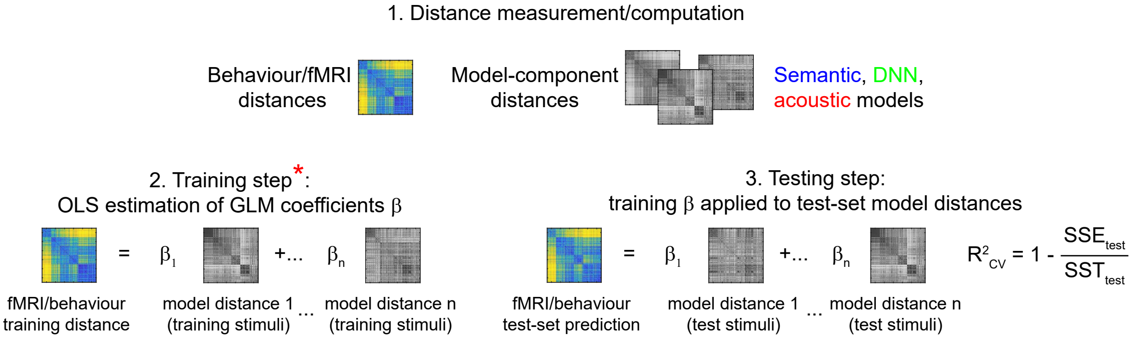

Supplementary Fig. 1: **Data-analysis framework.** We analyse the model-based prediction of fMRI and behavioural data with a distance-based approach extending representational similarity analysis (RSA) within a cross-validated variance partitioning framework. The β coefficients of a general linear model (GLM) predicting training-set behaviour/fMRI distances are applied to the test-set model distances. The generalization of the training-step β coefficients to the test data yields a prediction for the test-set behaviour/fMRI distances, whose departure from the observed test-set behaviour/fMRI distances is measured with the cross-validated R-squared (R^2_{CV}) statistic. The red asterisk marks the step involving a weight/parameter optimization (training step). No weight or parameter was optimized to measure/compute the analysed distances, or during the testing step. OLS = ordinary least squares; SSE = sum of squared errors; SST = total sum of squares.

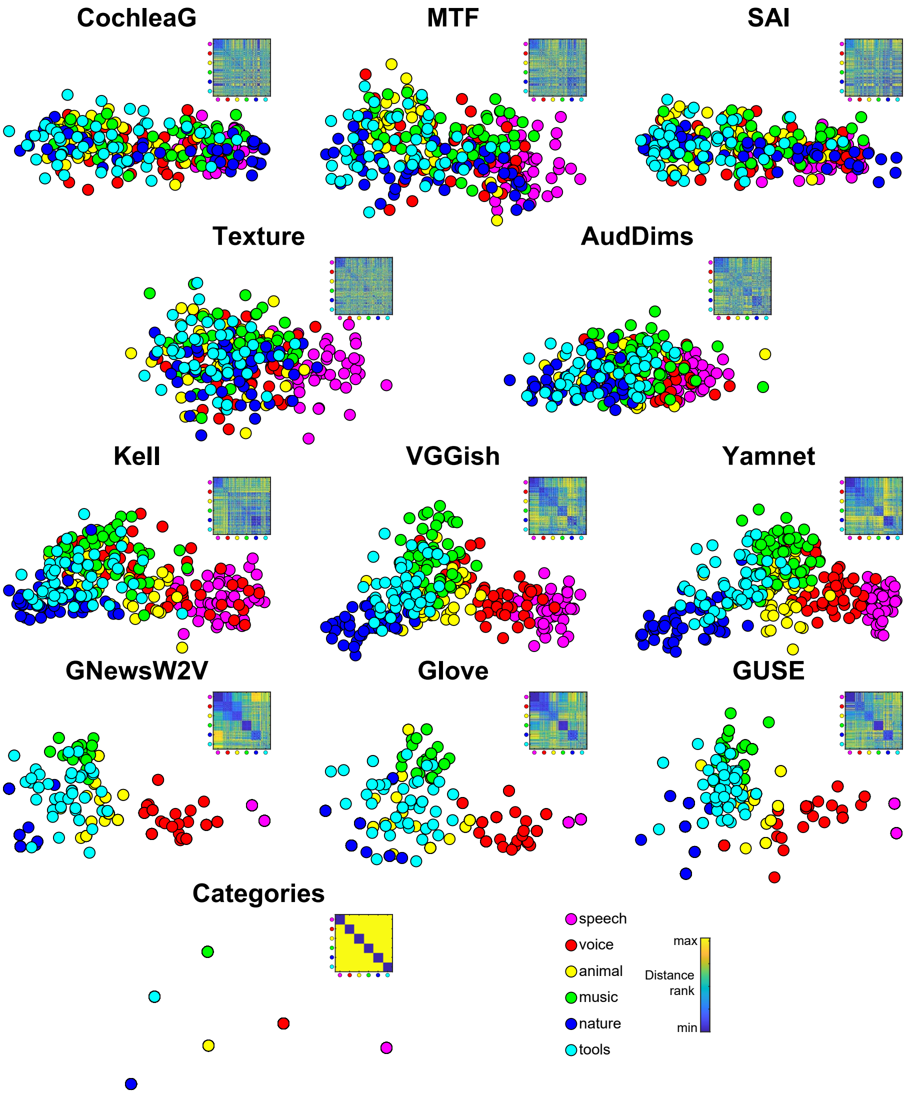

Supplementary Fig. 2: **Visualizing acoustic, semantic and DNN models of natural sound representation.** Metric multidimensional scaling (MDS) performed on the standardized distance averaged across all model components (e.g., all layer-specific distances for the Yamnet network). All MDS solutions were Procrustes-rotated to the metric MDS fit to the average pSTG distance (N dimensions considered = 60; only translation and rotation considered). For each MDS solution, we also show the ranked dissimilarity matrix.

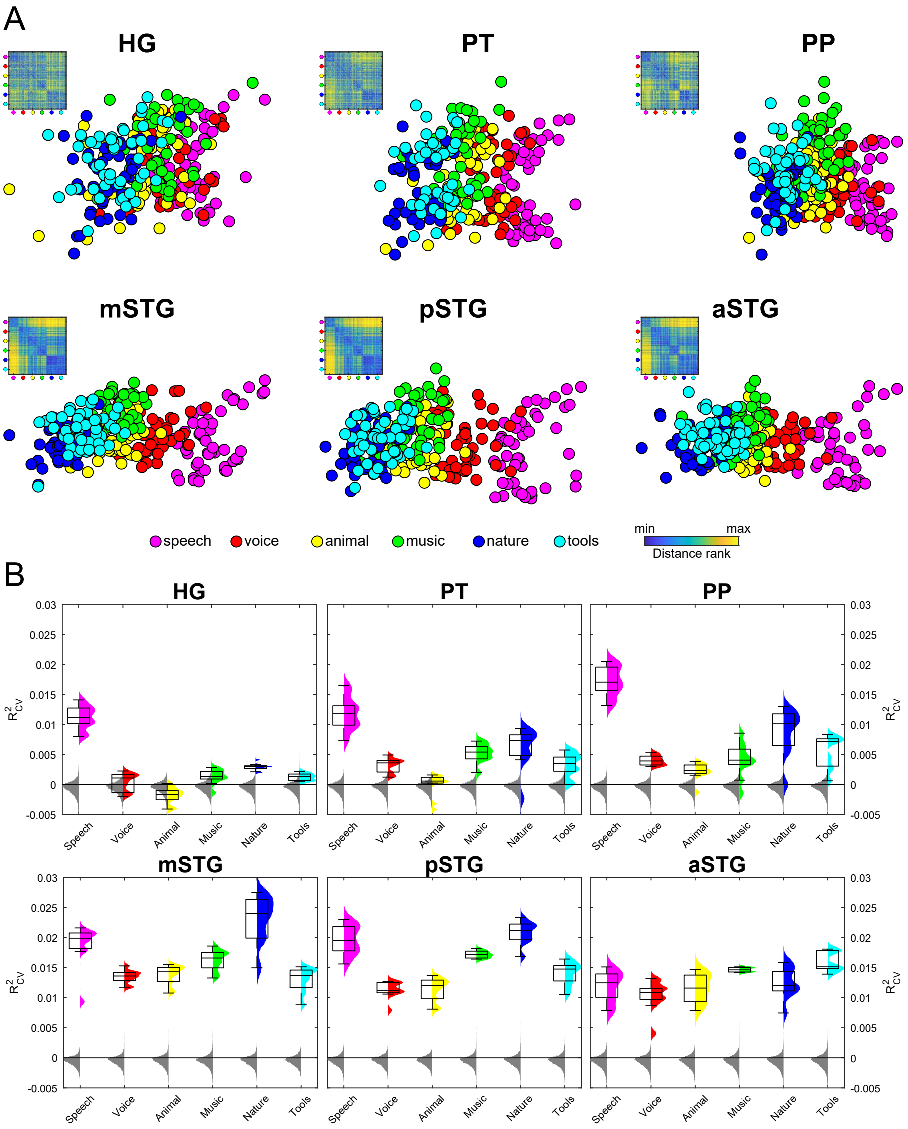

Supplementary Fig. 3: **Visualizing acoustic-to-semantic representations in the brain. A.** Metric multidimensional scaling (MDS) of training-set fMRI distances averaged across cross-validation folds and participants (HG = Heschl's gyrus; PT = planum temporale; PP = planum polare; m/p/aSTG = middle/posterior/anterior superior temporal gyrus). The MDS representations for each ROI were Procrustes rotated to the metric MDS of between-stimulus distances in pSTG. For each MDS solution, we also show the ranked dissimilarity matrix. **B.** R^2_{CV} for each of the components of the category model. Colours = across-CV distributions, with corresponding box plot (centre = median; lower/upper box limits = 1st/3rd quartile). Dark grey = permutation distribution of the median of the training-set R^2_{CV} across CV folds. Larger R^2_{CV} values denote greater similarity of the category exemplars relative to the cloud of the rest of the sound stimuli within the representational space. See Supplementary Table 16, for numerical results. N fMRI participants = 5.

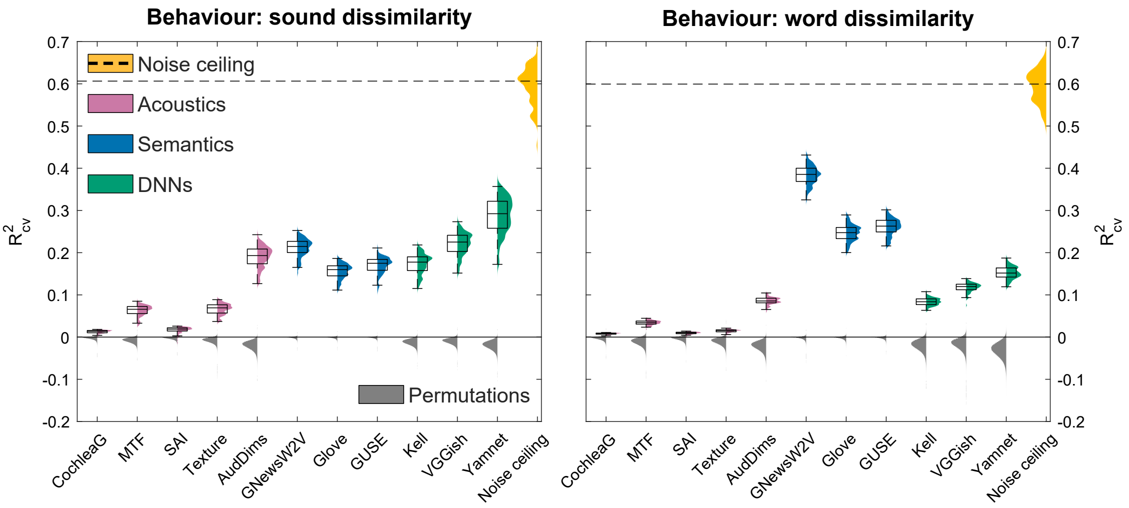

Supplementary Fig. 4: **Acoustic and semantic representations in behaviour: model-by-model analysis.** Coloured distributions = plugin distribution of R^2_{CV} across CV folds (and corresponding box-plot : centre = median; lower/upper box limits = 1st/3rd quartile; bottom/-top whisker = data within 1.5 interquartile ranges from 1st and 3rd quartiles, respectively); dark grey = cross-CV fold median of the permutation results; orange = noise ceiling (dashed line = median noise-ceiling across CV folds. N sound or word dissimilarity participants = 20.

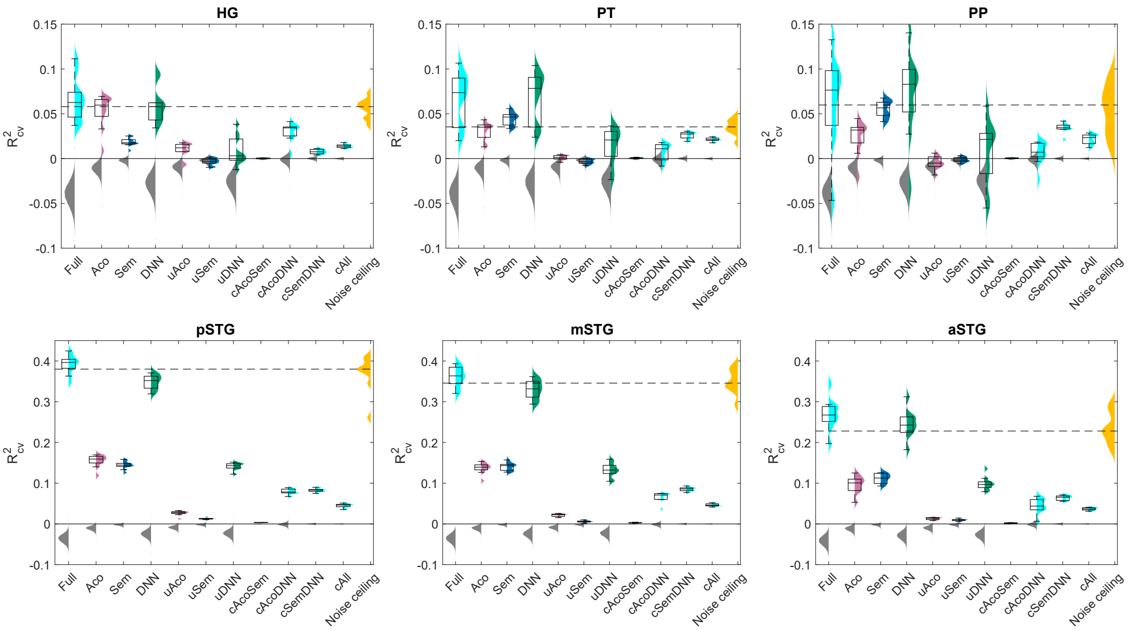

Supplementary Fig. 5: **Acoustic and semantic representations in all fMRI ROIs: variance partitioning analysis, speech stimuli included.** Full = all models together; Aco = acoustic models; Sem = semantic models; DNN = sound-to-event deep neural networks; u = unique predictive variance component; c = common predictive variance component; cAll = predictive variance component common to the acoustic, semantic, and DNN models). Coloured distributions = plugin distribution of R^2_{CV} across CV folds (and corresponding box-plot : centre = median; lower/upper box limits = 1st/3rd quartile; bottom/top whisker = data within 1.5 interquartile ranges from 1st and 3rd quartiles, respectively); dark grey = cross-CV fold median of the permutation results; orange = noise ceiling (dashed line = median noise-ceiling across CV folds. HG = Heschl's gyrus; PT = planum temporale' PP = planum polare; p/m/aSTG = posterior, mid or anterior portion of the superior temporal gyrus. N fMRI participants = 5.

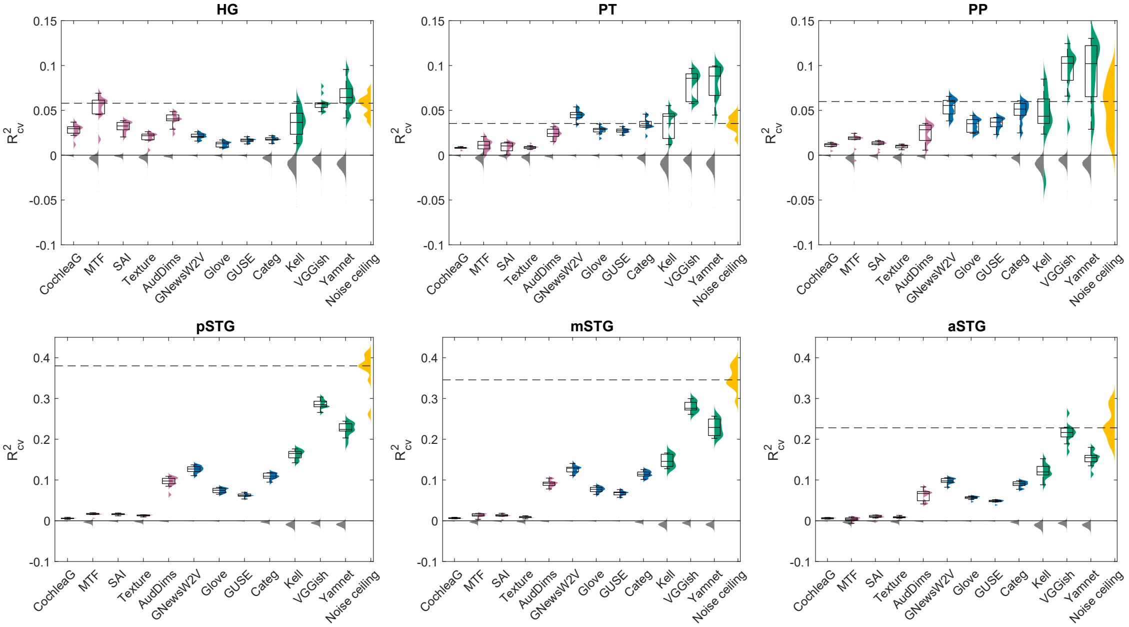

Supplementary Fig. 6: **Acoustic and semantic representations in all fMRI ROIs: model-by-model analysis, speech stimuli included.** Coloured distributions = plugin distribution of R^2_{CV} across CV folds (and corresponding box-plot : centre = median; lower/upper box limits = 1st/3rd quartile; bottom/top whisker = data within 1.5 interquartile ranges from 1st and 3rd quartiles, respectively); dark grey = cross-CV fold median of the permutation results; orange = noise ceiling (dashed line = median noise-ceiling across CV folds. HG = Heschl's gyrus; PT = planum temporale' PP = planum polare; p/m/aSTG = posterior, mid or anterior portion of the superior temporal gyrus. N fMRI participants = 5.

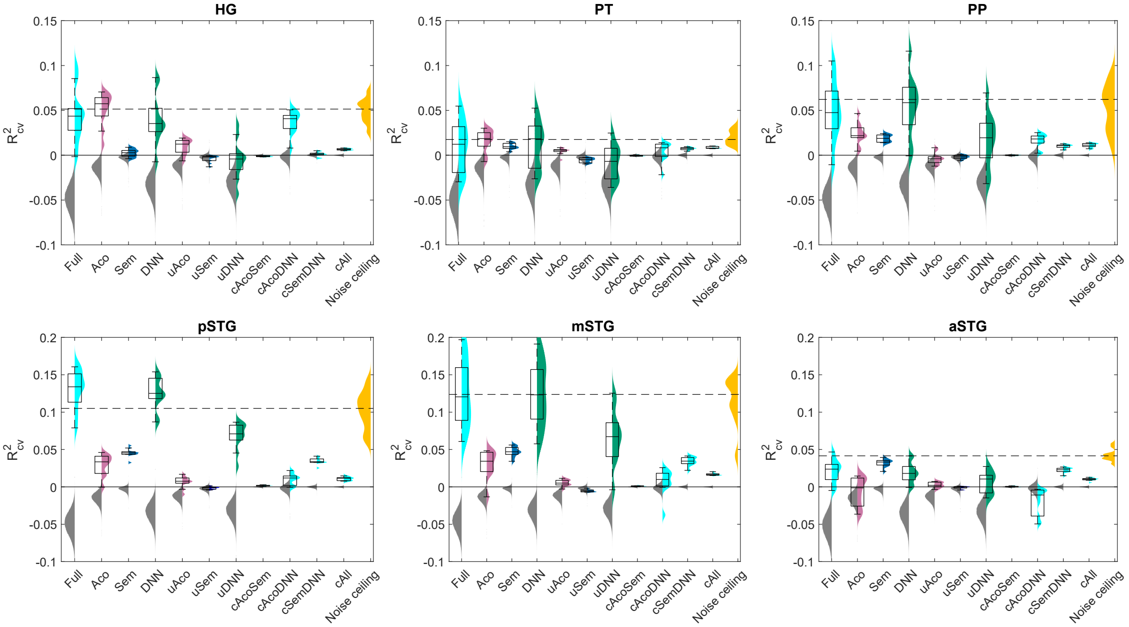

Supplementary Fig. 7: **Acoustic and semantic representations in all fMRI ROIs: variance partitioning analysis, speech stimuli excluded.** Full = all models together; Aco = acoustic models; Sem = semantic models; DNN = sound-to-event deep neural networks; u = unique predictive variance component; c = common predictive variance component; cAll = predictive variance component common to the acoustic, semantic, and DNN models). Coloured distributions = plugin distribution of R^2_{CV} across CV folds (and corresponding box-plot : centre = median; lower/upper box limits = 1st/3rd quartile; bottom/top whisker = data within 1.5 interquartile ranges from 1st and 3rd quartiles, respectively); dark grey = cross-CV fold median of the permutation results; orange = noise ceiling (dashed line = median noise-ceiling across CV folds. HG = Heschl's gyrus; PT = planum temporale' PP = planum polare; p/m/aSTG = posterior, mid or anterior portion of the superior temporal gyrus. N fMRI participants = 5.

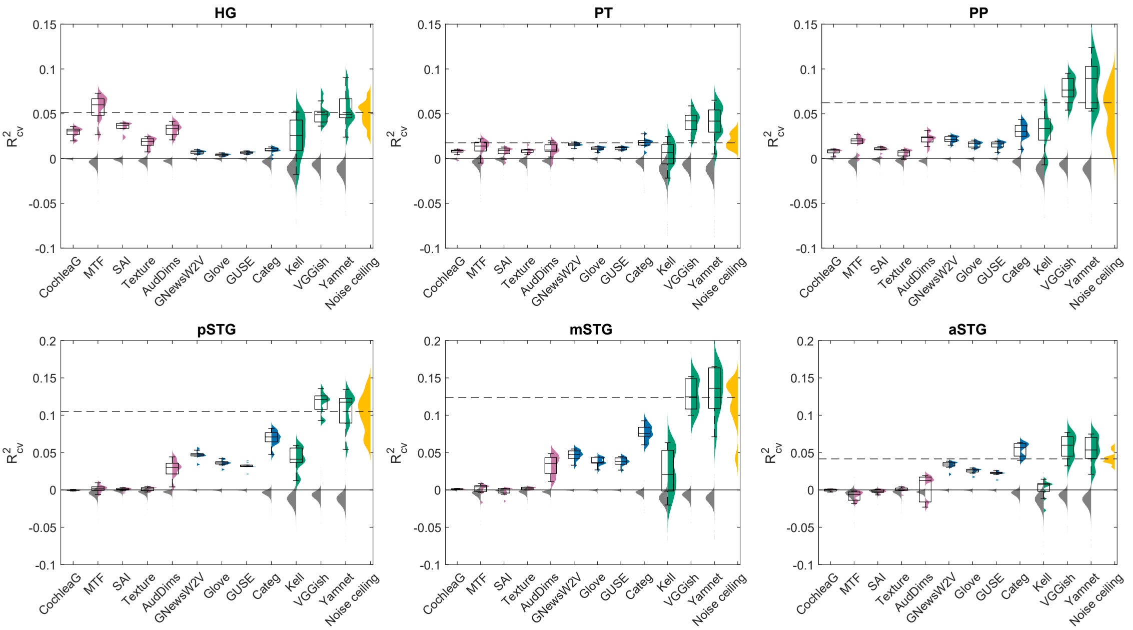

Supplementary Fig. 8: Acoustic and semantic representations in all fMRI ROIs: model-by-model analysis, speech stimuli excluded. Coloured distributions = plugin distribution of R^2_{CV} across CV folds (and corresponding box-plot : centre = median; lower/upper box limits = 1st/3rd quartile; bottom/top whisker = data within 1.5 interquartile ranges from 1st and 3rd quartiles, respectively); dark grey = cross-CV fold median of the permutation results; orange = noise ceiling (dashed line = median noise-ceiling across CV folds. HG = Heschl's gyrus; PT = planum temporale' PP = planum polare; p/m/aSTG = posterior, mid or anterior portion of the superior temporal gyrus. N fMRI participants = 5.

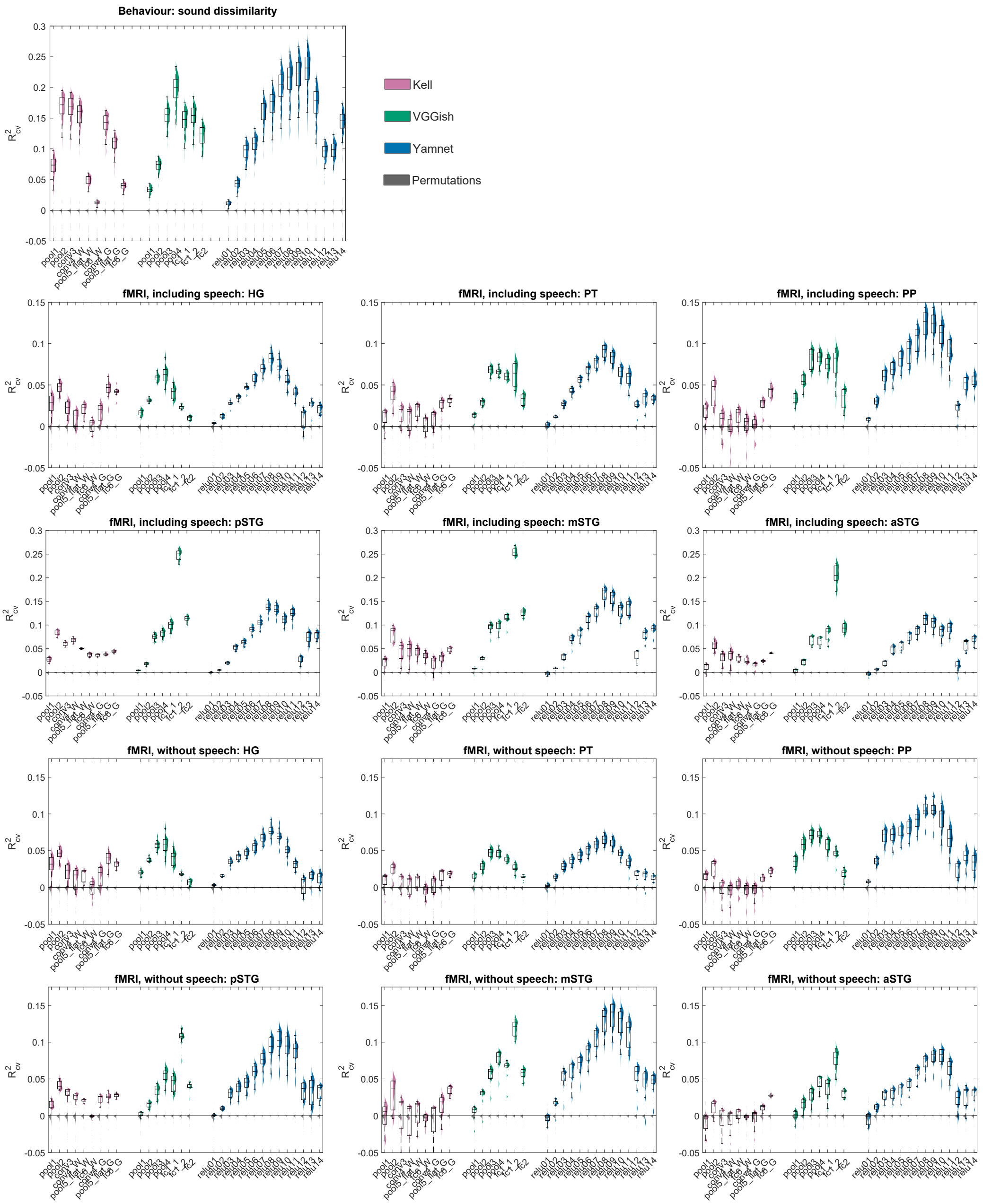

Supplementary Fig. 9: **Layer-by-layer analysis of DNN representation in perceived sound dissimilarity and fMRI data.** Coloured distributions = plugin distribution of R^2_{CV} across CV folds (and corresponding box-plot : centre = median; lower/upper box limits = 1st/3rd quartile; bottom/top whisker = data within 1.5 interquartile ranges from 1st and 3rd quartiles, respectively); dark grey = cross-CV fold median of the permutation results. HG = Heschl's gyrus; PT = planum temporale' PP = planum polare; p/m/aSTG = posterior, mid or anterior portion of the superior temporal gyrus. N sound dissimilarity and fMRI participants = 20 and 5, respectively.

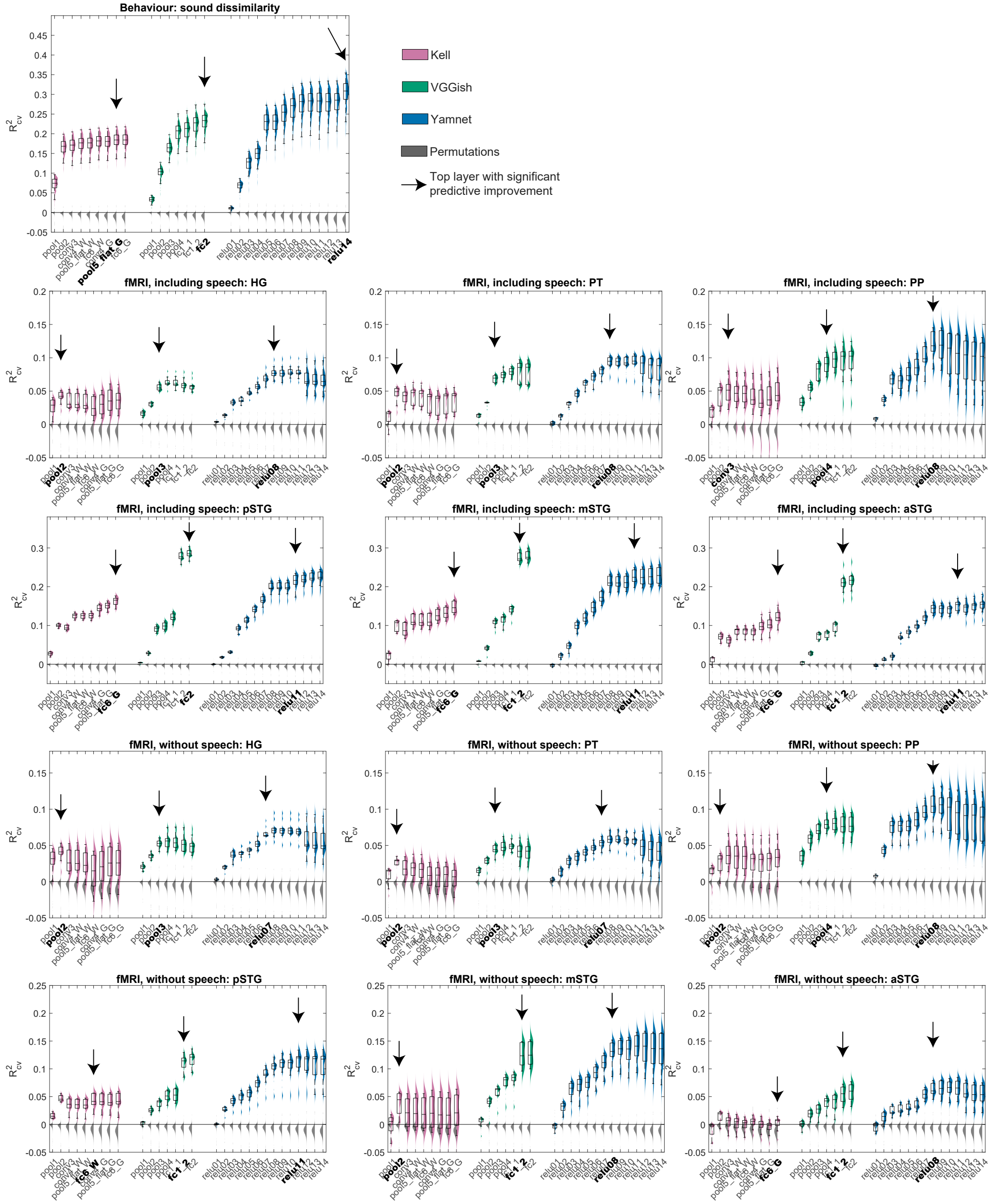

Supplementary Fig. 10: **Layer-cumulative analysis of DNN representation in perceived sound dissimilarity and fMRI data.** Arrows and bold fonts on the x-axis labels indicate the top DNN layer for which we observed a significant improvement in the predictive power when the layer is added to all previous layers ($p < 0.05$, one-sided, adjusted for multiple comparisons across same-DNN layers and fMRI ROIs). Coloured distributions = plugin distribution of R^2_{CV} across CV folds (and corresponding box-plot : centre = median; lower/upper box limits = 1st/3rd quartile; bottom/top whisker = data within 1.5 interquartile ranges from 1st and 3rd quartiles, respectively); dark grey = cross-CV fold median of the permutation results. HG = Heschl's gyrus; PT = planum temporale' PP = planum polare; p/m/aSTG = posterior, mid or anterior portion of the superior temporal gyrus. N sound dissimilarity and fMRI participants = 20 and 5, respectively.

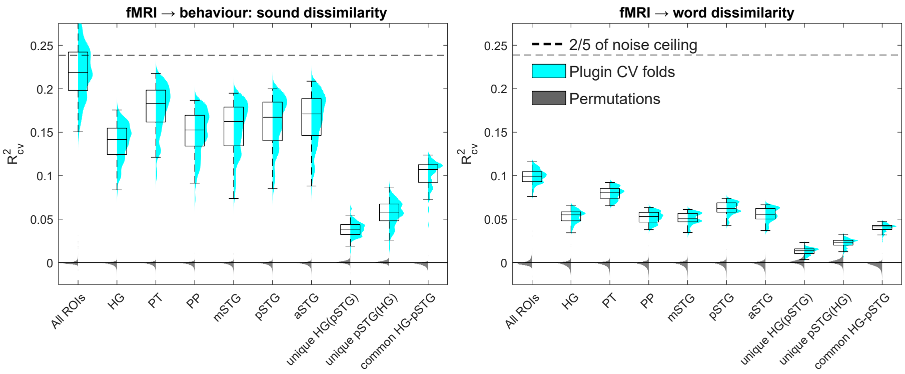

Supplementary Fig. 11: **Prediction of behavioural data from 7T DNN-weighted fMRI data (speech fMRI stimuli excluded).** Left panel: perceived sound dissimilarity task. Right panel: perceived word dissimilarity task. Cyan = plugin distribution of R^2_{CV} across CV folds, each with a corresponding box-plot (centre = median; lower/upper box limits = 1st/3rd quartile; bottom/top whisker = data within 1.5 interquartile ranges from 1st and 3rd quartiles, respectively); dark grey = cross-CV fold median of the permutation analyses; dashed line = 2/5 (40%) of across-fold median noise-ceiling R^2_{CV}. HG = Heschl's gyrus; PT = planum temporale; PP = planum polare; m/p/aSTG = mid/posterior/anterior superior temporal gyrus. Unique = unique behaviour-predictive variance; common = common behaviour-predictive variance (HG + STG analysis). N sound or word dissimilarity participants = 20.

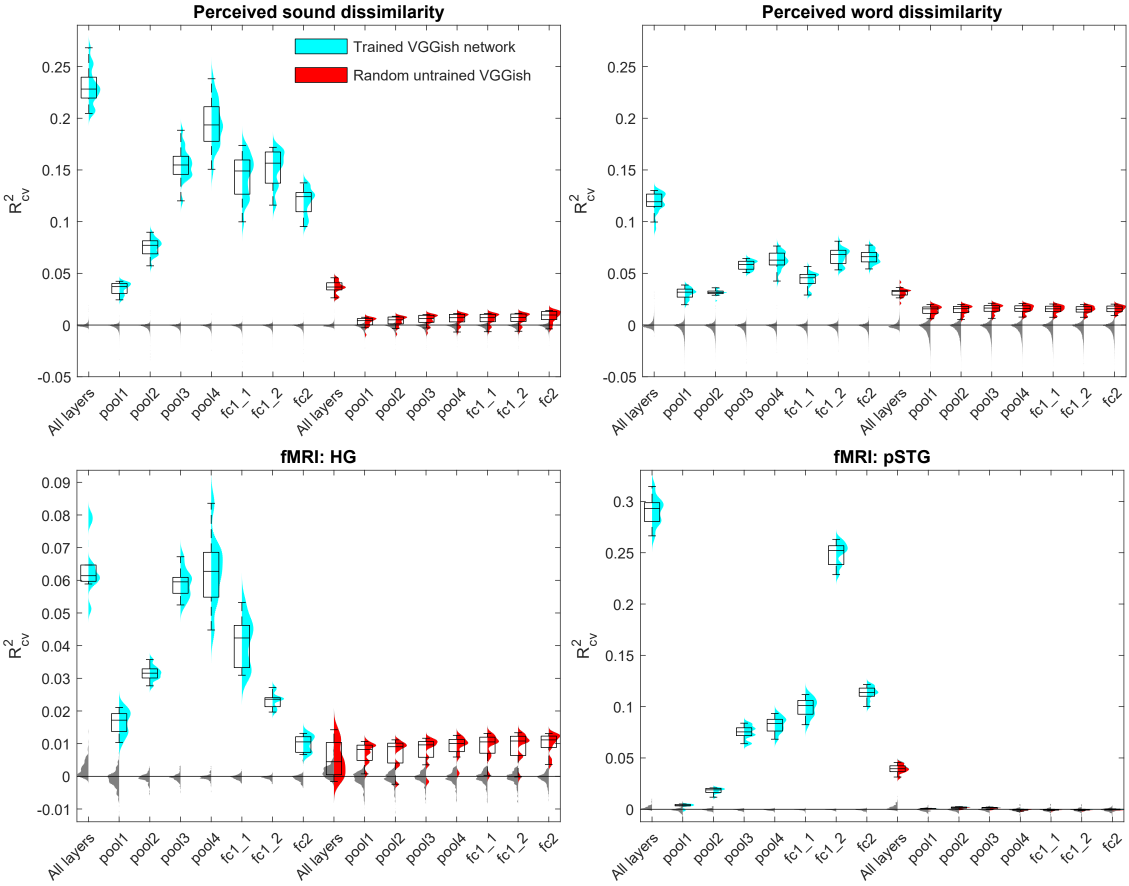

Supplementary Fig. 12: **Behaviour and fMRI predictivity of DNNs in the absence of event-categorization training.** We compare the behaviour and fMRI predictivity of the trained VGGish DNN to that of a random VGGish network (Kaiming He initialization; He et al., 2015). Colours = plugin distribution of R^2_{CV} across CV folds, each with a corresponding box-plot (centre = median; lower/upper box limits = 1st/3rd quartile; bottom/top whisker = data within 1.5 interquartile ranges from 1st and 3rd quartiles, respectively); dark grey = cross-CV fold median of the permutation analyses. HG = Heschl's gyrus; pSTG = posterior superior temporal gyrus. N sound or word dissimilarity participants = 20. N fMRI participants = 5.

He, K., Zhang, X., Ren, S. & Sun, J. Delving deep into rectifiers: Surpassing human-level performance on ImageNet classification. *Proc IEEE Int Conf Computer Vision*, 1026–1034 (2015).

Supplementary Table 1: **Analysis of behavioural data partitioning the unique and common variances across acoustic, semantic and DNN models.** For each behavioural datasets and model, we show the median R^2_{CV} value across CV folds for a particular model, variance partition, or contrast followed, in parentheses, by the interquartile-range of R^2_{CV} across folds, and by the permutation-based p-value for the effect or contrast. Aco = acoustic models; Sem = semantic models; DNN = deep neural network sound-to-event models; u = unique predictive variance; c = common predictive variance. cAll = predictive variance common to the acoustics, semantics and DNN models. Multiple-comparison corrections (FWER = 0.05) applied between: the Aco, Sem and DNN models and pairwise contrasts; the unique predictive variances for the Aco, Sem and DNN models and pairwise contrasts; the common predictive variances and pairwise contrasts. All statistical tests are one-sided, with the exception of the contrasts, which are two-sided. N sound or word dissimilarity participants = 20.

	Sound dissimilarity	Word dissimilarity
Full	0.466(0.038; 0.0001)	0.482(0.035; 0.0001)
Aco	0.250(0.029; 0.0001)	0.115(0.013; 0.0001)
Sem	0.236(0.036; 0.0001)	0.411(0.033; 0.0001)
DNN	0.315(0.047; 0.0001)	0.193(0.021; 0.0001)
uAco	0.029(0.013; 0.0002)	0.013(0.008; 0.0636)
uSem	0.108(0.025; 0.0001)	0.257(0.025; 0.0001)
uDNN	0.072(0.031; 0.0001)	0.034(0.012; 0.0006)
cAcoSem	0.021(0.005; 0.0006)	0.023(0.004; 0.0072)
cAcoDNN	0.138(0.030; 0.0001)	0.027(0.007; 0.0027)
cSemDNN	0.049(0.009; 0.0001)	0.078(0.009; 0.0001)
cAll	0.060(0.004; 0.0001)	0.053(0.005; 0.0001)
Aco vs Sem	0.012(0.061; 0.9986)	-0.297(0.034; 0.0001)
Aco vs DNN	-0.065(0.039; 0.0415)	-0.075(0.021; 0.2324)
Sem vs DNN	-0.072(0.076; 0.0182)	0.221(0.033; 0.0001)
uAco vs uSem	-0.076(0.027; 0.0062)	-0.245(0.029; 0.0001)
uAco vs uDNN	-0.038(0.039; 0.6029)	-0.020(0.015; 0.9922)
uSem vs uDNN	0.038(0.048; 0.6043)	0.225(0.029; 0.0001)
cAcoSem vs cAcoDNN	-0.117(0.029; 0.0001)	-0.004(0.007; 0.8989)
cAcoSem vs cSemDNN	-0.028(0.009; 0.0005)	-0.055(0.010; 0.0010)
cAcoSem vs cAll	-0.038(0.005; 0.0001)	-0.030(0.007; 0.0359)
cAcoDNN vs cSemDNN	0.091(0.038; 0.0001)	-0.051(0.010; 0.0022)
cAcoDNN vs cAll	0.077(0.035; 0.0001)	-0.025(0.008; 0.0717)
cSemDNN vs cAll	-0.010(0.009; 0.1869)	0.025(0.007; 0.0699)

Supplementary Table 2: **Model-by-model analysis of behavioural data.** For each behavioural datasets and model, we show the median R^2_{CV} value across CV folds for a particular model or models contrast followed, in parentheses, by the interquartile-range of R^2_{CV} across folds, and by the permutation-based p-value for the effect or contrast. Multiple-comparison corrections (FWER = 0.05) applied between models from the same class (acoustic, semantics, and deep neural network sound-to-event models), and between pairwise contrasts between same-class models. All statistical tests are one-sided, with the exception of the contrasts, which are two-sided. N sound or word dissimilarity participants = 20.

	Sound dissimilarity	Word dissimilarity
CochleaG	0.014(0.005; 0.0001)	0.008(0.003; 0.0155)
MTF	0.066(0.017; 0.0001)	0.034(0.007; 0.0001)
SAI	0.019(0.008; 0.0001)	0.010(0.004; 0.0084)
Texture	0.069(0.020; 0.0001)	0.015(0.005; 0.0016)
AudDims	0.193(0.035; 0.0001)	0.086(0.011; 0.0001)
GNewsW2V	0.215(0.027; 0.0001)	0.386(0.031; 0.0001)
GloVe	0.160(0.024; 0.0001)	0.247(0.026; 0.0001)
GUSE	0.175(0.026; 0.0001)	0.263(0.028; 0.0001)
Kell	0.178(0.033; 0.0001)	0.084(0.014; 0.0001)
VGGish	0.225(0.038; 0.0001)	0.120(0.013; 0.0001)
Yamnet	0.292(0.064; 0.0001)	0.152(0.022; 0.0001)
CochleaG vs MTF	-0.052(0.016; 0.0043)	-0.026(0.008; 0.2829)
CochleaG vs SAI	-0.005(0.005; 0.9881)	-0.002(0.003; 0.9998)
CochleaG vs Texture	-0.057(0.017; 0.0025)	-0.008(0.005; 0.9722)
CochleaG vs AudDims	-0.183(0.033; 0.0001)	-0.079(0.010; 0.0002)
MTF vs SAI	0.047(0.014; 0.0089)	0.024(0.008; 0.3350)
MTF vs Texture	-0.002(0.019; 0.9999)	0.020(0.009; 0.5147)
MTF vs AudDims	-0.130(0.027; 0.0001)	-0.052(0.015; 0.0088)
SAI vs Texture	-0.051(0.018; 0.0051)	-0.006(0.005; 0.9903)
SAI vs AudDims	-0.177(0.034; 0.0001)	-0.077(0.012; 0.0003)
Texture vs AudDims	-0.126(0.033; 0.0001)	-0.072(0.011; 0.0004)
GNewsW2V vs GloVe	0.056(0.017; 0.0001)	0.137(0.014; 0.0001)
GNewsW2V vs GUSE	0.042(0.015; 0.0001)	0.125(0.024; 0.0001)
GloVe vs GUSE	-0.015(0.014; 0.0004)	-0.012(0.018; 0.0299)
Kell vs VGGish	-0.048(0.019; 0.0003)	-0.035(0.010; 0.1445)
Kell vs Yamnet	-0.116(0.031; 0.0001)	-0.070(0.016; 0.0026)
VGGish vs Yamnet	-0.068(0.030; 0.0001)	-0.035(0.023; 0.1320)

Supplementary Table 3: **Analysis of fMRI data partitioning the unique and common variances across acoustic, semantic and DNN models (speech stimuli included).** For each fMRI ROI and model, we show the median R^2_{CV} value across CV folds for a particular model, variance partition, or contrast followed, in parentheses, by the interquartile-range of R^2_{CV} across folds, and by the permutation-based p-value for the effect or contrast. Aco = acoustic models; Sem = semantic models; DNN = deep neural network sound-to-event models; u = unique predictive variance; c = common predictive variance. cAll = predictive variance common to the acoustics, semantics and DNN models. Multiple-comparison corrections (FWER = 0.05) applied between ROIs and: the Aco, Sem and DNN models and pairwise contrasts; the unique predictive variances for the Aco, Sem and DNN models and pairwise contrasts; the common predictive variances and pairwise contrasts. All statistical tests are one-sided, with the exception of the contrasts, which are two-sided. N fMRI participants = 5.

	fMRI: HG	fMRI: PT	fMRI: PP	fMRI: mSTG	fMRI: pSTG	fMRI: aSTG
Full	0.063(0.028; 0.0001)	0.074(0.055; 0.0001)	0.076(0.061; 0.0001)	0.363(0.040; 0.0001)	0.397(0.022; 0.0001)	0.267(0.037; 0.0001)
Aco	0.060(0.019; 0.0001)	0.035(0.014; 0.0001)	0.032(0.017; 0.0001)	0.139(0.012; 0.0001)	0.159(0.016; 0.0001)	0.101(0.028; 0.0001)
Sem	0.018(0.005; 0.0001)	0.046(0.012; 0.0001)	0.057(0.015; 0.0001)	0.143(0.015; 0.0001)	0.144(0.008; 0.0001)	0.113(0.025; 0.0001)
DNN	0.058(0.019; 0.0001)	0.078(0.056; 0.0001)	0.083(0.047; 0.0001)	0.332(0.038; 0.0001)	0.352(0.029; 0.0001)	0.243(0.038; 0.0001)
uAco	0.012(0.008; 0.0013)	0.002(0.004; 0.7479)	-0.005(0.011; 1.0000)	0.023(0.005; 0.0001)	0.028(0.004; 0.0001)	0.014(0.005; 0.0004)
uSem	-0.003(0.004; 1.0000)	-0.004(0.005; 1.0000)	-0.002(0.004; 1.0000)	0.005(0.003; 0.0828)	0.013(0.001; 0.0010)	0.010(0.003; 0.0040)
uDNN	0.003(0.024; 0.3310)	0.021(0.028; 0.0001)	0.021(0.045; 0.0001)	0.132(0.020; 0.0001)	0.143(0.011; 0.0001)	0.097(0.014; 0.0001)
cAcoSem	0.000(0.001; 0.9999)	0.001(0.001; 0.9601)	0.000(0.001; 0.9975)	0.003(0.001; 0.3422)	0.004(0.000; 0.2367)	0.001(0.002; 0.7680)
cAcoDNN	0.034(0.010; 0.0001)	0.011(0.017; 0.0023)	0.007(0.015; 0.0280)	0.071(0.014; 0.0001)	0.079(0.009; 0.0001)	0.044(0.025; 0.0001)
cSemDNN	0.008(0.005; 0.0226)	0.027(0.006; 0.0001)	0.035(0.004; 0.0001)	0.086(0.007; 0.0001)	0.083(0.005; 0.0001)	0.066(0.011; 0.0001)
cAll	0.014(0.003; 0.0003)	0.021(0.003; 0.0001)	0.023(0.010; 0.0001)	0.046(0.005; 0.0001)	0.046(0.006; 0.0001)	0.037(0.006; 0.0001)
Aco vs Sem	0.044(0.016; 0.0544)	-0.010(0.019; 1.0000)	-0.032(0.010; 0.5566)	-0.004(0.014; 1.0000)	0.012(0.009; 1.0000)	-0.016(0.021; 0.9977)
Aco vs DNN	-0.001(0.035; 1.0000)	-0.042(0.036; 0.0809)	-0.054(0.044; 0.0027)	-0.195(0.021; 0.0001)	-0.200(0.012; 0.0001)	-0.149(0.011; 0.0001)
Sem vs DNN	-0.036(0.026; 0.2746)	-0.035(0.048; 0.3317)	-0.026(0.048; 0.8370)	-0.187(0.036; 0.0001)	-0.205(0.013; 0.0001)	-0.129(0.032; 0.0001)
uAco vs uSem	0.017(0.008; 0.9920)	0.004(0.006; 1.0000)	-0.005(0.009; 1.0000)	0.016(0.006; 0.9945)	0.016(0.004; 0.9954)	0.004(0.004; 1.0000)
uAco vs uDNN	0.007(0.037; 1.0000)	-0.019(0.032; 0.9801)	-0.017(0.052; 0.9907)	-0.113(0.018; 0.0001)	-0.115(0.007; 0.0001)	-0.087(0.016; 0.0001)
uSem vs uDNN	-0.007(0.019; 1.0000)	-0.028(0.023; 0.6736)	-0.021(0.048; 0.9457)	-0.125(0.020; 0.0001)	-0.129(0.012; 0.0001)	-0.089(0.016; 0.0001)
cAcoSem vs cAcoDNN	-0.034(0.011; 0.0001)	-0.011(0.017; 0.0451)	-0.007(0.015; 0.3593)	-0.068(0.014; 0.0001)	-0.075(0.009; 0.0001)	-0.042(0.025; 0.0001)
cAcoSem vs cSemDNN	-0.007(0.005; 0.2451)	-0.027(0.005; 0.0001)	-0.035(0.004; 0.0001)	-0.082(0.006; 0.0001)	-0.079(0.006; 0.0001)	-0.064(0.010; 0.0001)
cAcoSem vs cAll	-0.014(0.003; 0.0065)	-0.021(0.003; 0.0001)	-0.023(0.011; 0.0001)	-0.043(0.003; 0.0001)	-0.042(0.004; 0.0001)	-0.036(0.004; 0.0001)
cAcoDNN vs cSemDNN	0.024(0.012; 0.0001)	-0.015(0.018; 0.0031)	-0.028(0.017; 0.0001)	-0.018(0.013; 0.0004)	-0.004(0.011; 0.8270)	-0.022(0.024; 0.0001)
cAcoDNN vs cAll	0.018(0.010; 0.0004)	-0.011(0.015; 0.0394)	-0.014(0.017; 0.0053)	0.024(0.013; 0.0001)	0.036(0.010; 0.0001)	0.007(0.026; 0.3630)
cSemDNN vs cAll	-0.006(0.003; 0.4205)	0.006(0.004; 0.5002)	0.013(0.004; 0.0092)	0.038(0.004; 0.0001)	0.037(0.008; 0.0001)	0.029(0.006; 0.0001)

Supplementary Table 4: **Model-by-model analysis of fMRI data (speech stimuli included).** For each fMRI ROI and model, we show the median R^2_{CV} value across CV folds for a particular model, or contrast followed, in parentheses, by the interquartile-range of R^2_{CV} across folds, and by the permutation-based p-value for the effect or contrast. Multiple-comparison corrections (FWER = 0.05) applied between ROIs and between: models from the same class (acoustic, semantics, and deep neural network sound-to-event models); pairwise contrasts between same-class models. All statistical tests are one-sided, with the exception of the contrasts, which are two-sided. N fMRI participants = 5.

	fMRI: HG	fMRI: PT	fMRI: PP	fMRI: mSTG	fMRI: pSTG	fMRI: aSTG
MTF	0.058(0.015; 0.0001)	0.011(0.008; 0.0019)	0.019(0.003; 0.0003)	0.014(0.006; 0.0007)	0.017(0.003; 0.0004)	0.004(0.005; 0.0737)
SAI	0.033(0.008; 0.0001)	0.010(0.009; 0.0027)	0.013(0.003; 0.0008)	0.013(0.004; 0.0011)	0.015(0.002; 0.0007)	0.010(0.004; 0.0035)
Texture	0.022(0.006; 0.0003)	0.009(0.002; 0.0056)	0.010(0.003; 0.0027)	0.008(0.003; 0.0076)	0.013(0.002; 0.0011)	0.009(0.002; 0.0056)
AudDims	0.040(0.007; 0.0001)	0.024(0.008; 0.0001)	0.028(0.017; 0.0001)	0.092(0.008; 0.0001)	0.098(0.013; 0.0001)	0.067(0.022; 0.0001)
GNewsW2V	0.021(0.003; 0.0003)	0.045(0.006; 0.0001)	0.056(0.015; 0.0001)	0.129(0.012; 0.0001)	0.127(0.012; 0.0001)	0.098(0.009; 0.0001)
GloVe	0.013(0.005; 0.0011)	0.029(0.004; 0.0001)	0.035(0.014; 0.0001)	0.077(0.011; 0.0001)	0.075(0.011; 0.0001)	0.057(0.004; 0.0001)
GUSE	0.017(0.003; 0.0004)	0.028(0.003; 0.0001)	0.037(0.010; 0.0001)	0.069(0.007; 0.0001)	0.062(0.005; 0.0001)	0.049(0.003; 0.0001)
Categ	0.018(0.003; 0.0004)	0.033(0.006; 0.0001)	0.051(0.013; 0.0001)	0.114(0.010; 0.0001)	0.109(0.012; 0.0001)	0.091(0.009; 0.0001)
Kell	0.036(0.024; 0.0001)	0.043(0.028; 0.0001)	0.043(0.027; 0.0001)	0.146(0.030; 0.0001)	0.165(0.016; 0.0001)	0.120(0.021; 0.0001)
VGGish	0.057(0.005; 0.0001)	0.086(0.031; 0.0001)	0.102(0.026; 0.0001)	0.276(0.019; 0.0001)	0.285(0.014; 0.0001)	0.216(0.021; 0.0001)
Yamnet	0.064(0.016; 0.0001)	0.088(0.032; 0.0001)	0.102(0.057; 0.0001)	0.229(0.041; 0.0001)	0.224(0.018; 0.0001)	0.155(0.015; 0.0001)
CochleaG vs MTF	-0.027(0.011; 0.0001)	-0.003(0.007; 0.9976)	-0.008(0.004; 0.3035)	-0.007(0.007; 0.4543)	-0.010(0.005; 0.0754)	0.003(0.007; 0.9915)
CochleaG vs SAI	-0.005(0.005; 0.8380)	-0.002(0.008; 1.0000)	-0.002(0.004; 1.0000)	-0.007(0.005; 0.4503)	-0.010(0.003; 0.0779)	-0.004(0.005; 0.9060)
CochleaG vs Texture	0.008(0.003; 0.2914)	-0.001(0.002; 1.0000)	0.002(0.003; 1.0000)	-0.002(0.004; 1.0000)	-0.008(0.001; 0.3214)	-0.003(0.005; 0.9973)
CochleaG vs AudDims	-0.012(0.003; 0.0252)	-0.016(0.008; 0.0021)	-0.017(0.014; 0.0017)	-0.086(0.007; 0.0001)	-0.090(0.013; 0.0001)	-0.060(0.024; 0.0001)
MTF vs SAI	0.022(0.004; 0.0003)	0.001(0.003; 1.0000)	0.006(0.004; 0.6180)	0.001(0.004; 1.0000)	-0.000(0.006; 1.0000)	-0.005(0.004; 0.7362)
MTF vs Texture	0.036(0.012; 0.0001)	0.002(0.009; 1.0000)	0.008(0.007; 0.2027)	0.006(0.007; 0.5420)	0.003(0.004; 0.9958)	-0.004(0.010; 0.8969)
MTF vs AudDims	0.016(0.013; 0.0017)	-0.014(0.007; 0.0042)	-0.010(0.005; 0.0993)	-0.078(0.007; 0.0001)	-0.082(0.016; 0.0001)	-0.061(0.018; 0.0001)
SAI vs Texture	0.013(0.007; 0.0170)	0.001(0.011; 1.0000)	0.003(0.003; 0.9993)	0.005(0.007; 0.7384)	0.002(0.004; 0.9994)	0.002(0.006; 1.0000)
SAI vs AudDims	-0.007(0.007; 0.3883)	-0.016(0.007; 0.0024)	-0.014(0.013; 0.0059)	-0.077(0.009; 0.0001)	-0.080(0.012; 0.0001)	-0.053(0.016; 0.0001)
Texture vs AudDims	-0.020(0.004; 0.0004)	-0.016(0.012; 0.0021)	-0.018(0.017; 0.0004)	-0.083(0.010; 0.0001)	-0.083(0.014; 0.0001)	-0.057(0.030; 0.0001)
GNewsW2V vs GloVe	0.009(0.001; 0.0306)	0.018(0.003; 0.0001)	0.020(0.004; 0.0001)	0.050(0.006; 0.0001)	0.052(0.005; 0.0001)	0.042(0.008; 0.0001)
GNewsW2V vs GUSE	0.004(0.002; 0.7265)	0.018(0.002; 0.0001)	0.018(0.006; 0.0001)	0.059(0.007; 0.0001)	0.065(0.006; 0.0001)	0.050(0.008; 0.0001)
GNewsW2V vs Categ	0.003(0.004; 0.9261)	0.010(0.004; 0.0052)	0.003(0.007; 0.9047)	0.011(0.009; 0.0034)	0.016(0.007; 0.0001)	0.007(0.004; 0.1185)
GloVe vs GUSE	-0.004(0.001; 0.7324)	0.001(0.003; 1.0000)	-0.001(0.002; 0.9999)	0.009(0.002; 0.0292)	0.012(0.004; 0.0017)	0.008(0.001; 0.0488)
GloVe vs Categ	-0.005(0.004; 0.5250)	-0.007(0.006; 0.1612)	-0.016(0.009; 0.0001)	-0.040(0.005; 0.0001)	-0.037(0.005; 0.0001)	-0.035(0.006; 0.0001)
GUSE vs Categ	-0.001(0.005; 1.0000)	-0.007(0.005; 0.2055)	-0.013(0.007; 0.0004)	-0.047(0.003; 0.0001)	-0.049(0.008; 0.0001)	-0.043(0.007; 0.0001)
Kell vs VGGish	-0.018(0.014; 0.0852)	-0.044(0.006; 0.0001)	-0.044(0.022; 0.0001)	-0.133(0.006; 0.0001)	-0.124(0.023; 0.0001)	-0.094(0.018; 0.0001)
Kell vs Yamnet	-0.028(0.010; 0.0012)	-0.046(0.009; 0.0001)	-0.054(0.029; 0.0001)	-0.081(0.016; 0.0001)	-0.064(0.018; 0.0001)	-0.028(0.018; 0.0012)
VGGish vs Yamnet	-0.011(0.016; 0.6737)	-0.003(0.012; 1.0000)	-0.003(0.014; 1.0000)	0.051(0.020; 0.0001)	0.064(0.017; 0.0001)	0.058(0.017; 0.0001)

Supplementary Table 5: **Analysis of fMRI data partitioning the unique and common variances across acoustic, semantic and DNN models (speech stimuli excluded).** For each fMRI ROI and model, we show the median R^2_{CV} value across CV folds for a particular model, or contrast followed, in parentheses, by the interquartile-range of R^2_{CV} across folds, and by the permutation-based p-value for the effect or contrast. Aco = acoustic models; Sem = semantic models; DNN = deep neural network sound-to-event models; u = unique predictive variance; c = common predictive variance. cAll = predictive variance common to the acoustics, semantics and DNN models. Multiple-comparison corrections (FWER = 0.05) applied between ROIs and: the Aco, Sem and DNN models and pairwise contrasts; the unique predictive variances for the Aco, Sem and DNN models and pairwise contrasts; the common predictive variances and pairwise contrasts. All statistical tests are one-sided, with the exception of the contrasts, which are two-sided. N fMRI participants = 5.

	fMRI: HG	fMRI: PT	fMRI: PP	fMRI: mSTG	fMRI: pSTG	fMRI: aSTG
Full	0.044(0.024; 0.0001)	0.012(0.051; 0.0040)	0.048(0.042; 0.0001)	0.120(0.071; 0.0001)	0.134(0.038; 0.0001)	0.024(0.020; 0.0001)
Aco	0.057(0.021; 0.0001)	0.019(0.015; 0.0002)	0.022(0.011; 0.0001)	0.034(0.026; 0.0001)	0.034(0.023; 0.0001)	-0.001(0.038; 1.0000)
Sem	0.003(0.006; 0.4476)	0.009(0.006; 0.0166)	0.019(0.008; 0.0002)	0.047(0.009; 0.0001)	0.045(0.004; 0.0001)	0.033(0.006; 0.0001)
DNN	0.035(0.026; 0.0001)	0.018(0.047; 0.0002)	0.059(0.042; 0.0001)	0.123(0.066; 0.0001)	0.125(0.027; 0.0001)	0.018(0.018; 0.0002)
uAco	0.012(0.013; 0.0037)	0.006(0.002; 0.1297)	-0.004(0.007; 1.0000)	0.005(0.006; 0.1731)	0.008(0.007; 0.0461)	0.002(0.006; 0.6288)
uSem	-0.002(0.005; 1.0000)	-0.005(0.006; 1.0000)	-0.002(0.003; 1.0000)	-0.006(0.003; 1.0000)	-0.001(0.003; 1.0000)	-0.000(0.002; 1.0000)
uDNN	-0.004(0.018; 1.0000)	-0.007(0.034; 1.0000)	0.019(0.038; 0.0002)	0.067(0.045; 0.0001)	0.071(0.020; 0.0001)	0.011(0.024; 0.0100)
cAcoSem	-0.001(0.001; 1.0000)	-0.001(0.001; 1.0000)	0.000(0.001; 0.9999)	0.001(0.001; 0.8612)	0.002(0.001; 0.6781)	-0.000(0.001; 0.9999)
cAcoDNN	0.041(0.015; 0.0001)	0.008(0.014; 0.0316)	0.018(0.008; 0.0002)	0.010(0.017; 0.0140)	0.012(0.013; 0.0047)	-0.011(0.035; 1.0000)
cSemDNN	0.001(0.002; 0.8544)	0.008(0.002; 0.0457)	0.011(0.003; 0.0090)	0.035(0.008; 0.0001)	0.034(0.005; 0.0001)	0.023(0.004; 0.0001)
cAll	0.006(0.002; 0.0826)	0.008(0.002; 0.0367)	0.011(0.003; 0.0067)	0.016(0.002; 0.0005)	0.011(0.004; 0.0060)	0.011(0.002; 0.0101)
Aco vs Sem	0.057(0.019; 0.0416)	0.012(0.014; 1.0000)	0.005(0.011; 1.0000)	-0.009(0.020; 1.0000)	-0.010(0.023; 1.0000)	-0.030(0.031; 0.9314)
Aco vs DNN	0.014(0.037; 1.0000)	0.002(0.031; 1.0000)	-0.034(0.036; 0.8160)	-0.102(0.038; 0.0001)	-0.099(0.021; 0.0001)	-0.030(0.031; 0.9170)
Sem vs DNN	-0.031(0.037; 0.9022)	-0.009(0.045; 1.0000)	-0.043(0.045; 0.4212)	-0.078(0.057; 0.0002)	-0.081(0.026; 0.0001)	0.013(0.019; 1.0000)
uAco vs uSem	0.015(0.012; 0.9999)	0.011(0.003; 1.0000)	-0.002(0.007; 1.0000)	0.011(0.007; 1.0000)	0.009(0.007; 1.0000)	0.004(0.005; 1.0000)
uAco vs uDNN	0.019(0.033; 0.9974)	0.010(0.033; 1.0000)	-0.023(0.038; 0.9842)	-0.065(0.044; 0.0018)	-0.064(0.021; 0.0021)	-0.008(0.029; 1.0000)
uSem vs uDNN	-0.001(0.019; 1.0000)	-0.002(0.032; 1.0000)	-0.024(0.040; 0.9775)	-0.073(0.043; 0.0002)	-0.072(0.017; 0.0002)	-0.012(0.025; 1.0000)
cAcoSem vs cAcoDNN	-0.042(0.015; 0.0001)	-0.009(0.014; 0.2300)	-0.018(0.009; 0.0030)	-0.009(0.017; 0.2864)	-0.011(0.012; 0.1368)	0.011(0.034; 0.1146)
cAcoSem vs cSemDNN	-0.002(0.002; 0.9971)	-0.008(0.003; 0.3679)	-0.011(0.003; 0.1315)	-0.033(0.007; 0.0001)	-0.032(0.004; 0.0001)	-0.023(0.004; 0.0002)
cAcoSem vs cAll	-0.008(0.001; 0.4170)	-0.009(0.002; 0.2786)	-0.011(0.004; 0.1041)	-0.015(0.002; 0.0120)	-0.010(0.005; 0.2013)	-0.010(0.002; 0.1963)
cAcoDNN vs cSemDNN	0.040(0.018; 0.0001)	0.001(0.017; 1.0000)	0.008(0.008; 0.3925)	-0.021(0.018; 0.0004)	-0.021(0.012; 0.0003)	-0.034(0.030; 0.0001)
cAcoDNN vs cAll	0.034(0.015; 0.0001)	0.000(0.012; 1.0000)	0.007(0.010; 0.5950)	-0.007(0.018; 0.5957)	0.002(0.014; 1.0000)	-0.021(0.035; 0.0005)
cSemDNN vs cAll	-0.005(0.003; 0.7777)	-0.001(0.003; 1.0000)	-0.001(0.002; 1.0000)	0.019(0.008; 0.0022)	0.023(0.005; 0.0001)	0.012(0.005; 0.0636)

Supplementary Table 6: **Model-by-model analysis of fMRI data (speech stimuli excluded).** For each fMRI ROI and model, we show the median R^2_{CV} value across CV folds for a particular model or models contrast followed, in parentheses, by the interquartile-range of R^2_{CV} across folds, and by the permutation-based p-value for the effect or contrast. Multiple-comparison corrections (FWER = 0.05) applied between ROIs and between: models from the same class (acoustic, semantics, and deep neural network sound-to-event models); pairwise contrasts between same-class models. All statistical tests are one-sided, with the exception of the contrasts, which are two-sided. N fMRI participants = 5.

	fMRI: HG	fMRI: PT	fMRI: PP	fMRI: mSTG	fMRI: pSTG	fMRI: aSTG
CochleaG	0.031(0.006; 0.0001)	0.009(0.002; 0.0107)	0.009(0.004; 0.0124)	0.001(0.001; 0.5592)	-0.000(0.001; 0.9999)	0.001(0.003; 0.7655)
MTF	0.060(0.019; 0.0001)	0.014(0.010; 0.0011)	0.020(0.005; 0.0003)	0.004(0.008; 0.1139)	0.002(0.005; 0.3203)	-0.006(0.011; 1.0000)
SAI	0.037(0.006; 0.0001)	0.009(0.005; 0.0110)	0.011(0.002; 0.0044)	0.000(0.006; 0.9783)	0.001(0.002; 0.7286)	-0.002(0.003; 1.0000)
Texture	0.019(0.007; 0.0003)	0.009(0.003; 0.0075)	0.007(0.006; 0.0246)	0.002(0.003; 0.3074)	0.002(0.004; 0.2867)	0.000(0.003; 0.9217)
AudDims	0.034(0.010; 0.0001)	0.009(0.007; 0.0075)	0.023(0.006; 0.0003)	0.036(0.022; 0.0001)	0.030(0.014; 0.0001)	0.013(0.033; 0.0018)
GNewsW2V	0.007(0.003; 0.0221)	0.016(0.002; 0.0006)	0.021(0.006; 0.0003)	0.048(0.010; 0.0001)	0.047(0.003; 0.0001)	0.035(0.005; 0.0001)
GloVe	0.004(0.002; 0.0985)	0.011(0.003; 0.0031)	0.017(0.006; 0.0004)	0.037(0.008; 0.0001)	0.036(0.003; 0.0001)	0.026(0.004; 0.0002)
GUSE	0.007(0.002; 0.0275)	0.012(0.003; 0.0027)	0.016(0.006; 0.0005)	0.038(0.009; 0.0001)	0.032(0.002; 0.0001)	0.023(0.002; 0.0003)
Categ	0.009(0.003; 0.0098)	0.018(0.005; 0.0003)	0.030(0.012; 0.0001)	0.075(0.012; 0.0001)	0.071(0.012; 0.0001)	0.057(0.017; 0.0001)
Kell	0.026(0.034; 0.0002)	0.007(0.022; 0.0291)	0.034(0.024; 0.0001)	0.021(0.054; 0.0003)	0.041(0.019; 0.0001)	0.007(0.010; 0.0270)
VGGish	0.049(0.012; 0.0001)	0.042(0.016; 0.0001)	0.076(0.020; 0.0001)	0.125(0.041; 0.0001)	0.121(0.018; 0.0001)	0.060(0.026; 0.0001)
Yamnet	0.049(0.021; 0.0001)	0.042(0.025; 0.0001)	0.089(0.047; 0.0001)	0.136(0.054; 0.0001)	0.117(0.033; 0.0001)	0.053(0.028; 0.0001)
CochleaG vs MTF	-0.029(0.010; 0.0002)	-0.006(0.008; 0.9291)	-0.011(0.006; 0.1900)	-0.002(0.008; 1.0000)	-0.003(0.006; 1.0000)	0.006(0.012; 0.9296)
CochleaG vs SAI	-0.005(0.003; 0.9545)	0.001(0.005; 1.0000)	-0.003(0.002; 1.0000)	0.001(0.006; 1.0000)	-0.002(0.003; 1.0000)	0.001(0.002; 1.0000)
CochleaG vs Texture	0.011(0.003; 0.1649)	0.000(0.001; 1.0000)	0.001(0.003; 1.0000)	-0.001(0.002; 1.0000)	-0.002(0.005; 1.0000)	-0.000(0.005; 1.0000)
CochleaG vs AudDims	-0.004(0.005; 0.9989)	-0.001(0.007; 1.0000)	-0.014(0.006; 0.0350)	-0.034(0.022; 0.0001)	-0.030(0.014; 0.0001)	-0.013(0.034; 0.0717)
MTF vs SAI	0.025(0.007; 0.0006)	0.005(0.005; 0.9715)	0.008(0.007; 0.5342)	0.003(0.002; 1.0000)	0.001(0.003; 1.0000)	-0.004(0.006; 0.9974)
MTF vs Texture	0.041(0.012; 0.0001)	0.006(0.008; 0.9089)	0.013(0.007; 0.0685)	0.002(0.009; 1.0000)	0.000(0.005; 1.0000)	-0.007(0.007; 0.7301)
MTF vs AudDims	0.025(0.009; 0.0004)	0.002(0.008; 1.0000)	-0.005(0.007; 0.9663)	-0.031(0.012; 0.0001)	-0.028(0.015; 0.0002)	-0.017(0.024; 0.0084)
SAI vs Texture	0.017(0.003; 0.0086)	-0.001(0.008; 1.0000)	0.004(0.004; 0.9957)	-0.002(0.008; 1.0000)	-0.001(0.003; 1.0000)	-0.003(0.002; 1.0000)
SAI vs AudDims	0.001(0.006; 1.0000)	-0.002(0.007; 1.0000)	-0.013(0.004; 0.0835)	-0.034(0.015; 0.0001)	-0.029(0.013; 0.0002)	-0.013(0.028; 0.0748)
Texture vs AudDims	-0.015(0.007; 0.0238)	-0.002(0.008; 1.0000)	-0.017(0.009; 0.0104)	-0.035(0.022; 0.0001)	-0.028(0.011; 0.0002)	-0.011(0.030; 0.2024)
GNewsW2V vs GloVe	0.004(0.002; 0.9398)	0.005(0.002; 0.8064)	0.006(0.002; 0.6838)	0.009(0.003; 0.1634)	0.012(0.003; 0.0310)	0.008(0.003; 0.2283)
GNewsW2V vs GUSE	0.001(0.001; 1.0000)	0.004(0.002; 0.8734)	0.006(0.002; 0.6020)	0.009(0.001; 0.1944)	0.015(0.002; 0.0022)	0.012(0.003; 0.0302)
GNewsW2V vs Categ	-0.002(0.007; 0.9975)	-0.003(0.004; 0.9841)	-0.010(0.011; 0.0696)	-0.030(0.011; 0.0001)	-0.025(0.009; 0.0001)	-0.025(0.013; 0.0001)
GloVe vs GUSE	-0.002(0.001; 0.9968)	-0.000(0.003; 1.0000)	0.000(0.003; 1.0000)	-0.000(0.002; 1.0000)	0.004(0.002; 0.9264)	0.004(0.001; 0.9336)
GloVe vs Categ	-0.005(0.005; 0.7916)	-0.007(0.003; 0.4330)	-0.015(0.012; 0.0022)	-0.040(0.011; 0.0001)	-0.035(0.011; 0.0001)	-0.032(0.015; 0.0001)
GUSE vs Categ	-0.002(0.005; 0.9979)	-0.007(0.003; 0.4851)	-0.017(0.011; 0.0006)	-0.039(0.012; 0.0001)	-0.040(0.011; 0.0001)	-0.036(0.015; 0.0001)
Kell vs VGGish	-0.022(0.020; 0.0904)	-0.036(0.018; 0.0014)	-0.043(0.032; 0.0002)	-0.101(0.018; 0.0001)	-0.075(0.034; 0.0001)	-0.057(0.015; 0.0001)
Kell vs Yamnet	-0.026(0.018; 0.0231)	-0.037(0.014; 0.0009)	-0.056(0.013; 0.0001)	-0.102(0.027; 0.0001)	-0.068(0.036; 0.0001)	-0.051(0.030; 0.0001)
VGGish vs Yamnet	-0.007(0.019; 0.9868)	-0.004(0.011; 0.9996)	-0.014(0.025; 0.6110)	-0.013(0.027; 0.6868)	0.008(0.022; 0.9816)	0.005(0.027; 0.9989)

Supplementary Table 7: **Layer-level analysis of DNN representation in perceived sound dissimilarity.** For each layer we show : the predictive power of the layer alone (layer-by-layer analysis), the predictive power of the layer along with all preceding layers (layer-cumulative analysis), and the improvement in the predictive power when the layer is added to all previous layers (cumulative improvement analysis). For each of these statistics, we show the median R^2_{CV} value across CV folds followed, in parentheses, by the interquartile-range of R^2_{CV} across folds, and by the permutation-based p-value for the effect. Multiple-comparison corrections (FWER = 0.05) applied between layers from the same DNN. All statistical tests are one-sided. N sound dissimilarity participants = 20.

	Layer-by-layer	Layer-cumulative	Cumulative improvement
Kell: pool1	0.074(0.020; 0.0001)	0.074(0.022; 0.0001)	NaN(NaN; NaN)
Kell: pool2	0.172(0.027; 0.0001)	0.168(0.027; 0.0001)	0.098(0.017; 0.0001)
Kell: conv3	0.170(0.027; 0.0001)	0.171(0.026; 0.0001)	0.005(0.003; 0.0109)
Kell: conv4_W	0.161(0.028; 0.0001)	0.177(0.025; 0.0001)	0.006(0.005; 0.0053)
Kell: pool5_flat_W	0.049(0.011; 0.0001)	0.177(0.025; 0.0001)	0.001(0.001; 0.8627)
Kell: fc6_W	0.013(0.004; 0.0002)	0.182(0.024; 0.0001)	0.005(0.004; 0.0082)
Kell: conv4_G	0.143(0.022; 0.0001)	0.180(0.025; 0.0001)	-0.001(0.001; 1.0000)
Kell: pool5_flat_G	0.113(0.017; 0.0001)	0.184(0.024; 0.0001)	0.005(0.002; 0.0105)
Kell: fc6_G	0.040(0.008; 0.0001)	0.184(0.025; 0.0001)	0.000(0.001; 0.9978)
VGGish: pool1	0.034(0.008; 0.0001)	0.033(0.009; 0.0001)	NaN(NaN; NaN)
VGGish: pool2	0.075(0.013; 0.0001)	0.104(0.015; 0.0001)	0.071(0.013; 0.0001)
VGGish: pool3	0.156(0.021; 0.0001)	0.164(0.021; 0.0001)	0.061(0.010; 0.0001)
VGGish: pool4	0.200(0.030; 0.0001)	0.207(0.032; 0.0001)	0.041(0.013; 0.0001)
VGGish: fc1_1	0.148(0.027; 0.0001)	0.213(0.036; 0.0001)	0.007(0.003; 0.0013)
VGGish: fc1_2	0.154(0.024; 0.0001)	0.228(0.034; 0.0001)	0.014(0.005; 0.0001)
VGGish: fc2	0.126(0.026; 0.0001)	0.234(0.029; 0.0001)	0.009(0.008; 0.0005)
Yamnet: relu01	0.012(0.005; 0.0003)	0.011(0.004; 0.0003)	NaN(NaN; NaN)
Yamnet: relu02	0.043(0.011; 0.0001)	0.070(0.013; 0.0001)	0.060(0.013; 0.0001)
Yamnet: relu03	0.098(0.019; 0.0001)	0.128(0.025; 0.0001)	0.058(0.016; 0.0001)
Yamnet: relu04	0.109(0.019; 0.0001)	0.150(0.025; 0.0001)	0.024(0.006; 0.0001)
Yamnet: relu05	0.163(0.027; 0.0001)	0.231(0.038; 0.0001)	0.083(0.019; 0.0001)
Yamnet: relu06	0.177(0.030; 0.0001)	0.232(0.037; 0.0001)	0.001(0.002; 0.8153)
Yamnet: relu07	0.204(0.035; 0.0001)	0.255(0.042; 0.0001)	0.024(0.008; 0.0001)
Yamnet: relu08	0.217(0.035; 0.0001)	0.271(0.047; 0.0001)	0.015(0.005; 0.0001)
Yamnet: relu09	0.223(0.038; 0.0001)	0.282(0.044; 0.0001)	0.012(0.005; 0.0002)
Yamnet: relu10	0.232(0.036; 0.0001)	0.283(0.044; 0.0001)	0.002(0.002; 0.3338)
Yamnet: relu11	0.179(0.035; 0.0001)	0.283(0.047; 0.0001)	0.000(0.003; 0.9843)
Yamnet: relu12	0.097(0.017; 0.0001)	0.281(0.045; 0.0001)	0.001(0.003; 0.8939)
Yamnet: relu13	0.099(0.021; 0.0001)	0.285(0.041; 0.0001)	0.003(0.004; 0.0590)
Yamnet: relu14	0.146(0.022; 0.0001)	0.309(0.040; 0.0001)	0.024(0.012; 0.0001)

Supplementary Table 8: **Layer-by-layer analysis of DNN representation in fMRI data (speech stimuli included).** For each layer-based model, we show the median R^2_{CV} value across CV folds for a particular layer followed, in parentheses, by the interquartile-range of R^2_{CV} across folds, and by the permutation-based p-value for the effect or contrast. Multiple-comparison corrections (FWER = 0.05) applied between ROIs and layers from the same DNN. All statistical tests are one-sided, with the exception of the contrasts. N fMRI participants = 5.

	fMRI: HG	fMRI: PT	fMRI: PP	fMRI: mSTG	fMRI: pSTG	fMRI: aSTG
Kell: pool1	0.029(0.018; 0.0001)	0.016(0.014; 0.0003)	0.022(0.015; 0.0001)	0.027(0.016; 0.0001)	0.028(0.008; 0.0001)	0.016(0.011; 0.0003)
Kell: pool2	0.049(0.009; 0.0001)	0.043(0.017; 0.0001)	0.048(0.030; 0.0001)	0.089(0.031; 0.0001)	0.083(0.010; 0.0001)	0.060(0.014; 0.0001)
Kell: conv3	0.023(0.014; 0.0001)	0.021(0.018; 0.0001)	0.009(0.017; 0.0055)	0.051(0.027; 0.0001)	0.063(0.008; 0.0001)	0.037(0.015; 0.0001)
Kell: conv4_W	0.013(0.020; 0.0005)	0.018(0.026; 0.0002)	-0.004(0.015; 1.0000)	0.051(0.025; 0.0001)	0.070(0.007; 0.0001)	0.040(0.016; 0.0001)
Kell: pool5_flat_W	0.022(0.011; 0.0001)	0.025(0.015; 0.0001)	0.017(0.015; 0.0002)	0.045(0.014; 0.0001)	0.050(0.001; 0.0001)	0.032(0.010; 0.0001)
Kell: fc6_W	0.004(0.013; 0.1445)	0.009(0.017; 0.0073)	0.006(0.015; 0.0455)	0.036(0.009; 0.0001)	0.039(0.007; 0.0001)	0.023(0.010; 0.0001)
Kell: conv4_G	0.020(0.018; 0.0001)	0.014(0.017; 0.0005)	0.003(0.010; 0.4373)	0.026(0.021; 0.0001)	0.038(0.005; 0.0001)	0.018(0.006; 0.0002)
Kell: pool5_flat_G	0.047(0.010; 0.0001)	0.029(0.009; 0.0001)	0.029(0.009; 0.0001)	0.033(0.012; 0.0001)	0.038(0.003; 0.0001)	0.024(0.003; 0.0001)
Kell: fc6_G	0.042(0.003; 0.0001)	0.033(0.005; 0.0001)	0.044(0.011; 0.0001)	0.051(0.010; 0.0001)	0.045(0.004; 0.0001)	0.040(0.001; 0.0001)
VGGish: pool1	0.017(0.005; 0.0002)	0.014(0.004; 0.0005)	0.033(0.011; 0.0001)	0.008(0.002; 0.0140)	0.004(0.002; 0.2100)	0.005(0.007; 0.0714)
VGGish: pool2	0.032(0.003; 0.0001)	0.030(0.006; 0.0001)	0.054(0.011; 0.0001)	0.031(0.004; 0.0001)	0.019(0.004; 0.0001)	0.025(0.011; 0.0001)
VGGish: pool3	0.059(0.005; 0.0001)	0.068(0.008; 0.0001)	0.086(0.024; 0.0001)	0.099(0.009; 0.0001)	0.075(0.008; 0.0001)	0.075(0.019; 0.0001)
VGGish: pool4	0.063(0.014; 0.0001)	0.066(0.005; 0.0001)	0.084(0.011; 0.0001)	0.103(0.014; 0.0001)	0.084(0.011; 0.0001)	0.072(0.017; 0.0001)
VGGish: fc1_1	0.042(0.013; 0.0001)	0.060(0.008; 0.0001)	0.075(0.013; 0.0001)	0.114(0.011; 0.0001)	0.101(0.013; 0.0001)	0.087(0.024; 0.0001)
VGGish: fc1_2	0.024(0.003; 0.0001)	0.064(0.028; 0.0001)	0.083(0.024; 0.0001)	0.253(0.014; 0.0001)	0.252(0.018; 0.0001)	0.205(0.031; 0.0001)
VGGish: fc2	0.011(0.005; 0.0025)	0.034(0.014; 0.0001)	0.038(0.022; 0.0001)	0.128(0.011; 0.0001)	0.114(0.008; 0.0001)	0.094(0.018; 0.0001)
Yamnet: relu01	0.004(0.001; 0.1863)	0.002(0.004; 0.6344)	0.009(0.003; 0.0074)	-0.002(0.005; 1.0000)	0.000(0.001; 0.9999)	-0.004(0.004; 1.0000)
Yamnet: relu02	0.013(0.004; 0.0006)	0.011(0.002; 0.0018)	0.031(0.008; 0.0001)	0.009(0.002; 0.0078)	0.005(0.001; 0.0769)	0.007(0.003; 0.0314)
Yamnet: relu03	0.028(0.002; 0.0001)	0.028(0.004; 0.0001)	0.060(0.013; 0.0001)	0.036(0.011; 0.0001)	0.021(0.004; 0.0001)	0.023(0.010; 0.0001)
Yamnet: relu04	0.036(0.003; 0.0001)	0.042(0.005; 0.0001)	0.069(0.014; 0.0001)	0.073(0.009; 0.0001)	0.054(0.008; 0.0001)	0.054(0.015; 0.0001)
Yamnet: relu05	0.047(0.003; 0.0001)	0.057(0.006; 0.0001)	0.082(0.017; 0.0001)	0.089(0.015; 0.0001)	0.064(0.007; 0.0001)	0.063(0.017; 0.0001)
Yamnet: relu06	0.058(0.008; 0.0001)	0.071(0.008; 0.0001)	0.094(0.026; 0.0001)	0.118(0.016; 0.0001)	0.091(0.008; 0.0001)	0.081(0.016; 0.0001)
Yamnet: relu07	0.070(0.009; 0.0001)	0.079(0.012; 0.0001)	0.110(0.026; 0.0001)	0.136(0.020; 0.0001)	0.106(0.010; 0.0001)	0.093(0.016; 0.0001)
Yamnet: relu08	0.082(0.011; 0.0001)	0.093(0.014; 0.0001)	0.127(0.026; 0.0001)	0.172(0.024; 0.0001)	0.137(0.012; 0.0001)	0.114(0.019; 0.0001)
Yamnet: relu09	0.073(0.011; 0.0001)	0.085(0.013; 0.0001)	0.125(0.022; 0.0001)	0.163(0.024; 0.0001)	0.133(0.012; 0.0001)	0.107(0.019; 0.0001)
Yamnet: relu10	0.054(0.009; 0.0001)	0.066(0.011; 0.0001)	0.114(0.022; 0.0001)	0.137(0.023; 0.0001)	0.113(0.013; 0.0001)	0.090(0.020; 0.0001)
Yamnet: relu11	0.040(0.007; 0.0001)	0.061(0.012; 0.0001)	0.088(0.021; 0.0001)	0.144(0.029; 0.0001)	0.124(0.012; 0.0001)	0.096(0.018; 0.0001)
Yamnet: relu12	0.017(0.019; 0.0002)	0.026(0.006; 0.0001)	0.025(0.007; 0.0001)	0.045(0.017; 0.0001)	0.028(0.010; 0.0001)	0.014(0.011; 0.0005)
Yamnet: relu13	0.028(0.004; 0.0001)	0.036(0.013; 0.0001)	0.052(0.014; 0.0001)	0.085(0.016; 0.0001)	0.074(0.019; 0.0001)	0.065(0.020; 0.0001)
Yamnet: relu14	0.022(0.009; 0.0001)	0.033(0.005; 0.0001)	0.055(0.011; 0.0001)	0.093(0.009; 0.0001)	0.081(0.012; 0.0001)	0.072(0.010; 0.0001)

Supplementary Table 9: **Layer-cumulative analysis of DNN representation in fMRI data (speech stimuli included).** For each layer-based model, we show the median R^2_{CV} value across CV folds for a particular layer (considered together with the preceding layers), in parentheses, by the interquartile-range of R^2_{CV} across folds, and by the permutation-based p-value for the effect or contrast. Multiple-comparison corrections (FWER = 0.05) applied between ROIs and layers from the same DNN. All statistical tests are one-sided. N fMRI participants = 5.

	fMRI: HG	fMRI: PT	fMRI: PP	fMRI: mSTG	fMRI: pSTG	fMRI: aSTG
Kell: pool1	0.029(0.018; 0.0001)	0.016(0.014; 0.0002)	0.022(0.015; 0.0001)	0.027(0.016; 0.0001)	0.028(0.008; 0.0001)	0.016(0.011; 0.0003)
Kell: pool2	0.042(0.009; 0.0001)	0.049(0.011; 0.0001)	0.051(0.028; 0.0001)	0.107(0.027; 0.0001)	0.100(0.006; 0.0001)	0.071(0.013; 0.0001)
Kell: conv3	0.030(0.022; 0.0001)	0.043(0.015; 0.0001)	0.051(0.024; 0.0001)	0.087(0.033; 0.0001)	0.094(0.012; 0.0001)	0.063(0.015; 0.0001)
Kell: conv4_W	0.030(0.021; 0.0001)	0.048(0.023; 0.0001)	0.047(0.022; 0.0001)	0.108(0.027; 0.0001)	0.126(0.010; 0.0001)	0.089(0.013; 0.0001)
Kell: pool5_flat_W	0.028(0.021; 0.0001)	0.046(0.023; 0.0001)	0.046(0.024; 0.0001)	0.107(0.029; 0.0001)	0.126(0.012; 0.0001)	0.088(0.013; 0.0001)
Kell: fc6_W	0.024(0.029; 0.0001)	0.042(0.028; 0.0001)	0.037(0.023; 0.0001)	0.109(0.032; 0.0001)	0.126(0.012; 0.0001)	0.086(0.014; 0.0001)
Kell: conv4_G	0.030(0.029; 0.0001)	0.040(0.029; 0.0001)	0.032(0.026; 0.0001)	0.125(0.028; 0.0001)	0.143(0.015; 0.0001)	0.097(0.018; 0.0001)
Kell: pool5_flat_G	0.035(0.030; 0.0001)	0.043(0.030; 0.0001)	0.037(0.028; 0.0001)	0.131(0.026; 0.0001)	0.151(0.013; 0.0001)	0.101(0.020; 0.0001)
Kell: fc6_G	0.036(0.024; 0.0001)	0.043(0.028; 0.0001)	0.043(0.027; 0.0001)	0.146(0.030; 0.0001)	0.165(0.016; 0.0001)	0.120(0.021; 0.0001)
VGGish: pool1	0.017(0.005; 0.0002)	0.014(0.004; 0.0010)	0.033(0.011; 0.0001)	0.008(0.002; 0.0321)	0.004(0.002; 0.2803)	0.005(0.007; 0.1290)
VGGish: pool2	0.031(0.005; 0.0001)	0.033(0.001; 0.0001)	0.056(0.008; 0.0001)	0.042(0.007; 0.0001)	0.028(0.004; 0.0001)	0.028(0.006; 0.0001)
VGGish: pool3	0.055(0.008; 0.0001)	0.070(0.010; 0.0001)	0.083(0.026; 0.0001)	0.111(0.009; 0.0001)	0.092(0.012; 0.0001)	0.077(0.017; 0.0001)
VGGish: pool4	0.062(0.006; 0.0001)	0.074(0.008; 0.0001)	0.091(0.021; 0.0001)	0.120(0.014; 0.0001)	0.099(0.016; 0.0001)	0.081(0.016; 0.0001)
VGGish: fc1_1	0.061(0.008; 0.0001)	0.079(0.009; 0.0001)	0.098(0.023; 0.0001)	0.143(0.014; 0.0001)	0.121(0.015; 0.0001)	0.103(0.022; 0.0001)
VGGish: fc1_2	0.059(0.006; 0.0001)	0.086(0.032; 0.0001)	0.102(0.027; 0.0001)	0.272(0.020; 0.0001)	0.277(0.015; 0.0001)	0.210(0.020; 0.0001)
VGGish: fc2	0.057(0.005; 0.0001)	0.086(0.031; 0.0001)	0.102(0.026; 0.0001)	0.276(0.019; 0.0001)	0.285(0.014; 0.0001)	0.216(0.021; 0.0001)
Yamnet: relu01	0.004(0.001; 0.2586)	0.002(0.004; 0.5825)	0.009(0.003; 0.0158)	-0.002(0.005; 0.9997)	0.000(0.001; 0.9524)	-0.004(0.004; 1.0000)
Yamnet: relu02	0.014(0.002; 0.0010)	0.013(0.003; 0.0016)	0.037(0.005; 0.0001)	0.023(0.008; 0.0001)	0.018(0.003; 0.0001)	0.011(0.005; 0.0052)
Yamnet: relu03	0.033(0.004; 0.0001)	0.033(0.004; 0.0001)	0.068(0.014; 0.0001)	0.048(0.012; 0.0001)	0.031(0.004; 0.0001)	0.021(0.006; 0.0001)
Yamnet: relu04	0.036(0.005; 0.0001)	0.046(0.008; 0.0001)	0.066(0.019; 0.0001)	0.100(0.016; 0.0001)	0.092(0.008; 0.0001)	0.069(0.006; 0.0001)
Yamnet: relu05	0.047(0.004; 0.0001)	0.063(0.006; 0.0001)	0.073(0.019; 0.0001)	0.119(0.020; 0.0001)	0.112(0.011; 0.0001)	0.084(0.009; 0.0001)
Yamnet: relu06	0.057(0.006; 0.0001)	0.073(0.010; 0.0001)	0.085(0.023; 0.0001)	0.146(0.024; 0.0001)	0.141(0.012; 0.0001)	0.099(0.008; 0.0001)
Yamnet: relu07	0.068(0.008; 0.0001)	0.082(0.009; 0.0001)	0.099(0.023; 0.0001)	0.172(0.025; 0.0001)	0.164(0.014; 0.0001)	0.122(0.012; 0.0001)
Yamnet: relu08	0.077(0.007; 0.0001)	0.095(0.011; 0.0001)	0.118(0.029; 0.0001)	0.210(0.024; 0.0001)	0.196(0.015; 0.0001)	0.144(0.019; 0.0001)
Yamnet: relu09	0.076(0.009; 0.0001)	0.094(0.011; 0.0001)	0.119(0.041; 0.0001)	0.210(0.024; 0.0001)	0.197(0.015; 0.0001)	0.144(0.018; 0.0001)
Yamnet: relu10	0.077(0.007; 0.0001)	0.092(0.012; 0.0001)	0.115(0.060; 0.0001)	0.210(0.029; 0.0001)	0.198(0.015; 0.0001)	0.143(0.017; 0.0001)
Yamnet: relu11	0.077(0.005; 0.0001)	0.095(0.012; 0.0001)	0.107(0.059; 0.0001)	0.224(0.030; 0.0001)	0.216(0.023; 0.0001)	0.154(0.022; 0.0001)
Yamnet: relu12	0.064(0.016; 0.0001)	0.092(0.025; 0.0001)	0.104(0.057; 0.0001)	0.224(0.038; 0.0001)	0.218(0.017; 0.0001)	0.147(0.018; 0.0001)
Yamnet: relu13	0.065(0.015; 0.0001)	0.089(0.029; 0.0001)	0.102(0.057; 0.0001)	0.226(0.040; 0.0001)	0.222(0.018; 0.0001)	0.152(0.016; 0.0001)
Yamnet: relu14	0.064(0.016; 0.0001)	0.088(0.032; 0.0001)	0.102(0.057; 0.0001)	0.229(0.041; 0.0001)	0.224(0.018; 0.0001)	0.155(0.015; 0.0001)

Supplementary Table 10: Analysis of the increase in fMRI-predictive contribution as each DNN layer is added to the previous layer representations (speech stimuli included). For each layer-based model, we show the median value of the R^2_{CV} increase across CV folds for a particular layer (considered together with the preceding layers and contrasted with the cumulative-layer R^2_{CV} statistic for the preceding layer) followed, in parentheses, by the interquartile-range of R^2_{CV} across folds, and by the permutation-based p-value for the effect or contrast. Multiple-comparison corrections (FWER = 0.05) applied between ROIs and layers from the same DNN. All statistical tests are one-sided. N fMRI participants = 5.

	fMRI: HG	fMRI: PT	fMRI: PP	fMRI: mSTG	fMRI: pSTG	fMRI: aSTG
Kell: pool2	0.015(0.005; 0.0003)	0.035(0.003; 0.0001)	0.028(0.007; 0.0001)	0.078(0.007; 0.0001)	0.073(0.003; 0.0001)	0.059(0.005; 0.0001)
Kell: conv3	0.002(0.021; 0.9211)	0.000(0.014; 1.0000)	0.011(0.023; 0.0023)	-0.006(0.014; 1.0000)	-0.004(0.001; 1.0000)	-0.007(0.007; 1.0000)
Kell: conv4_W	-0.001(0.003; 1.0000)	0.003(0.007; 0.4964)	-0.003(0.004; 1.0000)	0.024(0.005; 0.0001)	0.031(0.004; 0.0001)	0.026(0.012; 0.0001)
Kell: pool5_flat_W	-0.002(0.001; 1.0000)	-0.002(0.001; 1.0000)	-0.000(0.001; 1.0000)	-0.001(0.002; 1.0000)	0.000(0.001; 1.0000)	0.000(0.001; 1.0000)
Kell: fc6_W	-0.006(0.009; 1.0000)	-0.009(0.007; 1.0000)	-0.003(0.008; 1.0000)	0.001(0.002; 0.9680)	0.001(0.001; 1.0000)	-0.001(0.002; 1.0000)
Kell: conv4_G	0.005(0.004; 0.1412)	-0.003(0.003; 1.0000)	-0.002(0.003; 1.0000)	0.014(0.005; 0.0003)	0.018(0.005; 0.0001)	0.013(0.015; 0.0006)
Kell: pool5_flat_G	0.006(0.004; 0.0770)	0.003(0.001; 0.5655)	0.003(0.004; 0.4379)	0.005(0.001; 0.0927)	0.007(0.002; 0.0308)	0.005(0.003; 0.1035)
Kell: fc6_G	0.000(0.003; 1.0000)	0.002(0.003; 0.6764)	0.006(0.002; 0.0770)	0.015(0.002; 0.0003)	0.013(0.003; 0.0006)	0.019(0.002; 0.0001)
VGGish: pool2	0.014(0.003; 0.0003)	0.018(0.003; 0.0001)	0.022(0.003; 0.0001)	0.036(0.010; 0.0001)	0.025(0.003; 0.0001)	0.024(0.002; 0.0001)
VGGish: pool3	0.025(0.005; 0.0001)	0.037(0.006; 0.0001)	0.028(0.011; 0.0001)	0.069(0.007; 0.0001)	0.065(0.011; 0.0001)	0.047(0.010; 0.0001)
VGGish: pool4	0.009(0.009; 0.0113)	0.005(0.002; 0.1023)	0.009(0.005; 0.0132)	0.009(0.003; 0.0113)	0.008(0.003; 0.0183)	0.004(0.001; 0.3368)
VGGish: fc1_1	-0.000(0.001; 1.0000)	0.006(0.004; 0.0876)	0.006(0.004; 0.0678)	0.023(0.005; 0.0001)	0.022(0.005; 0.0001)	0.020(0.006; 0.0001)
VGGish: fc1_2	-0.002(0.004; 1.0000)	0.008(0.022; 0.0153)	0.005(0.026; 0.0958)	0.141(0.018; 0.0001)	0.158(0.015; 0.0001)	0.112(0.045; 0.0001)
VGGish: fc2	-0.002(0.002; 1.0000)	0.000(0.001; 1.0000)	0.001(0.001; 0.9954)	0.004(0.002; 0.3280)	0.008(0.002; 0.0200)	0.006(0.002; 0.0548)
Yamnet: relu02	0.010(0.002; 0.0066)	0.011(0.003; 0.0013)	0.029(0.006; 0.0001)	0.027(0.005; 0.0001)	0.019(0.003; 0.0001)	0.016(0.002; 0.0003)
Yamnet: relu03	0.020(0.006; 0.0001)	0.018(0.001; 0.0001)	0.031(0.008; 0.0001)	0.025(0.009; 0.0001)	0.014(0.002; 0.0003)	0.011(0.009; 0.0023)
Yamnet: relu04	0.005(0.004; 0.1310)	0.017(0.005; 0.0003)	0.000(0.008; 1.0000)	0.054(0.005; 0.0001)	0.061(0.006; 0.0001)	0.049(0.005; 0.0001)
Yamnet: relu05	0.011(0.003; 0.0031)	0.015(0.005; 0.0003)	0.009(0.005; 0.0109)	0.023(0.004; 0.0001)	0.020(0.004; 0.0001)	0.015(0.003; 0.0003)
Yamnet: relu06	0.011(0.004; 0.0043)	0.010(0.002; 0.0060)	0.012(0.007; 0.0011)	0.027(0.005; 0.0001)	0.028(0.003; 0.0001)	0.015(0.002; 0.0003)
Yamnet: relu07	0.011(0.006; 0.0019)	0.009(0.003; 0.0131)	0.015(0.004; 0.0003)	0.028(0.003; 0.0001)	0.025(0.004; 0.0001)	0.022(0.003; 0.0001)
Yamnet: relu08	0.009(0.004; 0.0132)	0.014(0.002; 0.0003)	0.021(0.007; 0.0001)	0.040(0.006; 0.0001)	0.032(0.003; 0.0001)	0.022(0.005; 0.0001)
Yamnet: relu09	0.000(0.002; 1.0000)	-0.001(0.001; 1.0000)	-0.001(0.004; 1.0000)	-0.001(0.001; 1.0000)	0.001(0.001; 0.9969)	-0.001(0.002; 1.0000)
Yamnet: relu10	0.002(0.002; 0.9471)	0.001(0.002; 1.0000)	-0.005(0.018; 1.0000)	0.001(0.002; 0.9999)	0.001(0.002; 0.9999)	-0.001(0.002; 1.0000)
Yamnet: relu11	-0.001(0.001; 1.0000)	0.003(0.002; 0.5487)	-0.002(0.012; 1.0000)	0.016(0.006; 0.0003)	0.017(0.004; 0.0003)	0.010(0.002; 0.0045)
Yamnet: relu12	-0.008(0.019; 1.0000)	-0.005(0.015; 1.0000)	-0.005(0.008; 1.0000)	0.001(0.006; 0.9752)	0.004(0.005; 0.2513)	-0.004(0.010; 1.0000)
Yamnet: relu13	-0.001(0.002; 1.0000)	-0.003(0.004; 1.0000)	-0.002(0.003; 1.0000)	0.002(0.001; 0.9048)	0.005(0.002; 0.1400)	0.005(0.001; 0.0932)
Yamnet: relu14	-0.000(0.001; 1.0000)	-0.001(0.002; 1.0000)	-0.001(0.001; 1.0000)	0.003(0.001; 0.4811)	0.003(0.000; 0.5758)	0.004(0.002; 0.2445)

Supplementary Table 11: **Layer-by-layer analysis of DNN representation in fMRI data (speech stimuli excluded).** For each layer-based model, we show the median R^2_{CV} value across CV folds for a particular layer followed, in parentheses, by the interquartile-range of R^2_{CV} across folds, and by the permutation-based p-value for the effect or contrast. Multiple-comparison corrections (FWER = 0.05) applied between ROIs and layers from the same DNN. All statistical tests are one-sided. N fMRI participants = 5.

	fMRI: HG	fMRI: PT	fMRI: PP	fMRI: mSTG	fMRI: pSTG	fMRI: aSTG
Kell: pool1	0.032(0.017; 0.0001)	0.015(0.012; 0.0009)	0.018(0.008; 0.0004)	0.005(0.024; 0.1086)	0.015(0.008; 0.0009)	-0.003(0.017; 1.0000)
Kell: pool2	0.047(0.009; 0.0001)	0.027(0.011; 0.0001)	0.032(0.020; 0.0001)	0.036(0.039; 0.0001)	0.040(0.010; 0.0001)	0.017(0.015; 0.0004)
Kell: conv3	0.024(0.019; 0.0001)	0.013(0.016; 0.0022)	0.003(0.016; 0.3659)	0.018(0.037; 0.0003)	0.033(0.008; 0.0001)	0.006(0.021; 0.0597)
Kell: conv4_W	0.017(0.026; 0.0004)	0.011(0.022; 0.0063)	-0.004(0.013; 1.0000)	0.010(0.040; 0.0092)	0.028(0.007; 0.0001)	0.003(0.016; 0.3545)
Kell: pool5_flat_W	0.022(0.016; 0.0002)	0.014(0.015; 0.0013)	0.003(0.009; 0.4062)	0.015(0.027; 0.0009)	0.021(0.003; 0.0002)	0.006(0.012; 0.0663)
Kell: fc6_W	0.004(0.017; 0.2433)	-0.003(0.010; 1.0000)	-0.002(0.012; 1.0000)	-0.003(0.008; 1.0000)	-0.001(0.002; 1.0000)	-0.001(0.003; 1.0000)
Kell: conv4_G	0.021(0.025; 0.0002)	0.010(0.019; 0.0074)	-0.002(0.011; 1.0000)	0.010(0.033; 0.0096)	0.026(0.008; 0.0001)	0.003(0.013; 0.4457)
Kell: pool5_flat_G	0.041(0.013; 0.0001)	0.022(0.013; 0.0002)	0.013(0.006; 0.0020)	0.020(0.020; 0.0002)	0.027(0.005; 0.0001)	0.013(0.006; 0.0025)
Kell: fc6_G	0.033(0.006; 0.0001)	0.019(0.004; 0.0002)	0.024(0.006; 0.0001)	0.036(0.009; 0.0001)	0.029(0.004; 0.0001)	0.027(0.002; 0.0001)
VGGish: pool1	0.021(0.004; 0.0002)	0.015(0.006; 0.0008)	0.036(0.013; 0.0001)	0.009(0.006; 0.0180)	0.004(0.004; 0.2601)	0.001(0.009; 0.9217)
VGGish: pool2	0.037(0.004; 0.0001)	0.029(0.008; 0.0001)	0.059(0.014; 0.0001)	0.031(0.004; 0.0001)	0.017(0.007; 0.0004)	0.018(0.011; 0.0004)
VGGish: pool3	0.058(0.006; 0.0001)	0.048(0.009; 0.0001)	0.071(0.012; 0.0001)	0.060(0.013; 0.0001)	0.036(0.012; 0.0001)	0.032(0.015; 0.0001)
VGGish: pool4	0.058(0.015; 0.0001)	0.047(0.008; 0.0001)	0.070(0.008; 0.0001)	0.081(0.014; 0.0001)	0.057(0.012; 0.0001)	0.051(0.013; 0.0001)
VGGish: fc1_1	0.042(0.017; 0.0001)	0.039(0.005; 0.0001)	0.059(0.013; 0.0001)	0.068(0.005; 0.0001)	0.048(0.021; 0.0001)	0.044(0.020; 0.0001)
VGGish: fc1_2	0.018(0.002; 0.0004)	0.025(0.006; 0.0001)	0.047(0.006; 0.0001)	0.121(0.019; 0.0001)	0.108(0.006; 0.0001)	0.080(0.017; 0.0001)
VGGish: fc2	0.007(0.007; 0.0347)	0.014(0.002; 0.0010)	0.020(0.008; 0.0002)	0.058(0.010; 0.0001)	0.039(0.004; 0.0001)	0.033(0.010; 0.0001)
Yamnet: relu01	0.003(0.002; 0.5255)	0.003(0.003; 0.5631)	0.008(0.002; 0.0208)	-0.002(0.007; 1.0000)	0.001(0.002; 0.9964)	-0.001(0.014; 1.0000)
Yamnet: relu02	0.015(0.002; 0.0008)	0.015(0.004; 0.0008)	0.036(0.008; 0.0001)	0.017(0.003; 0.0004)	0.010(0.004; 0.0079)	0.012(0.006; 0.0032)
Yamnet: relu03	0.035(0.005; 0.0001)	0.029(0.008; 0.0001)	0.071(0.016; 0.0001)	0.058(0.013; 0.0001)	0.032(0.010; 0.0001)	0.032(0.011; 0.0001)
Yamnet: relu04	0.044(0.007; 0.0001)	0.038(0.009; 0.0001)	0.072(0.015; 0.0001)	0.065(0.019; 0.0001)	0.039(0.011; 0.0001)	0.034(0.013; 0.0001)
Yamnet: relu05	0.049(0.007; 0.0001)	0.044(0.010; 0.0001)	0.074(0.012; 0.0001)	0.072(0.016; 0.0001)	0.046(0.012; 0.0001)	0.039(0.014; 0.0001)
Yamnet: relu06	0.057(0.010; 0.0001)	0.052(0.010; 0.0001)	0.081(0.017; 0.0001)	0.090(0.019; 0.0001)	0.060(0.013; 0.0001)	0.046(0.010; 0.0001)
Yamnet: relu07	0.067(0.009; 0.0001)	0.059(0.009; 0.0001)	0.093(0.017; 0.0001)	0.110(0.022; 0.0001)	0.077(0.014; 0.0001)	0.063(0.010; 0.0001)
Yamnet: relu08	0.076(0.008; 0.0001)	0.066(0.010; 0.0001)	0.104(0.013; 0.0001)	0.135(0.029; 0.0001)	0.095(0.020; 0.0001)	0.077(0.014; 0.0001)
Yamnet: relu09	0.069(0.007; 0.0001)	0.061(0.008; 0.0001)	0.104(0.011; 0.0001)	0.141(0.030; 0.0001)	0.102(0.020; 0.0001)	0.083(0.014; 0.0001)
Yamnet: relu10	0.051(0.008; 0.0001)	0.047(0.008; 0.0001)	0.100(0.022; 0.0001)	0.132(0.028; 0.0001)	0.095(0.023; 0.0001)	0.083(0.016; 0.0001)
Yamnet: relu11	0.033(0.008; 0.0001)	0.036(0.009; 0.0001)	0.066(0.023; 0.0001)	0.120(0.034; 0.0001)	0.091(0.019; 0.0001)	0.067(0.018; 0.0001)
Yamnet: relu12	0.011(0.021; 0.0051)	0.021(0.006; 0.0002)	0.029(0.019; 0.0001)	0.060(0.018; 0.0001)	0.037(0.023; 0.0001)	0.025(0.017; 0.0001)
Yamnet: relu13	0.017(0.009; 0.0004)	0.019(0.007; 0.0002)	0.044(0.013; 0.0001)	0.050(0.019; 0.0001)	0.039(0.033; 0.0001)	0.034(0.022; 0.0001)
Yamnet: relu14	0.015(0.012; 0.0008)	0.015(0.006; 0.0008)	0.035(0.020; 0.0001)	0.049(0.010; 0.0001)	0.038(0.018; 0.0001)	0.035(0.009; 0.0001)

Supplementary Table 12: **Layer-cumulative analysis of DNN representation in fMRI data (speech stimuli excluded).** For each layer-based model, we show the median R^2_{CV} value across CV folds for a particular layer (considered together with the preceding layers), in parentheses, by the interquartile-range of R^2_{CV} across folds, and by the permutation-based p-value for the effect or contrast. Multiple-comparison corrections (FWER = 0.05) applied between ROIs and layers from the same DNN. All statistical tests are one-sided. N fMRI participants = 5.

	fMRI: HG	fMRI: PT	fMRI: PP	fMRI: mSTG	fMRI: pSTG	fMRI: aSTG
Kell: pool1	0.032(0.017; 0.0001)	0.015(0.012; 0.0021)	0.018(0.008; 0.0008)	0.005(0.024; 0.1644)	0.015(0.008; 0.0023)	-0.003(0.017; 1.0000)
Kell: pool2	0.041(0.010; 0.0001)	0.029(0.007; 0.0001)	0.031(0.021; 0.0001)	0.044(0.036; 0.0001)	0.045(0.009; 0.0001)	0.019(0.014; 0.0005)
Kell: conv3	0.026(0.029; 0.0001)	0.017(0.020; 0.0008)	0.036(0.024; 0.0001)	0.021(0.050; 0.0001)	0.036(0.016; 0.0001)	0.007(0.015; 0.0917)
Kell: conv4_W	0.025(0.027; 0.0001)	0.019(0.017; 0.0005)	0.035(0.030; 0.0001)	0.019(0.051; 0.0005)	0.035(0.016; 0.0001)	0.005(0.017; 0.1980)
Kell: pool5_flat_W	0.022(0.026; 0.0001)	0.017(0.019; 0.0010)	0.035(0.031; 0.0001)	0.018(0.054; 0.0006)	0.035(0.016; 0.0001)	0.003(0.013; 0.4321)
Kell: fc6_W	0.015(0.043; 0.0021)	0.009(0.025; 0.0433)	0.032(0.022; 0.0001)	0.020(0.055; 0.0003)	0.042(0.020; 0.0001)	0.006(0.010; 0.1495)
Kell: conv4_G	0.021(0.040; 0.0002)	0.009(0.026; 0.0342)	0.031(0.024; 0.0001)	0.017(0.055; 0.0009)	0.041(0.019; 0.0001)	0.000(0.011; 0.9499)
Kell: pool5_flat_G	0.026(0.040; 0.0001)	0.010(0.027; 0.0233)	0.032(0.023; 0.0001)	0.017(0.054; 0.0010)	0.039(0.020; 0.0001)	-0.002(0.011; 0.9993)
Kell: fc6_G	0.026(0.034; 0.0001)	0.007(0.022; 0.0931)	0.034(0.024; 0.0001)	0.021(0.054; 0.0001)	0.041(0.019; 0.0001)	0.007(0.010; 0.0858)
VGGish: pool1	0.021(0.004; 0.0001)	0.015(0.006; 0.0018)	0.036(0.013; 0.0001)	0.009(0.006; 0.0406)	0.004(0.004; 0.3153)	0.001(0.009; 0.7751)
VGGish: pool2	0.036(0.004; 0.0001)	0.029(0.007; 0.0001)	0.060(0.011; 0.0001)	0.042(0.008; 0.0001)	0.025(0.006; 0.0001)	0.019(0.012; 0.0005)
VGGish: pool3	0.053(0.007; 0.0001)	0.044(0.009; 0.0001)	0.070(0.012; 0.0001)	0.063(0.012; 0.0001)	0.041(0.010; 0.0001)	0.028(0.015; 0.0001)
VGGish: pool4	0.056(0.011; 0.0001)	0.047(0.011; 0.0001)	0.079(0.011; 0.0001)	0.080(0.015; 0.0001)	0.051(0.015; 0.0001)	0.042(0.014; 0.0001)
VGGish: fc1_1	0.053(0.013; 0.0001)	0.049(0.006; 0.0001)	0.080(0.015; 0.0001)	0.085(0.011; 0.0001)	0.053(0.021; 0.0001)	0.045(0.023; 0.0001)
VGGish: fc1_2	0.051(0.015; 0.0001)	0.042(0.015; 0.0001)	0.076(0.021; 0.0001)	0.123(0.040; 0.0001)	0.114(0.017; 0.0001)	0.055(0.026; 0.0001)
VGGish: fc2	0.049(0.012; 0.0001)	0.042(0.016; 0.0001)	0.076(0.020; 0.0001)	0.125(0.041; 0.0001)	0.121(0.018; 0.0001)	0.060(0.026; 0.0001)
Yamnet: relu01	0.003(0.002; 0.5047)	0.003(0.003; 0.5286)	0.008(0.002; 0.0448)	-0.002(0.007; 0.9995)	0.001(0.002; 0.8910)	-0.001(0.014; 0.9980)
Yamnet: relu02	0.020(0.003; 0.0002)	0.015(0.009; 0.0023)	0.044(0.009; 0.0001)	0.033(0.013; 0.0001)	0.026(0.005; 0.0001)	0.014(0.015; 0.0026)
Yamnet: relu03	0.036(0.007; 0.0001)	0.029(0.009; 0.0001)	0.077(0.015; 0.0001)	0.065(0.020; 0.0001)	0.043(0.009; 0.0001)	0.021(0.013; 0.0001)
Yamnet: relu04	0.040(0.004; 0.0001)	0.035(0.009; 0.0001)	0.077(0.014; 0.0001)	0.072(0.020; 0.0001)	0.052(0.010; 0.0001)	0.024(0.013; 0.0001)
Yamnet: relu05	0.044(0.004; 0.0001)	0.041(0.007; 0.0001)	0.076(0.013; 0.0001)	0.076(0.019; 0.0001)	0.057(0.012; 0.0001)	0.028(0.009; 0.0001)
Yamnet: relu06	0.052(0.007; 0.0001)	0.047(0.006; 0.0001)	0.085(0.018; 0.0001)	0.093(0.019; 0.0001)	0.075(0.011; 0.0001)	0.033(0.013; 0.0001)
Yamnet: relu07	0.064(0.005; 0.0001)	0.054(0.007; 0.0001)	0.095(0.016; 0.0001)	0.111(0.022; 0.0001)	0.090(0.010; 0.0001)	0.054(0.015; 0.0001)
Yamnet: relu08	0.071(0.007; 0.0001)	0.059(0.009; 0.0001)	0.104(0.023; 0.0001)	0.131(0.024; 0.0001)	0.105(0.010; 0.0001)	0.060(0.018; 0.0001)
Yamnet: relu09	0.071(0.007; 0.0001)	0.058(0.008; 0.0001)	0.106(0.021; 0.0001)	0.136(0.025; 0.0001)	0.111(0.013; 0.0001)	0.065(0.024; 0.0001)
Yamnet: relu10	0.070(0.007; 0.0001)	0.057(0.008; 0.0001)	0.103(0.045; 0.0001)	0.136(0.034; 0.0001)	0.111(0.015; 0.0001)	0.067(0.018; 0.0001)
Yamnet: relu11	0.069(0.007; 0.0001)	0.058(0.006; 0.0001)	0.095(0.045; 0.0001)	0.141(0.036; 0.0001)	0.120(0.019; 0.0001)	0.066(0.024; 0.0001)
Yamnet: relu12	0.051(0.020; 0.0001)	0.049(0.021; 0.0001)	0.091(0.049; 0.0001)	0.141(0.051; 0.0001)	0.118(0.024; 0.0001)	0.055(0.025; 0.0001)
Yamnet: relu13	0.050(0.022; 0.0001)	0.044(0.027; 0.0001)	0.092(0.049; 0.0001)	0.137(0.054; 0.0001)	0.118(0.033; 0.0001)	0.054(0.028; 0.0001)
Yamnet: relu14	0.049(0.021; 0.0001)	0.042(0.025; 0.0001)	0.089(0.047; 0.0001)	0.136(0.054; 0.0001)	0.117(0.033; 0.0001)	0.053(0.028; 0.0001)

Supplementary Table 13: Analysis of the increase in fMRI-predictive contribution as each DNN layer is added to the previous layer representations (speech stimuli excluded). For each layer-based model, we show the median value of the R^2_{CV} increase across CV folds for a particular layer (considered together with the preceding layers and contrasted with the cumulative-layer R^2_{CV} statistic for the preceding layer) followed, in parentheses, by the interquartile-range of R^2_{CV} across folds, and by the permutation-based p-value for the effect or contrast. Multiple-comparison corrections (FWER = 0.05) applied between ROIs and layers from the same DNN. All statistical tests are one-sided. N fMRI participants = 5.

	fMRI: HG	fMRI: PT	fMRI: PP	fMRI: mSTG	fMRI: pSTG	fMRI: aSTG
Kell: pool2	0.011(0.007; 0.0073)	0.015(0.002; 0.0005)	0.013(0.004; 0.0015)	0.038(0.005; 0.0001)	0.032(0.002; 0.0001)	0.023(0.002; 0.0001)
Kell: conv3	-0.001(0.025; 1.0000)	0.001(0.022; 1.0000)	0.016(0.021; 0.0005)	-0.009(0.021; 1.0000)	-0.007(0.007; 1.0000)	-0.010(0.009; 1.0000)
Kell: conv4_W	-0.002(0.008; 1.0000)	-0.000(0.009; 1.0000)	-0.002(0.005; 1.0000)	0.000(0.002; 1.0000)	-0.001(0.001; 1.0000)	-0.002(0.002; 1.0000)
Kell: pool5_flat_W	-0.002(0.001; 1.0000)	-0.002(0.002; 1.0000)	-0.001(0.001; 1.0000)	-0.001(0.002; 1.0000)	0.000(0.002; 1.0000)	-0.002(0.004; 1.0000)
Kell: fc6_W	-0.007(0.014; 1.0000)	-0.011(0.009; 1.0000)	-0.002(0.013; 1.0000)	0.002(0.004; 0.8935)	0.009(0.006; 0.0216)	0.004(0.002; 0.5051)
Kell: conv4_G	0.005(0.002; 0.1890)	-0.001(0.002; 1.0000)	-0.001(0.001; 1.0000)	-0.002(0.001; 1.0000)	-0.001(0.002; 1.0000)	-0.003(0.006; 1.0000)
Kell: pool5_flat_G	0.005(0.002; 0.1677)	0.001(0.001; 1.0000)	0.001(0.002; 1.0000)	-0.001(0.002; 1.0000)	-0.001(0.002; 1.0000)	-0.003(0.003; 1.0000)
Kell: fc6_G	-0.001(0.003; 1.0000)	-0.000(0.005; 1.0000)	0.003(0.003; 0.6405)	0.005(0.001; 0.1628)	0.002(0.002; 0.9782)	0.007(0.002; 0.0461)
VGGish: pool2	0.015(0.002; 0.0005)	0.014(0.001; 0.0010)	0.024(0.004; 0.0001)	0.036(0.007; 0.0001)	0.024(0.003; 0.0001)	0.019(0.001; 0.0002)
VGGish: pool3	0.017(0.003; 0.0004)	0.017(0.005; 0.0003)	0.013(0.004; 0.0017)	0.019(0.004; 0.0002)	0.014(0.008; 0.0013)	0.008(0.003; 0.0358)
VGGish: pool4	0.005(0.013; 0.1745)	0.005(0.005; 0.2754)	0.008(0.004; 0.0363)	0.018(0.004; 0.0002)	0.014(0.003; 0.0009)	0.014(0.004; 0.0010)
VGGish: fc1_1	0.000(0.003; 1.0000)	0.002(0.003; 0.9738)	0.003(0.005; 0.7923)	0.004(0.004; 0.2885)	0.003(0.004; 0.6273)	0.002(0.008; 0.9738)
VGGish: fc1_2	-0.003(0.004; 1.0000)	-0.007(0.015; 1.0000)	-0.001(0.014; 1.0000)	0.053(0.026; 0.0001)	0.060(0.010; 0.0001)	0.022(0.034; 0.0001)
VGGish: fc2	-0.002(0.002; 1.0000)	-0.001(0.002; 1.0000)	0.001(0.003; 1.0000)	0.001(0.003; 0.9862)	0.006(0.002; 0.0925)	0.004(0.001; 0.3662)
Yamnet: relu02	0.018(0.005; 0.0002)	0.013(0.005; 0.0019)	0.037(0.005; 0.0001)	0.038(0.010; 0.0001)	0.027(0.003; 0.0001)	0.018(0.005; 0.0002)
Yamnet: relu03	0.018(0.006; 0.0003)	0.015(0.001; 0.0006)	0.033(0.007; 0.0001)	0.034(0.013; 0.0001)	0.016(0.005; 0.0005)	0.013(0.009; 0.0022)
Yamnet: relu04	0.005(0.004; 0.2809)	0.006(0.002; 0.1285)	0.001(0.002; 0.9992)	0.007(0.002; 0.0517)	0.009(0.005; 0.0245)	0.003(0.002; 0.6604)
Yamnet: relu05	0.004(0.002; 0.2940)	0.006(0.002; 0.1398)	0.001(0.001; 0.9998)	0.005(0.002; 0.2553)	0.006(0.001; 0.1351)	0.003(0.003; 0.5484)
Yamnet: relu06	0.008(0.002; 0.0304)	0.007(0.002; 0.0800)	0.010(0.004; 0.0116)	0.018(0.003; 0.0002)	0.018(0.003; 0.0002)	0.005(0.003; 0.1931)
Yamnet: relu07	0.013(0.003; 0.0023)	0.007(0.003; 0.0456)	0.011(0.003; 0.0085)	0.020(0.003; 0.0001)	0.018(0.003; 0.0003)	0.019(0.002; 0.0002)
Yamnet: relu08	0.007(0.005; 0.0653)	0.006(0.003; 0.1405)	0.012(0.010; 0.0028)	0.022(0.005; 0.0001)	0.013(0.004; 0.0015)	0.010(0.004; 0.0117)
Yamnet: relu09	-0.000(0.001; 1.0000)	0.000(0.001; 1.0000)	0.001(0.005; 0.9998)	0.004(0.002; 0.3050)	0.006(0.003; 0.1126)	0.004(0.004; 0.3518)
Yamnet: relu10	0.000(0.002; 1.0000)	-0.000(0.003; 1.0000)	-0.006(0.016; 1.0000)	-0.000(0.003; 1.0000)	-0.001(0.004; 1.0000)	0.001(0.007; 0.9998)
Yamnet: relu11	-0.001(0.001; 1.0000)	0.000(0.002; 1.0000)	-0.003(0.005; 1.0000)	0.007(0.008; 0.0559)	0.007(0.002; 0.0471)	0.001(0.003; 0.9904)
Yamnet: relu12	-0.012(0.023; 1.0000)	-0.007(0.018; 1.0000)	-0.004(0.006; 1.0000)	0.002(0.014; 0.9217)	-0.001(0.002; 1.0000)	-0.008(0.009; 1.0000)
Yamnet: relu13	-0.001(0.002; 1.0000)	-0.004(0.002; 1.0000)	0.001(0.001; 1.0000)	-0.003(0.002; 1.0000)	-0.001(0.002; 1.0000)	-0.003(0.005; 1.0000)
Yamnet: relu14	-0.000(0.003; 1.0000)	-0.001(0.002; 1.0000)	-0.002(0.002; 1.0000)	-0.001(0.001; 1.0000)	-0.000(0.001; 1.0000)	-0.000(0.001; 1.0000)

Supplementary Table 14: **DNN-based prediction of behavioural data from fMRI ROI data (speech fMRI stimuli included).** For each behavioural datasets and fMRI-based model, we show the median R^2_{CV} value across CV folds for a particular model or models contrast followed, in parentheses, by the interquartile-range of R^2_{CV} across folds, and by the permutation-based p-value for the effect or contrast. u = unique behaviour variance predicted by the Heschl's gyrus (HG) and posterior superior temporal gyrus (pSTG) ROIs; c = common predictive variance. Multiple-comparison corrections (FWER = 0.05) applied between ROI-specific models and pairwise contrasts, and between predictive variance components of the HG+pSTG behavioural prediction. All statistical tests are one-sided, with the exception of the contrasts, which are two-sided. N sound or word dissimilarity participants = 20.

	Sound dissimilarity	Word dissimilarity
All ROIs	0.213(0.054; 0.0001)	0.100(0.011; 0.0001)
HG	0.144(0.032; 0.0001)	0.051(0.008; 0.0001)
PT	0.198(0.052; 0.0001)	0.084(0.011; 0.0001)
PP	0.180(0.050; 0.0001)	0.065(0.011; 0.0001)
mSTG	0.149(0.044; 0.0001)	0.067(0.011; 0.0001)
pSTG	0.147(0.045; 0.0001)	0.070(0.011; 0.0001)
aSTG	0.133(0.039; 0.0001)	0.067(0.010; 0.0001)
uHG(pSTG)	0.058(0.017; 0.0001)	0.016(0.007; 0.0013)
upSTG(HG)	0.058(0.024; 0.0001)	0.032(0.009; 0.0001)
cHG-pSTG	0.086(0.021; 0.0001)	0.037(0.006; 0.0001)
HG vs PT	-0.054(0.019; 0.0001)	-0.033(0.008; 0.0002)
HG vs PP	-0.037(0.017; 0.0001)	-0.013(0.006; 0.0191)
HG vs mSTG	-0.003(0.024; 0.2559)	-0.014(0.011; 0.0151)
HG vs pSTG	-0.003(0.026; 0.2389)	-0.017(0.012; 0.0065)
HG vs aSTG	0.012(0.027; 0.0027)	-0.014(0.011; 0.0164)
PT vs PP	0.017(0.007; 0.0005)	0.020(0.003; 0.0032)
PT vs mSTG	0.049(0.011; 0.0001)	0.019(0.005; 0.0036)
PT vs pSTG	0.048(0.013; 0.0001)	0.016(0.005; 0.0108)
PT vs aSTG	0.065(0.014; 0.0001)	0.019(0.006; 0.0046)
PP vs mSTG	0.032(0.015; 0.0001)	-0.001(0.006; 0.9024)
PP vs pSTG	0.032(0.018; 0.0001)	-0.004(0.007; 0.3764)
PP vs aSTG	0.047(0.020; 0.0001)	-0.001(0.008; 0.9092)
mSTG vs pSTG	-0.001(0.004; 0.9570)	-0.003(0.001; 0.5245)
mSTG vs aSTG	0.016(0.007; 0.0005)	-0.000(0.001; 1.0000)
pSTG vs aSTG	0.016(0.006; 0.0006)	0.003(0.002; 0.5908)
uHG(pSTG) vs upSTG(HG)	-0.005(0.029; 0.0615)	-0.015(0.015; 0.0064)

Supplementary Table 15: **DNN-based prediction of behavioural data from fMRI ROI data (speech fMRI stimuli excluded).** For each behavioural datasets and fMRI-based model, we show the median R^2_{CV} value across CV folds for a particular model or models contrast followed, in parentheses, by the interquartile-range of R^2_{CV} across folds, and by the permutation-based p-value for the effect or contrast. u = unique behaviour variance predicted by the Heschl's gyrus (HG) and posterior superior temporal gyrus (pSTG) ROIs; c = common predictive variance. Multiple-comparison corrections (FWER = 0.05) applied between ROI-specific models and pairwise contrasts, and between predictive variance components of the HG+pSTG behavioural prediction. All statistical tests are one-sided, with the exception of the contrasts, which are two-sided. N sound or word dissimilarity participants = 20.

	Sound dissimilarity	Word dissimilarity
All ROIs	0.219(0.044; 0.0001)	0.099(0.011; 0.0001)
HG	0.142(0.030; 0.0001)	0.055(0.010; 0.0001)
PT	0.183(0.037; 0.0001)	0.081(0.011; 0.0001)
PP	0.153(0.035; 0.0001)	0.053(0.011; 0.0001)
mSTG	0.162(0.045; 0.0001)	0.050(0.010; 0.0001)
pSTG	0.167(0.044; 0.0001)	0.062(0.011; 0.0001)
aSTG	0.171(0.042; 0.0001)	0.056(0.012; 0.0001)
uHG(pSTG)	0.038(0.011; 0.0001)	0.014(0.005; 0.0046)
upSTG(HG)	0.058(0.019; 0.0001)	0.023(0.005; 0.0001)
cHG-pSTG	0.107(0.020; 0.0001)	0.041(0.005; 0.0001)
HG vs PT	-0.041(0.017; 0.0001)	-0.026(0.004; 0.0006)
HG vs PP	-0.011(0.013; 0.0019)	0.001(0.003; 0.9396)
HG vs mSTG	-0.017(0.024; 0.0003)	0.003(0.010; 0.7105)
HG vs pSTG	-0.025(0.026; 0.0001)	-0.009(0.011; 0.1183)
HG vs aSTG	-0.028(0.025; 0.0001)	-0.002(0.010; 0.8817)
PT vs PP	0.028(0.007; 0.0001)	0.028(0.003; 0.0003)
PT vs mSTG	0.022(0.012; 0.0001)	0.030(0.007; 0.0003)
PT vs pSTG	0.015(0.015; 0.0004)	0.018(0.008; 0.0070)
PT vs aSTG	0.011(0.016; 0.0024)	0.025(0.007; 0.0010)
PP vs mSTG	-0.007(0.015; 0.0257)	0.001(0.007; 0.9236)
PP vs pSTG	-0.012(0.017; 0.0013)	-0.010(0.008; 0.0849)
PP vs aSTG	-0.017(0.017; 0.0002)	-0.003(0.007; 0.6250)
mSTG vs pSTG	-0.006(0.005; 0.0320)	-0.011(0.002; 0.0505)
mSTG vs aSTG	-0.011(0.006; 0.0021)	-0.005(0.003; 0.3594)
pSTG vs aSTG	-0.004(0.005; 0.1067)	0.007(0.003; 0.1875)
uHG(pSTG) vs upSTG(HG)	-0.020(0.021; 0.0001)	-0.010(0.011; 0.0455)

Supplementary Table 16: **Analysis of the representation of the components of the category model in fMRI data.** For each fMRI ROI and category-model component, we show the median R^2_{CV} value across CV folds for a particular component followed, in parentheses, by the interquartile-range of R^2_{CV} across folds, and by the permutation-based p-value for the effect or contrast. Multiple-comparison corrections (FWER = 0.05) applied between ROIs and category-model components, or between ROIs and between-category contrasts. All statistical tests are one-sided, with the exception of the contrasts, which are two-sided. N fMRI participants = 5.

	fMRI: HG	fMRI: PT	fMRI: PP	fMRI: mSTG	fMRI: pSTG	fMRI: aSTG
Speech	0.011(0.003; 0.0001)	0.012(0.003; 0.0001)	0.017(0.004; 0.0001)	0.020(0.003; 0.0001)	0.019(0.004; 0.0001)	0.013(0.004; 0.0001)
Voice	0.001(0.003; 0.4784)	0.004(0.002; 0.0115)	0.004(0.002; 0.0074)	0.014(0.001; 0.0001)	0.011(0.002; 0.0001)	0.011(0.002; 0.0001)
Animal	-0.002(0.002; 1.0000)	0.001(0.001; 0.7916)	0.002(0.002; 0.0893)	0.014(0.002; 0.0001)	0.012(0.003; 0.0001)	0.012(0.004; 0.0001)
Music	0.001(0.001; 0.3718)	0.005(0.002; 0.0008)	0.004(0.003; 0.0058)	0.017(0.003; 0.0001)	0.017(0.001; 0.0001)	0.015(0.001; 0.0001)
Nature	0.003(0.000; 0.0319)	0.007(0.003; 0.0001)	0.010(0.005; 0.0001)	0.024(0.006; 0.0001)	0.021(0.003; 0.0001)	0.012(0.003; 0.0001)
Tools	0.001(0.001; 0.3662)	0.004(0.002; 0.0137)	0.007(0.004; 0.0001)	0.014(0.003; 0.0001)	0.015(0.003; 0.0001)	0.015(0.003; 0.0001)
Speech vs Voice	0.011(0.003; 0.0001)	0.008(0.005; 0.0003)	0.013(0.004; 0.0001)	0.006(0.004; 0.0065)	0.009(0.002; 0.0001)	0.003(0.002; 0.6963)
Speech vs Animal	0.014(0.003; 0.0001)	0.012(0.004; 0.0001)	0.015(0.004; 0.0001)	0.005(0.004; 0.0273)	0.008(0.005; 0.0003)	0.000(0.009; 1.0000)
Speech vs Music	0.010(0.004; 0.0001)	0.006(0.005; 0.0084)	0.013(0.004; 0.0001)	0.003(0.002; 0.3826)	0.002(0.003; 0.7598)	-0.002(0.004; 0.8761)
Speech vs Nature	0.008(0.003; 0.0005)	0.006(0.008; 0.0129)	0.009(0.004; 0.0001)	-0.004(0.004; 0.1137)	-0.001(0.005; 0.9996)	-0.000(0.008; 1.0000)
Speech vs Tools	0.010(0.004; 0.0001)	0.009(0.005; 0.0001)	0.013(0.004; 0.0001)	0.006(0.002; 0.0037)	0.006(0.004; 0.0097)	-0.004(0.007; 0.2237)
Voice vs Animal	0.002(0.004; 0.9501)	0.003(0.004; 0.5148)	0.002(0.001; 0.9922)	-0.001(0.002; 1.0000)	0.000(0.003; 1.0000)	-0.001(0.007; 1.0000)
Voice vs Music	-0.001(0.001; 1.0000)	-0.002(0.003; 0.8330)	0.000(0.003; 1.0000)	-0.003(0.004; 0.5033)	-0.006(0.002; 0.0107)	-0.004(0.003; 0.1527)
Voice vs Nature	-0.003(0.003; 0.6929)	-0.005(0.005; 0.0521)	-0.006(0.006; 0.0067)	-0.011(0.007; 0.0001)	-0.010(0.004; 0.0001)	-0.001(0.006; 0.9954)
Voice vs Tools	-0.001(0.003; 1.0000)	0.000(0.004; 1.0000)	-0.003(0.005; 0.5303)	0.000(0.003; 1.0000)	-0.004(0.004; 0.2312)	-0.005(0.004; 0.0590)
Animal vs Music	-0.003(0.003; 0.6979)	-0.006(0.003; 0.0106)	-0.001(0.005; 0.9977)	-0.002(0.004; 0.8142)	-0.005(0.004; 0.0276)	-0.003(0.005; 0.4442)
Animal vs Nature	-0.005(0.003; 0.0574)	-0.007(0.004; 0.0015)	-0.007(0.007; 0.0008)	-0.011(0.006; 0.0001)	-0.010(0.003; 0.0001)	-0.001(0.002; 1.0000)
Animal vs Tools	-0.003(0.001; 0.6174)	-0.003(0.004; 0.3163)	-0.004(0.004; 0.0643)	0.000(0.003; 1.0000)	-0.003(0.003; 0.3687)	-0.004(0.002; 0.1082)
Music vs Nature	-0.002(0.002; 0.9271)	-0.002(0.003; 0.8998)	-0.005(0.002; 0.0147)	-0.007(0.005; 0.0015)	-0.003(0.002; 0.2701)	0.002(0.004; 0.7635)
Music vs Tools	0.000(0.002; 1.0000)	0.002(0.003; 0.9919)	-0.001(0.004; 0.9951)	0.003(0.003; 0.6750)	0.003(0.003; 0.3565)	-0.001(0.004; 1.0000)
Nature vs Tools	0.002(0.002; 0.9719)	0.003(0.003; 0.2907)	0.003(0.002; 0.3035)	0.011(0.003; 0.0001)	0.007(0.001; 0.0017)	-0.004(0.001; 0.2476)
