# Supplementary material for: Intermediate acoustic-to-semantic representations link behavioral and neural responses to natural sounds
Source: Nat Neurosci. 2023 Mar 16;26(4):664–72. doi: 10.1038/s41593-023-01285-9 (PMC10076214; doi:10.1038/s41593-023-01285-9)
Supplement: Supplementary file 2 — Reporting Summary [file 41593_2023_1285_MOESM2_ESM.pdf]

nature portfolio

Corresponding author(s): Bruno L. Giordano and Elia Formisano

Last updated by author(s): Feb 6, 2023

Reporting Summary

Nature Portfolio wishes to improve the reproducibility of the work that we publish. This form provides structure for consistency and transparency in reporting. For further information on Nature Portfolio policies, see our Editorial Policies and the Editorial Policy Checklist.

Statistics

For all statistical analyses, confirm that the following items are present in the figure legend, table legend, main text, or Methods section.

n/a	Confirmed	
☐	☒	The exact sample size (*n*) for each experimental group/condition, given as a discrete number and unit of measurement
☒	☐	A statement on whether measurements were taken from distinct samples or whether the same sample was measured repeatedly
☐	☒	The statistical test(s) used AND whether they are one- or two-sided<br>*Only common tests should be described solely by name; describe more complex techniques in the Methods section.*
☐	☒	A description of all covariates tested
☐	☒	A description of any assumptions or corrections, such as tests of normality and adjustment for multiple comparisons
☐	☒	A full description of the statistical parameters including central tendency (e.g. means) or other basic estimates (e.g. regression coefficient) AND variation (e.g. standard deviation) or associated estimates of uncertainty (e.g. confidence intervals)
☐	☒	For null hypothesis testing, the test statistic (e.g. *F*, *t*, *r*) with confidence intervals, effect sizes, degrees of freedom and *P* value noted<br>*Give P values as exact values whenever suitable.*
☒	☐	For Bayesian analysis, information on the choice of priors and Markov chain Monte Carlo settings
☒	☐	For hierarchical and complex designs, identification of the appropriate level for tests and full reporting of outcomes
☐	☒	Estimates of effect sizes (e.g. Cohen's *d*, Pearson's *r*), indicating how they were calculated

Our web collection on statistics for biologists contains articles on many of the points above.

Software and code

Policy information about availability of computer code

Data collection	Data were collected using custom Matlab code.
Data analysis	Data were analysed using custom Matlab and Python code, as well as publicly available Matlab and Python code for the characterization of the stimulus conditions using computational models (see Methods). Custom analysis code have been deposited at https://doi.org/10.5061/dryad.0p2ngf258

For manuscripts utilizing custom algorithms or software that are central to the research but not yet described in published literature, software must be made available to editors and reviewers. We strongly encourage code deposition in a community repository (e.g. GitHub). See the Nature Portfolio guidelines for submitting code & software for further information.

Data

Policy information about availability of data

All manuscripts must include a data availability statement. This statement should provide the following information, where applicable:

- Accession codes, unique identifiers, or web links for publicly available datasets
- A description of any restrictions on data availability
- For clinical datasets or third party data, please ensure that the statement adheres to our policy

All data used in the analyses are available at the following Dryad repository: https://doi.org/10.5061/dryad.0p2ngf258

Human research participants

Policy information about studies involving human research participants and Sex and Gender in Research.

Reporting on sex and gender	Sex and gender were not considered in the design of the published experiments whose data we re-analysed for this study. In these studies, sex was self-reported by experiment participants; gender information was not collected. These variables were not considered in our analyses because they were beyond the scope of the current study.
Population characteristics	Normal hearing individuals (behavioural experiment: 26 females, 14 males, median age = 25 years; fMRI experiment: 3 females, 2 males, median age = 27 years)
Recruitment	Participants in the behavioural experiment were recruited from mailing lists local to the McGill University. Participants in the fMRI experiment were recruited among graduate students at Maastricht University.
Ethics oversight	The behavioural experiment was approved by the McGill Research Ethics Board. The fMRI experiment was approved by the Ethical Committee of the Faculty of Psychology and Neuroscience of Maastricht University.

Note that full information on the approval of the study protocol must also be provided in the manuscript.

Field-specific reporting

Please select the one below that is the best fit for your research. If you are not sure, read the appropriate sections before making your selection.

☒ Life sciences ☐ Behavioural & social sciences ☐ Ecological, evolutionary & environmental sciences

For a reference copy of the document with all sections, see nature.com/documents/nr-reporting-summary-flat.pdf

Life sciences study design

All studies must disclose on these points even when the disclosure is negative.

Sample size	The study is based on the combined analysis of data from two published datasets: 1) Giordano et al. (2010), behavioural dataset, n participants=40, and 2) Santoro et al. (2018), fMRI dataset, n participants = 5. No statistical methods were used to pre-determine sample sizes in these study. The number of participants in Giordano et al. (2010) was established based on a methodological study on the reliability of hierarchical sorting data (Giordano et al., 2011, Multivariate Research Methods, 46 779-811) so as to have reliable group-aggregate dissimilarity estimates in each of two experimental conditions. The fMRI data set was optimized to conduct decoding analyses at single-subject and single-sound level: for each subject, data were collected using 7 Tesla fMRI, which ensures high functional contrast-to-noise and using a number of sounds (no sounds =288) substantially larger than comparable auditory fMRI studies. Furthermore, each sound was presented 3 times, leading to accurate estimates of single-participant cerebral responses to single sounds. We have shown (Santoro, PNAS 2108) that, combined with non-parametric, permutation-based statistics, these data and sample size are sufficient to detect significant differences between models.
Data exclusions	Participants performed an incidental one-back repetition detection task (6.49% of all sound trials) and responded with a button press when a sound was repeated (fMRI data for one-back trials not considered because of motor contamination and stimulus-habituation effects).
Replication	Strictly speaking, our statistical framework does not rely on the replication of experiments. Instead, it measures the extent to which statistical models explain unseen data not used in model training. This is the core concept of cross-validation, which measures the generalizability of statistical models to unseen data. From this point of view, cross-validation indeed measures the replicability of statistical models. Analyses of behavioural data rely on 100 cross-validation folds that generalize statistical models across separate groups of 50% of the participants. Analyses of fMRI data rely on 40 cross-validation folds across participants and stimulus sets.
Randomization	Participants for the Giordano et al. (2010) behavioural dataset were assigned randomly to experimental conditions. Participants for the Santoro et al. (2017) fMRI dataset were assigned to the same experimental conditions. The explicit analysis of age/gender etc. participant-related covariates in these datasets was beyond the scope of the current study, as our statistical framework sought to explicitly generalize across diverse groups of participants.
Blinding	In each of the two conditions of the the behavioural experiment and in the fMRI experiment all participants were exposed to the same experimental stimuli and paradigm. The assignment of participants to the two conditions of the behavioural experiment was established randomly. No blinding was required in the investigators. No blinding was carried out in the data analysis, for which data from all experiments and for all conditions were analyzed equally, within the same pipeline, and without discarding any data from the previously published experiments from which the data in this study were sourced.

Reporting for specific materials, systems and methods

We require information from authors about some types of materials, experimental systems and methods used in many studies. Here, indicate whether each material, system or method listed is relevant to your study. If you are not sure if a list item applies to your research, read the appropriate section before selecting a response.

Materials & experimental systems

n/a	Involved in the study
☒	☐ Antibodies
☒	☐ Eukaryotic cell lines
☒	☐ Palaeontology and archaeology
☒	☐ Animals and other organisms
☒	☐ Clinical data
☒	☐ Dual use research of concern

Methods

n/a	Involved in the study
☒	☐ ChIP-seq
☒	☐ Flow cytometry
☐	☒ MRI-based neuroimaging

Magnetic resonance imaging

Experimental design

Design type

Event-related design

Design specifications

The 288 fMRI stimuli were divided into four non-overlapping sets of 72 sounds each. Grouping was performed randomly under the constraint that all semantic categories would be equally represented in each set. Each subject underwent two scan sessions. During one session, two of the four sets of stimuli were presented. The order of the stimulus sets was counterbalanced across subjects. Each session consisted of six functional runs (?11 min each). We presented one stimulus set (72 distinct sounds) per run, and every set was presented three times (i.e., three runs per set). Within each run, stimuli were arranged according to a pseudorandom scheme to ensure that all semantic categories would be uniformly distributed throughout the run and that no stimuli of the same category would follow each other. Within each scan session, the stimulus sets were presented in an interleaved fashion. Within each run, stimuli were presented in the silent gap between acquisitions with a randomized interstimulus interval of two, three, or four TRs (TR = 2600 ms).

Behavioral performance measures

The fMRI data were acquired during a passive-listening experiment with five catch trials per run (i.e., trials in which the preceding sound was repeated). Subjects were instructed to respond with a button press when a sound was repeated. Catch trials were excluded from the analysis.

Acquisition

Imaging type(s)

Functional

Field strength

7T

Sequence & imaging parameters

T2*-weighted functional data were acquired using a clustered echo planar imaging sequence in which time gaps were placed after the acquisition of each volume. The fMRI time series were acquired according to a fast event-related scheme, with the following acquisition parameters: TR = 2,600 ms, TA = 1,200 ms, TE = 19 ms, GRAPPA = 2, partial Fourier = 6/8, flip angle = 70°, voxel size = 1.5 × 1.5 × 1.5 mm3. Nslices = 46. There was no gap between slices.

Area of acquisition

The acquisition volume covered the brain transversally from the inferior portion of the anterior temporal pole to the superior portion of the STG bilaterally.

Diffusion MRI ☐ Used ☒ Not used

Preprocessing

Preprocessing software

Functional and anatomical data were preprocessed with BrainVoyager QX (Brain Innovations). No spatial smoothing was applied. Anatomical data from the two scan sessions were aligned using the automatic alignment in BrainVoyager QX.

Normalization

Functional slices were coregistered to the anatomical data and normalized in Talairach space. Normalized functional data were resampled (sinc interpolation) to 1-mm isotropic resolution. The border between gray and white matter was segmented from anatomical volumes and used to generate cortical surface meshes of the individual subjects. We performed cortex-based alignment of all subjects. Alignment information was used to obtain a group surface mesh representation.

Normalization template

original Talairach

Noise and artifact removal

Preprocessing consisted of temporal high-pass filtering (removing drifts of seven cycles or less per run) and 3D motion correction (trilinear/sinc interpolation). Anatomical data from the two scan sessions were aligned using the automatic alignment in BrainVoyager QX.

Volume censoring

No volume censoring was applied

Statistical modeling & inference

Model type and settings: RSA analyses on group-averaged ROI-specific RDMs. The RSA framework considered in our analyses generalizes model representation results from group-averaged data in the training group of participants to group-averaged data in the test group of participants (multiple splits considered). As such, the statistical approach shares traits with both the fixed-effects analysis framework (because we consider model-representations in group-averaged data), and with the random-effects analysis framework (because large interindividual differences would make the generalization from training to test set hard if not impossible).

Effect(s) tested: We assessed the representation of multiple computational models of sound processing in fMRI data collected with a condition-rich design.

Specify type of analysis: ☐ Whole brain ☒ ROI-based ☐ Both

Anatomical location(s): Anatomical ROIs were manually outlined on the cortex reconstruction of each individual subject using BrainVoyager QX (Brain Innovations). We obtained 3D ROIs by projecting the selected regions into the volume space of the same subjects.

Statistic type for inference (See Eklund et al. 2016): Permutation-based inference.

Correction: Multiple comparison corrections adjusting for family-wise error rate at the 0.05 level, relying on a maximum-statistics permutation-based approach.

Models & analysis

n/a	Involved in the study
☒	☐ Functional and/or effective connectivity
☒	☐ Graph analysis
☐	☒ Multivariate modeling or predictive analysis

Multivariate modeling and predictive analysis: We considered the betas from the GLM models of the fMRI time series as independent variables. Betas were extracted within each ROI for each participant, and analysed within a cross-validated RSA framework. No dimension reduction was required neither on the side of the fMRI data, nor on the side of the computational models. All statistical models of the representation of computational models in fMRI (and behavioural) data were trained and evaluated (tested) on separate groups of participants (evaluation metric = cross-validated RSQ).
